# Diversity and evolution of multiple *orc/cdc6*-adjacent replication origins in haloarchaea

**DOI:** 10.1186/1471-2164-13-478

**Published:** 2012-09-14

**Authors:** Zhenfang Wu, Hailong Liu, Jingfang Liu, Xiaoqing Liu, Hua Xiang

**Affiliations:** 1 State Key Laboratory of Microbial Resources, Institute of Microbiology, Chinese Academy of Sciences, Beijing 100101, China

## Abstract

**Background:**

While multiple replication origins have been observed in archaea, considerably less is known about their evolutionary processes. Here, we performed a comparative analysis of the predicted (proved in part) *orc/cdc6*-associated replication origins in 15 completely sequenced haloarchaeal genomes to investigate the diversity and evolution of replication origins in halophilic Archaea.

**Results:**

Multiple *orc/cdc6*-associated replication origins were predicted in all of the analyzed haloarchaeal genomes following the identification of putative ORBs (origin recognition boxes) that are associated with *orc/cdc6* genes. Five of these predicted replication origins in *Haloarcula hispanica* were experimentally confirmed via autonomous replication activities. Strikingly, several predicted replication origins in *H. hispanica* and *Haloarcula marismortui* are located in the distinct regions of their highly homologous chromosomes, suggesting that these replication origins might have been introduced as parts of new genomic content. A comparison of the origin-associated Orc/Cdc6 homologs and the corresponding predicted ORB elements revealed that the replication origins in a given haloarchaeon are quite diverse, while different haloarchaea can share a few conserved origins. Phylogenetic and genomic context analyses suggested that there is an original replication origin (*oriC1*) that was inherited from the ancestor of archaea, and several other origins were likely evolved and/or translocated within the haloarchaeal species.

**Conclusion:**

This study provides detailed information about the diversity of multiple *orc/cdc6*-associated replication origins in haloarchaeal genomes, and provides novel insight into the evolution of multiple replication origins in Archaea.

## Background

DNA replication is an essential process and is generally conserved across all three domains of life, making use of two different DNA replication apparatuses (bacterial-type and eukaryotic-type) [1,2]. DNA replication initiates from a single origin in bacteria, whereas multiple origins are utilized in eukaryotes [3]. The study of replication origins in archaea has been ongoing for more than a decade, and multiple replication origins have been identified in several archaeal species [4-10]. It is not yet understood, however, why archaea adopt multiple origins to initiate replication of their bacterial-like chromosomes or how these multiple replication origins evolved. Notably, answering these questions may also provide insight into the mechanisms of the more complex replication origins found in eukaryotes.

Our current knowledge of archaeal replication origins comes from a few model strains, including *Pyrococcus abyssi* (Euryarchaeota) [4,11,12], *Sulfolobus* spp. (Crenarchaeota) [5,6,13], and two halophilic archaea (Euryarchaeota), *Haloferax volcanii*[9] and *Halobacterium* sp. strain NRC-1 [10,14]. A single origin was mapped near the only *orc1* gene in the genome of *P. abyssi* via a combination of *in silico*[4] and biochemical [11,12] analyses. Three origins were mapped in *Sulfolobus solfataricus* and *Sulfolobus acidocaldarius* by means of microarray-based marker frequency analysis (MFA) [6], and two origins adjacent to the *orc/cdc6* genes were previously identified by two-dimensional gel electrophoresis [5]. On the chromosome of *Halobacterium* sp. NRC-1, one origin was verified with autonomous replication activity [14], and four were mapped using whole-genome MFA, with three origins located in the vicinity of *orc/cdc6* genes [10]. In *H. volcanii*, five replication origins were identified, two within the chromosome and one each within the three megaplasmids pHV1, pHV3 and pHV4 [9].

These experimental data revealed that the basic structure of replication origins is conserved among archaea, normally containing an AT-rich unwinding element and several conserved repeats (Origin Recognition Box, ORB) [9]. The ORB elements were proven to be the recognition sites for the Orc/Cdc6 initiation protein via biochemical [5] and structural approaches [15,16]. In addition, distinct from the ORBs identified in the *oriC1* of *S. solfataricus*[5], a halophile-specific “G-string” (long G-stretches locating at the end of ORBs) was observed in all origins from *H. volcanii*[9]. Whereas the Cdc6 and the ORC complex proteins (Orc1-6) act together to recruit the MCM (minichromosome maintenance) complex to an origin of replication in eukaryotes [3,17], a subset of initiator proteins (Orc/Cdc6), which are related to both Orc1 and Cdc6 of eukaryotes, were adopted by archaea. Therefore, archaeal Orc/Cdc6 is considered to possess both origin recognition and MCM-loading activities [3]. Previous studies in *S. solfataricus* revealed that origin identity was determined by the specific recognition of Orc/Cdc6 proteins [18]. Interestingly, the multiple origins, especially the ORB sequences and their associated Orc/Cdc6 proteins, are quite diverse in all three experimentally characterized archaea (*S. solfataricus*, *H. volcanii* and *Halobacterium* sp. NRC-1) [5,9,10], indicating independent evolutionary history. In particular, an origin comparison between two hyperthermophilic archaeal genera, *Aeropyrum* and *Sulfolobus*, suggests that the capture of extrachromosomal elements accounts for replicon evolution [7]. However, as the study of replication origins has been limited to only a few archaeal species, it is still difficult to determine the evolutionary relationship of multiple replication origins within Archaea.

Haloarchaeal genomes are normally composed of multiple replicons (chromosome, minichromosome, and plasmids) with multiple Orc/Cdc6 homologs (usually more than 10 homologs) [19-30], indicating that the occurrence of multiple replication origins is widespread in haloarchaea. To date, however, studies have been limited to two model haloarchaea, *H. volcanii*[9] and *Halobacterium* sp. NRC-1 [10]. There is not enough information to understand the diversity and evolution of multiple replication origins in this distinct group of archaea. In this work, which is based on both previous experimental data and the identification of replication origins in *Haloarcula hispanica*, a haloarchaeon that was recently sequenced in our laboratory [20], we performed a comparative analysis of predicted *orc/cdc6*-associated replication origins in 15 completely sequenced haloarchaeal genomes. These comparative analyses indicated that the introduction of novel replication origins usually accompanied the acquisition of new genomic content by insertions into a chromosome or the reconstruction of novel extrachromosomal replicons, which may be linked to an adaptive mechanism of haloarchaea to diverse environments, similar to *Salinibacter ruber*[31]. In particular, various families of *orc/cdc6*-associated replication origins were identified, and different evolutionary mechanisms, including ancestral preservation, translocation among haloarchaea and likely differential loss, were proposed to account for the current multiple origins of replication in the haloarchaeal genomes.

## Results and discussion

### Identification of *orc/cdc6*-associated replication origins in *H*. *hispanica*

Research in archaeal model strains indicates that most replication origins share conserved characteristics, such as the presence of inverted ORB elements and being located directly adjacent to *orc/cdc6* genes [5,9,10]. In addition, a “G-string” at the end of ORB elements was observed in all identified origins from *H. volcanii*[9]. These common features provided us a reference standard to predict replication origins in *H. hispanica*. Briefly, only those intergenic regions (IRs) that contain ORB-like elements and are directly adjacent to *orc/cdc6* genes were considered to be putative *orc/cdc6*-associated replication origins. Necessarily, although they were not included in the scope of this study, we do not exclude the possibility of replication origins that are not directly adjacent to *orc/cdc6* genes or are without classical ORB-like elements. Replication origins with these characteristics were shown to exist in *Sulfolobus* spp. [5] and may exist in *Halobacterium* sp. NRC-1 [10]; however, to our knowledge, they constitute only a small proportion of the replication origins in archaea and are not easily predicted with current information.

**Figure 1:**
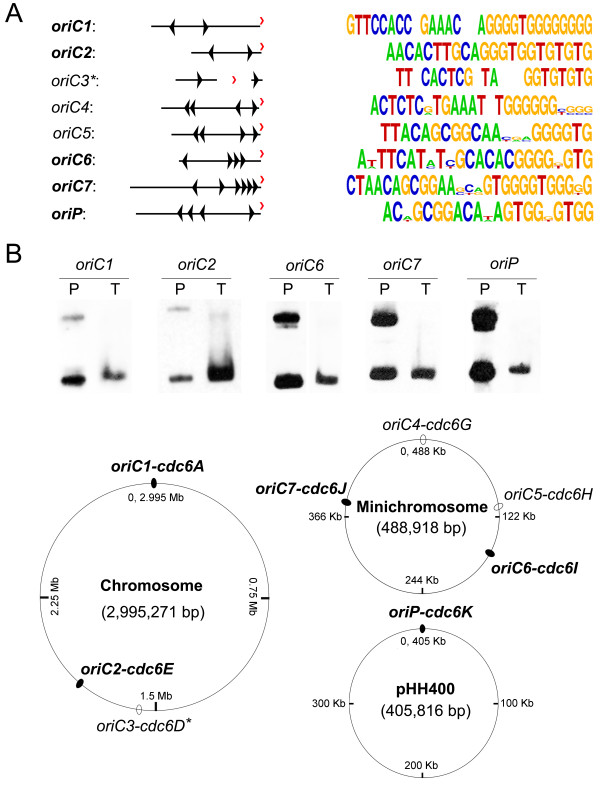
**Bioinformatic and genetic identification of replication origins in *H. hispanica. *A.** Seven replication origins, *oriC1*-*cdc6A* and *oriC2*-*cdc6E* in the main chromosome; *oriC4*-*cdc6G*, *oriC5*-*cdc6H*, *oriC6*-*cdc6I* and *oriC7*-*cdc6J* in the minichromosome; and *oriP*-*cdc6K* in the megaplasmid, were predicted by searching ORB motifs (indicated with small triangles) in the IRs located directly adjacent to *orc/cdc6* genes (indicated with red arrowheads) using MEME software. Logo representations of ORB elements are presented on the right, and the spaces represent sequences that are not conserved. oriC3*: predicted deficient origin adjacent to *cdc6D* gene. **B.** Replication assay for plasmids containing the origins predicted in A. (Up) Southern blot analysis with a *bla* gene probe: lane T contains crude DNA extracted from the *H. hispanica* transformants, and lane P represents the purified plasmid as an input control; (down) summaries of the identification of origins in *H. hispanica* and the five origins with ARS activity (*oriC*1, 2, 6, 7, P) are indicated with filled ovals and are bolded in A.

To identify replication origins in *H. hispanica*, the IRs around the *orc/cdc6* genes were examined for the presence of ORB elements. *H. hispanica* encodes eleven *orc/cdc6* genes, with six copies (*cdc6AF*) in the main chromosome, four (*cdc6GJ*) in the minichromosome and only one (*cdc6K*) in the megaplasmid. ORB repeats harboring a G-rich motif were observed adjacent to eight *orc/cdc6* genes (Additional file 1 and Figure 1A), in agreement with the halophile-specific “G-string” elements found in *H. volcanii*[9]. However, in contrast to other characterized archaeal origins with at least two ORB repeats flanking an AT-rich unwinding element, only one ORB-like element was observed in each IR flanking the *cdc6D* gene, which was considered to be a deficient origin (oriC3-cdc6D*) when examined by hand (Figure 1A and Additional file 1). Accordingly, seven replication origins were predicted in *H. hispanica*: two were in the main chromosome (*oriC1cdc6A* and *oriC2cdc6E*), four were in the minichromosome (*oriC4cdc6G*, *oriC5cdc6H*, *oriC6cdc6I* and *oriC7cdc6J*), and one was in the megaplasmid (*oriPcdc6K*) (Figure 1A and B).

To confirm these putative replication origins, we performed a genetic assay to test their autonomous replication activities. As a control, we also examined whether oriC3-cdc6D* and IRs around *cdc6B*, *cdc6C* and *cdc6F*, where no ORBs were detected, could engage in origin activities. DNA fragments, including the *orc/cdc6* genes plus their flanking IRs, were cloned into a nonreplicating plasmid, pBI101 [32,33], to assay for the presence of an autonomously replicating sequence (ARS) (Figure 1, Additional file 2). Of the eleven *orc/cdc6* genes with adjacent IRs, *oriC1cdc6A* and *oriC2cdc6E* in the main chromosome, *oriC6cdc6I* and *oriC7cdc6J* in the minichromosome and *oriP-cdc6K* in the megaplasmid were able to confer replication ability to the non-replicating plasmid (Figure 1B, Additional file 2), which was indicative of the ARS activities of these origins. As expected, no replicating ability was observed for plasmids constructed with oriC3-cdc6D* or with the fragments containing *cdc6B*, *cdc6C* and *cdc6F* (Additional file 2). Although the remaining two predicted replication origins, *oriC4-cdc6G* and *oriC5-cdc6H*, shared a conserved structure with characteristic archaeal origin (Additional file 1), they could not drive the autonomous replication activities under our experimental conditions, which is reminiscent of the dormant origins found in eukaryotes [34]. Dormant replication origins are normally inactive, but they can be activated for cellular response to replicative stress [35,36]. In the future, it would be interesting to further analyze the utilization of these likely dormant replication origins in *H. hispanica*.

### Most *orc/cdc6* genes are predicted to associate with replication origins in haloarchaea

To date, the genomes of 15 haloarchaea have been made available through NCBI (before October 2011), and 14 of these 15 genomes include the minichromosomes and/or megaplasmids, which provided us the opportunity to perform a comparative genomic analysis of replication origins in haloarchaea. To focus on the *orc/cdc6*-associated replication origins, we first conducted an exhaustive search of the *orc/cdc6* genes in the 15 sequenced haloarchaeal genomes (Table 1).

**Table 1:** Predicted origin-associated Orc/Cdc6 homologs in the haloarchaeal genomes

**Organism**^1^	**genome**	**Cdc6**^2^	***ori*-associated Cdc6**^3^	**Percentage**^4^
*Halalkalicoccus jeotgali* B3 [19] (CP002062-68)	Chromosome	4	2	50%
	plasmid 1	3	2	67%
	plasmid 2	3	3	100%
	plasmid 3^#^	0		
	plasmid 4^#^	0		
	plasmid 5^#^	0		
*Haloarcula hispanica*[20] (CP002921-23)	Chromosome	6	2	33%
	Chromosome II	4	4	100%
	pHH400	1	1	100%
*Haloarcula marismortui*[21] (AY596290-98)	Chromosome	7	4	57%
	Chromosome II	2	2	100%
	pNG700	1	1	100%
	pNG600	2	1	50%
	pNG500	2	2	100%
	pNG400*	0		
	pNG300*	1	0	
	pNG200*	0		
	pNG100	1	1	100%
*Halobacterium salinarum* R1^5^[22] (AM774415-19)	Chromosome	4	3	75%
	pHS3	2	1	50%
	pHS2	4	3	75%
	pHS1*	2	1	50%
	pHS4*	0		
*Halobacterium* sp. NRC-1 ^6^[23] (AE004437-38; AF016485)	Chromosome	4	3	75%
	pNRC200*	4	2	50%
	pNRC100*	1	0	
*Haloferax volcanii*^6^[24] (CP001953-57)	Chromosome	8	4	50%
	pHV4	4	3	75%
	pHV3	1	1	100%
	pHV2^#^[21]	0		
	pHV1	2	2	100%
*Halogeometricum borinquense*[25] (CP001690-95)	Chromosome	5	2	40%
	pHBOR01	1	1	100%
	pHBOR02	2	1	50%
	pHBOR03	2	2	100%
	pHBOR04	1	1	100%
	pHBOR05^#^	0		
*Halomicrobium mukohataei*[26] (CP001688-89)	Chromosome	3	1	33%
	pHmuk01	1	1	100%
*Halopiger xanaduensis* (CP002839-42)	Chromosome	9	5	56%
	pHALXA01	1	1	100%
	pHALXA02	1	1	100%
*Haloquadratum walsbyi*[27] (AM180088-89)	Chromosome	5	2	40%
	pL47^#^	0		
*Halorhabdus utahensis*[28] (CP001687)	Chromosome	5	2	40%
*Halorubrum lacusprofundi* (CP001365-67)	Chromosome	5	3	60%
	Chromosome II	5	4	80%
	pHLAC01*	5	4	80%
*Haloterrigena turkmenica*[29] (CP001860-66)	Chromosome	11	7	64%
	pHTUR01	1	1	100%
	pHTUR02	1	1	100%
	pHTUR03^#^	0		
	pHTUR04	3	3	100%
	pHTUR05^#^	0		
	pHTUR06^#^	0		
*Natrialba magadii* (CP001932-35)	Chromosome	7	5	71%
	pNMAG01	1	1	100%
	pNMAG02	1	1	100%
	pNMAG03^#^	0		
*Natronomonas pharaonis*[30] (CR936257-59)	Chromosome	4	2	50%
	pL131	1	1	100 %
	pL23^#^	0		

^1^ Haloarchaeal genomes and their GenBank accession numbers. ^2^ Number of annotated Orc/Cdc6 proteins; only those greater than 300 amino acids are included. ^3^ Number of Orc/Cdc6 proteins associated with predicted replication origins. ^4^ Percentage of predicted origin-associated Orc/Cdc6 proteins. ^5^ The chromosomes of *Halobacterium salinarum* R1 are almost identical to those of *Halobacterium* sp. NRC-1, showing overlapped predicted replication origins of three origins in the chromosome and one each origin in pHS2 and pHS3. ^6^ Origins were experimentally mapped in these haloarchaea. * *Rep* genes encoded in these replicons might be responsible for DNA replication initiation. ^#^ Both *orc/cdc6* and *rep* genes are absent in these replicons. A gene (Hvo_D0003) in pHV2 of *H. volcanii* shows low homology with the *repH* gene.

Multiple Orc/Cdc6 homologs are encoded in each of the 15 sequenced haloarchaeal genomes. Based on a previous study [15], origin-associated Orc/Cdc6 proteins contain two important domains, a N-terminal AAA + domain and a C-terminal winged-helix domain, and almost all have a length greater than 300 amino acids. A total of 154 Orc/Cdc6 homologs fulfilling these criteria were collected from the 15 sequenced haloarchaeal genomes (Table 1 and Additional file 3), and the IRs flanking these *orc/cdc6* genes were collected for motif searching. Interestingly, distinct ORB-like elements harboring G-string were found in the IRs flanking nearly two-thirds (102 of 154) of the *orc/cdc6* genes (Table 1 and Additional files 3 and 4), and the predicted replication origins were rechecked manually to remove deficient origins such as oriC3-cdc6D* in *H. hispanica*. As expected, multiple replication origins were predicted in all of the analyzed haloarchaeal genomes (Table 1). *Haloterrigena turkmenica* has the greatest number of predicted origins at 12, and 7 of those origins are located on its chromosome (Table 1). On average, within the haloarchaeal chromosomes, more than half of the *orc*/*cdc6* genes have predicted origins nearby: a maximum of 75% (3 of 4) in *Halobacterium* spp. and a minimum of 33% (1 of 3) in *Halomicrobium mukohataei* (Table 1). Compared with the chromosome, the overwhelming majority (greater than 80%) of the *orc*/*cdc6* genes in the extrachromosomal elements (minichromosomes and megaplasmids) are associated with predicted replication origins (Table 1).

As several replication origins have been experimentally mapped in *H. hispanica* (Figure 1), *H. volcanii*[9] and *Halobacterium* sp. NRC-1 [10], these experimental data were used to evaluate the efficiency of the origin prediction performed in this study. In *H. hispanica*, five out of the seven predicted replication origins were confirmed to have ARS activity. For the replication origins in the chromosome of *Halobacterium* sp. NRC-1, a high consistency between our predicted results and the whole-genome MFA [10], except for one uncertain origin (*oriC4*), proves the efficiency of the bioinformatic approach in this study. For *H. volcanii*, in addition to the five replication origins that were previously genetically mapped [9], five additional replication origins were also predicted in this study. As discussed above, these additional predicted origins might be weak or dormant replication origins, which are not easily identified by experimental approaches.

In summary, our bioinformatic approach not only is important for identifying active replication origins in haloarchaea but also provides novel information for predicting likely dormant replication origins, which is also important for the future study of replication regulation and adaptation in archaea.

### Diversity of *orc/cdc6*-associated replication origins in haloarchaea

**Figure 2:**
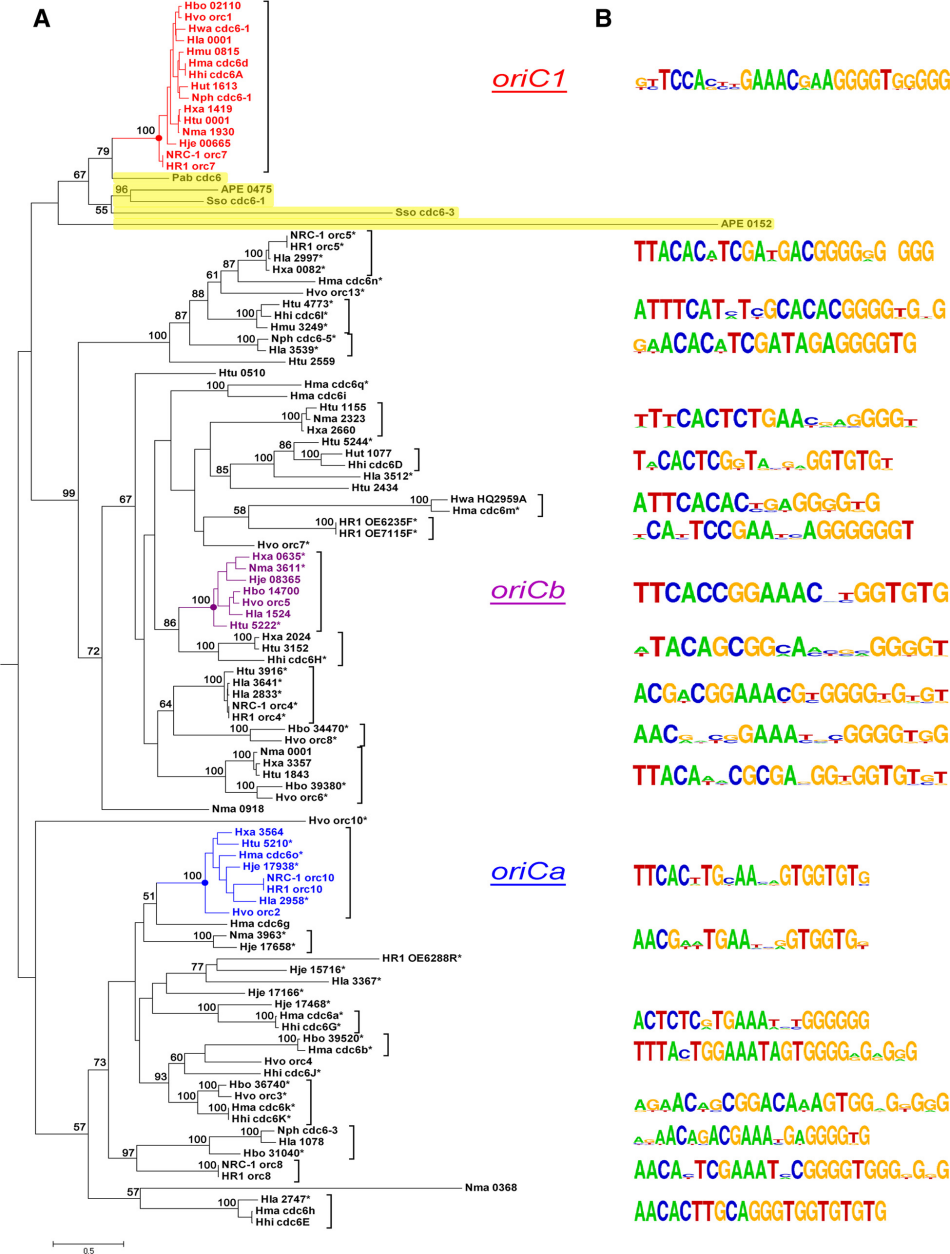
**Families of *orc/cdc6*-associated replication origins in the haloarchaeal genomes. A.** Phylogenetic tree of origin-associated Orc/Cdc6 homologs (Hbo: *Halogeometricum borinquense*, Hhi: *Haloarcula hispanica*, Hje: *Halalkalicoccus jeotgali* B3, Hla: *Halorubrum lacusprofundi*, Hma: *Haloarcula marismortui*, Hmu: *Halomicrobium mukohataei*, HR1: *Halobacterium salinarum* R1, Htu: *Haloterrigena turkmenica*, Hut: *Halorhabdus utahensis*, Hvo: *Haloferax volcanii* DS2, Hwa: *Haloquadratum walsbyi*, Nma: *Natrialba magadii*, Nph: *Natronomonas pharaonis*, NRC-1: *Halobacterium* sp. NRC-1). * indicates the Orc/Cdc6 proteins encoded on extrachromosomal elements. Orc/Cdc6 proteins from other archaea (APE: *Aeropyrum pernix*, Pab: *Pyrococcus abyssi*, Sso: *Sulfolobus solfataricus*) are highlighted with yellow background. The origin conserved in all genomes was assigned the name *oriC1*, as in previous reports (in red), and the other two origin clusters with the top two members, excluding *oriC1* in this study, were assigned the names *oriCa* (in blue) and *oriCb* (in pink). **B.** Logo representations of the putative ORB elements identified in the predicted replication origins adjacent to the *orc/cdc6* genes. The ORB elements are not shown for origin families with only one member.

A recent report suggested that Orc/Cdc6 initiators specifically determine origin discrimination in archaea [18]. To investigate this further, a phylogenetic analysis of *ori*-associated Orc/Cdc6 proteins in haloarchaea was performed, and the resulting tree showed that Orc/Cdc6 homologs cluster into different families (Figure 2A), which suggested that various *orc/cdc6*-associated replication origins have been adopted in haloarchaea. Different Orc/Cdc6 families have been suggested in previous work [14,37]; herein, we focused on the putative origin-associated Orc/Cdc6 homologs with the intention of providing a detailed classification of predicted replication origins. Although setting precise boundaries was difficult, the predicted replication origins could be sorted into distinct families based on a combination of the phylogenetic tree of the Orc/Cdc6 homologs (Figure 2A) and a comparison of ORB sequences (Figure 2B). It is noteworthy that BLAST analyses confirmed that only those Orc/Cdc6 homologs showing high identities (at least 80%) were grouped into the same family in this study. Specifically, the origins adjacent to the specific Orc/Cdc6 conserved among all haloarchaea were named *oriC1*, as previously reported [10,37], and two other families with the top two members were selected and named *oriCa* and *oriCb* to facilitate additional evolutionary analyses (Figure 2A). This classification of replication origins will become more complete when more haloarchaeal genomes become available and will aid in the understanding of replication origins in novel haloarchaea.

Notably, Orc/Cdc6 proteins associated with *oriC1* not only are conserved in all haloarchaeal genomes but were also identified in other archaeal genomes (Figure 2A). For instance, Cdc6-1 in *S. solfataricus* was experimentally proven to recognize the ORB elements of *oriC1* in *Halobacterium* sp. NRC-1 [5]. Thus, we suggest that this conserved origin (*oriC1*) might be present in an archaea ancestor and maintained in different lineages during the evolutionary history of Archaea. By contrast, other Orc/Cdc6 homologs from different haloarchaeal genomes could be clustered into several distinct families (Figure 2A), indicating that these origins are shared by different haloarchaea. As different Orc/Cdc6 proteins from the same haloarchaeal genome are normally distributed into different families (Figure 2A), the results suggested that multiple origins within a haloarchaeon were unlikely to arise from internal duplications.

To further characterize these predicted origins in haloarchaea, ORB sequences were extracted from all of the putative origins (Additional file 4) for comparison analysis (Figure 2B). The results revealed the linkage-specificity of Orc/Cdc6 homologs and the corresponding ORB elements, (i.e., the predicted ORB sequences proximal to Orc/Cdc6 homologs within the same family are highly similar) (Figure 2B and Additional file 5). These observations suggested that Orc/Cdc6 proteins specifically recognize adjacent ORB elements, consistent with a recent report about origin discrimination by Orc/Cdc6 initiators [18].

Notably, the structures of origins from even the same family are also diverse in haloarchaea. Although the predicted replication origins are primarily located directly upstream of *orc/cdc6* genes, as previously observed [4,5,9,10], there are a few exceptions (Additional file 4) in which ORB elements are located in IRs downstream of the *orc/cdc6* gene (e.g., proximal to *cdc6n* in *H. marismortui*, *orc5* in *Halobacterium* species, *orc8* in *H. volcanii* and *Nmag_3611* in *N. magadii*) (Additional file 4). In addition, in some predicted replication origins, ORB elements are occasionally observed in both IRs flanking the *orc/cdc6* gene, such as those flanking *cdc6E* in *H. hispanica*, *cdc6h*, *cdc6g* and *cdc6m* in *H. marismortui*, *orc10* and *orc8* in *Halobacterium* species, *orc4* in *H. volcanii* and *Hlac_1078*, *Hlac_2747* and *Hlac_2997* in *H. lacusprofundi* (Additional file 4). Interestingly, although a previous genetic experiment found that the predicted origin proximal to *NRC-1_orc8* is not able to promote efficient autonomous replication [14], this origin, in combination with the origin proximal to *NRC-1_orc10*, was experimentally proven to be active in *Halobacterium* sp. strain NRC-1 by whole-genome MFA [10]. The origin proximal to *orc10* in *Halobacterium* species has a different structure comparing to others in the *oriCa* family (Additional file 4), suggesting that structurally diverse origins function in different haloarchaea.

**Figure 3:**
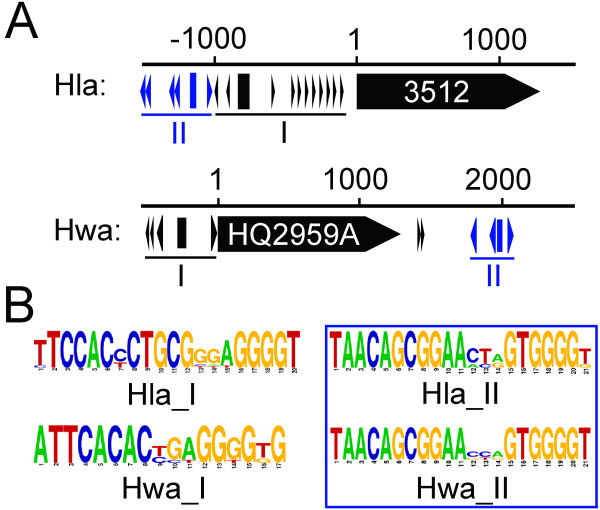
**Two different candidate replication origins are adjacent to one *orc/cdc6 *gene (Hla_3512 or Hwa_HQ2959A). A.** The sequence features of the two putative origins. The *orc/cdc6* genes are indicated with black-boxed arrows, and their start site is numbered 1. The adjacent origins (I and II) were mapped with small arrowheads and rectangles indicating the ORB elements and AT-rich regions, respectively. Origin II, in both cases, contains conserved ORB elements, which are highlighted in blue. **B.** Logo representations of the ORB elements in the four candidate replication origins. The ORB elements (boxed) are highly conserved in Origin II in both cases.

Surprisingly, two different ORB-like elements were found in the IRs proximal to one *orc/cdc6* gene, in either the megaplasmid of *H. lacusprofundi* (Hla_3512) or the main chromosome of *H. walsbyi* (Hwa_HQ2959A) (Figure 3A). One ORB-like element, TAACAGCGGAAACAGTGGGGTGGGGGGGT, is shared by these two different origins, while the other shows no similarity (Figure 3B).

Taken together, the multiple replication origins in haloarchaea are dramatically diverse. In addition to the diversity of the ORB elements and corresponding *orc/cdc6* genes, the number and position of ORB elements also contributes to the diversity of the origins in haloarchaea. This diversity may facilitate the differential utilization of multiple replication origins in haloarchaea. Strikingly, the origin proximal to *orc10* in *Halobacterium* species was active *in vivo*[10], while its conserved origin (*oriCa* family) proximal to *orc2* in *H. volcanii* was not proven functional [9]. As the two origins exhibit different structures and these two haloarchaea grow in different environmental conditions, these observations may provide novel insight into differential utilization of replication origins in haloarchaea.

### Novel replication origins accompany newly acquired genomic content

**Figure 4:**
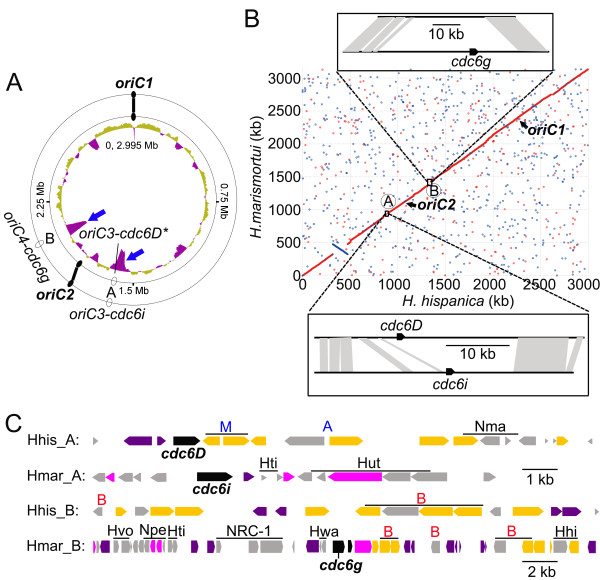
**Comparative analysis of the *orc/cdc6*-associated replication origins between the chromosomes of *H. hispanica *and *H. marismortui. *A.** Distribution of the candidate *orc/cdc6*-associated replication origins in the chromosomes of *H. hispanica* (inside) and *H. marismortui* (outside). G + C content of the chromosome of *H. hispanica* was plotted, and significant variations in the two divergent regions are indicated with blue arrows. The predicted *orc/cdc6*-associated replication origins are indicated as ovals on the chromosome circle, and the shared *orc/cdc6*-associated replication origins in the two *Haloarcula* species, *oriC1* and *oriC2*, are highlighted as filled ovals. **B.** Genome alignment of the chromosomes of *H. hispanica* and *H. marismortui*. Their shared *orc/cdc6*-associated replication origins are indicated as in A. Regions A and B represent discrepancies between the two chromosomes, which are exactly in accordance with the positions of their specific *orc/cdc6*-associated replication origins; oriC3-cdc6D* of *H. hispanica* and *oriC3-cdc6i* of *H. marismortui* are located in region A, and *oriC4-cdc6g* of *H. marismortui* is located in region B. The divergent regions and the edges of the similar regions were confirmed by BLASTN alignments of sequences, and shaded regions denote a similarity of over 70%. Linearized scaled bars are provided. **C.** A schematic representation of the two divergent regions (1 kb scale for Hhis_A, Hmar_A and Hhis_B; 2 kb scale for Hmar_B) between the two chromosomes. The *orc/cdc6* genes are indicated. The polysaccharide biosynthesis genes are in yellow, transposase genes in purple, other genes with known functions in pink and hypothetical genes in gray. The species with the closest matches in the BLAST analysis is indicated on top of the gene: M, Methanobacterium; A, other non-halophilic archaea; B, eubacteria (the colors are designed to correspond to the marks in Additional file 6). The genes in clusters are also in clusters in other haloarchaea, as indicated at the top of the clusters.

As described above, the replication origins of two *Haloarcula* species, *H. hispanica* and *H. marismortui* were predicted, and their ARS activities were also examined in *H. hispanica* (Figure 1). Although their chromosomes show a high degree of conservation (Figure 4B), the two species harbor several different replication origins (Table 1 and Figure 4A). Thus, an in-depth study of these origins would be helpful in understanding the processes involved in the diversity of haloarchaeal replication origins.

With the exception of the two shared replication origins, *oriC1cdc6A* and *oriC2-cdc6E* in *H. hispanica* and the corresponding *oriC1-cdc6d* and *oriC2-cdc6h* in *H. marismortui*, there are one or two other predicted origins specific to each strain: oriC3-cdc6D* in *H. hispanica*, *oriC3-cdc6i* and *oriC4-cdc6g* in *H. marismortui* (Figure 4A and B). The two shared origins, *oriC1* and *oriC2*, were likely present in the ancestor of *Haloarcula*, and their specific origins, oriC3cdc6D* in *H. hispanica* and *oriC3cdc6i* and *oriC4cdc6g* in *H. marismortui*, may have been acquired later through translocation processes following the divergence of these species. An alternative hypothesis is that all three species-specific origins were also present in the ancestor of *Haloarcula* but were lost differently in *H. hispanica* and *H. marismortui*. However, these three predicted origins (oriC3cdc6D*, *oriC3cdc6i* and *oriC4cdc6g*) are located in two divergent regions (region A and B in Figure 4A and B) with significant G + C content variations (Figure 4A), which is indicative of newly acquired genomic content specific to each of the two strains [38]. Thus, the most likely explanation is that these predicted species-specific origins were newly acquired as a part of new genomic content (i.e., the haloarchaeal genomes might recruit novel replication origins accompanying new genes). This hypothesis is reinforced by the abundance of transposases observed around these specific origins (Figure 4C and Additional file 3).

Concentrating on the genes with annotated functions, except for those predicted to be transposases, the majority of genes within the two divergent regions were found to be involved in polysaccharide biosynthesis (Figure 4C). Subsequently, a BLAST analysis against the NCBI non-redundant proteins database was performed on all of the genes in regions A and B in both chromosomes (Figures 4B and C and Additional file 6). The genes were conserved across several different organisms (Figure 4C); most were similar to other haloarchaeal homologs, but for several genes, their closest homologs were outside of haloarchaea. The two linked glycosyltransferase genes in region A of *H. hispanica* were most similar to those found in *Methanobacterium* (Figure 4C and Additional file 6). Several genes in region B of both chromosomes showed the greatest similarity to genes found in bacteria, especially a cluster in region B of *H. hispanica* (Figure 4C and Additional file 6). In addition, those genes found in clusters in the two *Haloarcula* species were also usually found in clusters in other organisms (Figure 4C), suggesting that these genes were acquired in clusters.

A previous report in *Salinibacter ruber* suggested that genes with related functions but different origins might have been assembled together and introduced concurrently into the genome of *S. rubber*[31]. Similarly, our comparative analyses indicated that the convergence of closely related functional genes from different sources is an important way through which new genomic content is acquired in haloarchaea and that foreign replication origins are usually introduced as a component of this new content. We cannot be certain whether the new genomic content (mixture of new genes and foreign replication origins) is introduced with single or multiple transfer(s), as the mechanism is not well understood; however, our analyses strongly suggested that the novel replication origins may be important for the acquisition of new genomic content and that the newly acquired genes from the surroundings may be favorable for the haloarchaeal cells to improve their ability to adapt to changeable environments.

### Recruitment of novel replication origins in the reconstruction of the extrachromosomal replicons

The haloarchaeal genomes in this study, except that of *Halorhabdus utahensis*, generally harbor extrachromosomal replicon(s), ranging in number from one in *H. mukohataei* and *H. walsbyi* to eight in *H. marismortui* (Table 1). In addition, *orc/cdc6* genes were found on most of the extrachromosomal elements (Table 1), suggesting that the *orc/cdc6*-associated replication origins are responsible for replication initiation on most of these replicons. Therefore, an in-depth analysis could further elucidate the evolution of these replication origins.

**Figure 5:**
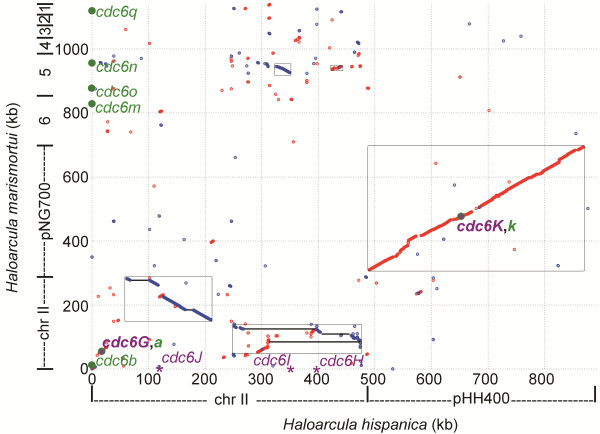
**Comparative genomic analysis of the extrachromosomal replicons of *H. hispanica *and *H. marismortui.*** The *orc/cdc6* genes (those from *H. hispanica* and *H. marismortui* are highlighted with a purple asterisk and a dark green round dot, respectively) that are associated with candidate replication origins are indicated, and the shared origins associated with *cdc6G/cdc6a, cdc6K/cdc6k* of the two strains are highlighted in bold. The homologous regions are boxed, and the lines in the box represent the regions that are continuous in *H. marismortui*.

Compared to *H. hispanica*, the *H. marismortui* genome contains a greater number of extrachromosomal replicons, with eight (minichromosome II and 7 megaplasmids, pNG100 to pNG700), while *H. hispanica* contains only two (minichromosome II and megaplasmid pHH400). Among these minireplicons, only megaplasmids pHH400 and pNG700 are collinear (Figure 5), suggesting that they may have been present in a common ancestor of the two *Haloarcula* species. The lengths of the minichromosomes of *H. marismortui* and *H. hispanica* are 288 kb and 488 kb, respectively. They share homology over approximately 100 kb, with a few inversions and gaps (Figure 5), indicating that this region was likely rearranged in the two *Haloarcula* species and thus that the two minichromosomes are only distantly related. In addition, the megaplasmids from pNG100 to pNG600 are unique to *H. marismortui*. However, pairs of orthologous to the minichromosome of *H. hispanica* are observed, especially in pNG500, with orthologs as large as 30 kb (Figure 5). Together with the abundant ISH (insertion sequence from *Halobacteriaceae*) elements encoded in these replicons, our data imply that the extrachromosomal replicons were significantly rearranged after the divergence of the two species and that new DNA contents were acquired from surrounding organisms. These results are also reminiscent of previous reports on the evolution of the large dynamic replicons found in *Halobacterium* spp. [22,39].

To understand the different composition of the extrachromosomal elements in the two *Haloarcula* species, the *orc/cdc6*-associated replication origins in these minireplicons were also examined. In *H. hispanica*, four predicted *orc/cdc6*-associated replication origins are distributed in the minichromosome, and one is present in the megaplasmid pHH400. The two origins (*oriC6cdc6I* and *oriC7cdc6J*) in the minichromosome and the one (*oriP-cdc6K*) in pHH400 were confirmed by ARS activity (Figure 1 and 5). In *H. marismortui*, the predicted *orc/cdc6*-associated replication origins are distributed among the extrachromosomal replicons as follows: two in the minichromosome, one in pNG700, one in pNG600, two in pNG500 and one in pNG100 (Figure 5). No *orc/cdc6* genes are encoded by either pNG400 or pNG200, and no candidate replication origin was identified adjacent to the *orc/cdc6* gene in pNG300, indicating that other types of replication origins are involved in the initiation of replication in these replicons. This concept is reinforced by the identification of *rep* genes in these replicons (Table 1) [40]. Among these replication origins, only two are shared by the two *Haloarcula* species, *oriP-cdc6K* in pHH400 and the origin (proximal to *cdc6k*) in pNG700, as well as the origin proximal to *cdc6G* and *cdc6a* in the minichromosomes of *H. hispanica* and *H. marismortui*, respectively (Figure 5). In contrast to the high conservation found in the megaplasmids pHH400 and pNG700, the region around *cdc6G* and *cdc6a* shows no collinearity (Figure 5), strongly suggesting that this origin might not have been present in their ancestor and instead was employed by *H. hispanica* and *H. marismortui* after their divergence. Surprisingly, a specific origin (*oriC7-cdc6J*) in the minichromosome of *H. hispanica*, which proved functional (Figure 1), was located in the region with high orthology to *H. marismortui* (Figure 5). This observation suggested that this replication origin was recruited into this region in *H. hispanica* or was lost in *H. marismortui* during rearrangement of minichromosomes in the two *Haloarcula* species. Similarly, the specific origins in pNG600, pNG500 and pNG100 and the *rep*-associated origins in pNG400, pNG300 and pNG200 were all likely recruited to accomplish the construction of these replicons in *H. marismortui*.

### Multiple evolutionary mechanisms account for multiple *orc/cdc6*-associated origins in haloarchaea

Our above analysis clearly indicated that the replication origins in haloarchaea are quite diverse and that different haloarchaea can share a few different origins. Although we cannot exclude the possibility that origin loss contributes to mosaic replication origins in haloarchaea, it is unlikely that all of the origins currently shared by different haloarchaea were present in the ancestor of each genus of *Halobacteriaceae* as *oriC1*. Archaea species often harbor mobile elements within their genome, which are mobilized via integrases [41] or transposases encoded by insertion sequence (IS) elements [42]. Our comparative analyses of the genomic context of the replication origins in the two *Haloarcula* species demonstrated the presence of mobile elements near their specific origins (Figure 4). These indicators of translocation processes were further analyzed in the genomes proximal to the origins in other haloarchaea. Forty-two of 102 potential replication origins have integrases or transposases nearby (Table 2 and Additional file 3), which might contribute to accelerate the translocation of these origins. In haloarchaeal chromosomes, the ratios of later-acquired origins are comparatively low, with a maximum of 50% for *H. marismortui*, *H. utahensis* and *H. walsbyi* and none in *H. borinquense*, *H. jeotgali* B3, *H. mukohataei*, *H. xanaduensis* and *N. pharaonis* (Table 2). By comparison, these later-acquired replication origins are widespread in extrachromosomal elements. For example, they account for 80% (4 of 5), 83% (5 of 6) and 87.5% (7 of 8) of the replication origins in the extrachromosomal elements of *H. salinarum* R1, *H. volcanii* DS2 and *H. lacusprofundi*, respectively (Table 2). These observations suggest that a portion of the replication origins in haloarchaea, especially those in extrachromosomal elements, were introduced through recent translocation processes.

**Table 2:** Predicted later-acquired replication origins in the haloarchaeal genomes

**Organism**	**No. of predicted replication origins**	No. of putative later-acquired replication origins *
*Halobacterium* sp. NRC-1	5 (2)	2 (1)
*H. borinquense*	7 (5)	2 (2)
*H. hispanica*	7 (5)	2 (2)
*H. jeotgali* B3	7 (5)	1 (1)
*H. lacusprofundi*	11 (8)	8 (7)
*H. marismortui*	11 (7)	6 (4)
*H. mukohataei*	2 (1)	-
*H. salinarum* R1	8 (5)	5 (4)
*H. turkmenica*	12 (5)	3 (2)
*H. utahensis*	2	1
*H. volcanii* DS2	10 (6)	6 (5)
*H. walsbyi*	2	1
*H. xanaduensis*	7 (2)	-
*N. magadii*	7 (2)	3 (1)
*N. pharaonis*	3 (1)	1 (1)

* Origins with indicators of translocation processes, integrases or transposases, etc., are predicted to be later-acquired. The number in parentheses is the number of replication origins in the extrachromosomal elements.

**Figure 6:**
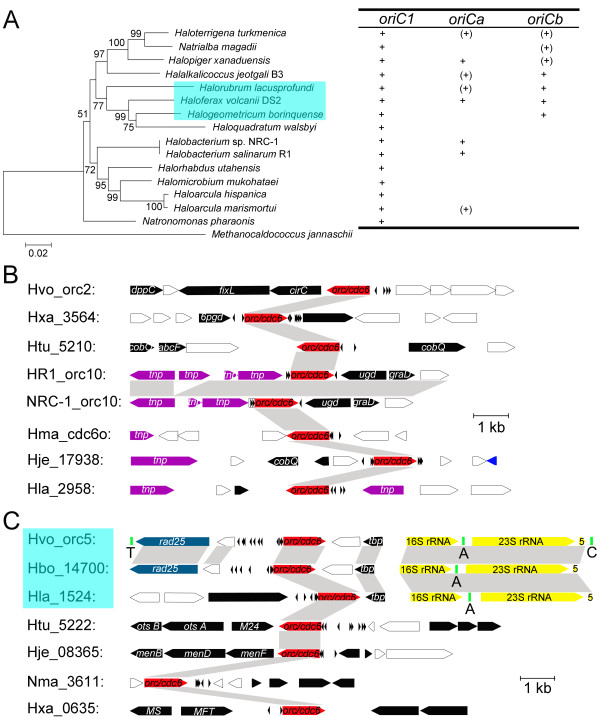
**Genome context analyses of the origins in the *oriCa *and *oriCb *families. A.** Phylogenetic tree based on the 16S rRNA genes (*Methanocaldococcus jannaschii* was added as an outgroup) and the distribution of the *oriCa* and *oriCb* families in the haloarchaeal genomes. + indicates the presence of this family of origins in the chromosome, and (+) indicates its presence in an extrachromosomal element. **B** and **C.** The regions around *oriCa* (B) and *oriCb* (C). Shaded regions denote similarity greater than 70% by BLASTN analyses. The *orc/cdc6* genes are highlighted in red; genes annotated with transposase (tnp) are highlighted in purple; rRNAs, including 16S rRNA, 23S rRNA and 5S rRNA, are highlighted in yellow; tRNAs are highlighted in green, with T, C and A representing the Thr-tRNA, Cys-tRNA and Ala-tRNA genes, respectively. *oriCb* origins of replication in *H. volcanii*, *H. borinquense* and *H. lacusprofundi* are indicated by teal rectangles.

Contrary to the complete conservation of the replication origin *oriC1*[10,37], the other origins are distributed almost randomly among haloarchaea, within both the chromosome and extrachromosomal elements (Figure 6A). To better understand the evolutionary history of these replication origins in haloarchaea, two distinct origin families (*oriCa* and *oriCb*, Figure 2), with the top two members excluding *oriC1* in this study, were selected for further comparative analyses. Interestingly, while the genes around *oriC1* are highly syntenic [10,37], the genomic context around *oriCa* reveals no similarity among the different haloarchaeal genomes (Figure 6B and Additional file 7). These observations indicated that the origins belonging to this family had different evolutionary processes from those in the *oriC1* family. Furthermore, transposases were observed near the origin in five out of eight genomes *Halobacterium* species (*HR1_orc10* and *NRC-1_orc10*), *H. marismortui* (*Hma_cdc6o*), *H. jeotgali* (*Hje_17938*), and *H. lacusprofundi* (*Hla_2958*)] (Figure 6B). These results suggested that these replication origins were likely mobilized via transposases, implying that association with transposases might result in an acceleration of translocation rates of *oriCa* among haloarchaea. Additionally, this acceleration may account for the random distribution of this origin family among different haloarchaea.

When focusing on the origins of the *oriCb* family, two different types were observed. One type consists of origins proximal to *Hvo_orc5* in *H. volcanii*, *Hbo_14700* in *H. borinquense* and *Hla_1524* in *H. lacusprofundi*. Some homologs were detected adjacent to this type of origin in all of the three genomes, showing that this type of origin directly links to a syntenic rRNA region containing the 16S rRNA, Ala-tRNA, 23S rRNA and 5S rRNA genes (Figure 6C and Additional file 7). In conjunction with the clustering in the 16S rRNA tree, this finding strongly suggests that these three haloarchaea shared their closest ancestor and that *oriCb* might be present in their ancestor. Notably, the two functional replication origins in the chromosome of *H. volcanii* were observed close to the two rRNA operons [9]. This closeness might benefit the preservation of origins over evolutionary time. Another type of origin in the *oriCb* family, including the origins proximal to *Htu_5222* in *H. turkmenica*, *Hje_08365* in *H. jeotgali*, *Nma_3611* in *N. magadii* and *Hxa_0635* in *H. xanaduensis*, was observed; this origin showed no similarity with respect to the order of the genes flanking the origin in different genomes (Figure 6C and Additional file 7). This finding implies a totally distinct evolutionary process. As three out of the four members of this type of origin were identified in extrachromosomal elements, it is plausible to propose that these origins were recruited for the construction of novel extrachromosomal replicons independently. Gene pools in environments were proposed to explain the adaption of prokaryotes under changeable environments [31,43]. Similarly, the diversity of replication origins can be thought of as a pool of origins in environments that can be recruited for the construction of novel replicons. This hypothesis sheds light on not only the random distribution of conserved origins in different haloarchaea but also the presence of extremely variable extrachromosomal replicons in haloarchaea.

## Conclusion

In this study, *orc/cdc6*-associated replication origins were predicted in 15 sequenced haloarchaeal genomes through Orc/Cdc6 protein analyses and adjacent ORB searching. Multiple replication origins were found in all of the analyzed genomes, and nearly two-thirds of the *orc/cdc6* genes were found to be associated with the predicted replication origins. We also experimentally investigated the predicted replication origins in *H. hispanica* and demonstrated that 5 out of 7 predicted origins possess ARS activity and that the remaining 2 putative replication origins appear to be dormant in experimental conditions. In conjunction with ORB comparisons and phylogenetic analysis of the Orc/Cdc6 homologs, various families of these predicted replication origins were revealed in haloarchaea. The diversity of multiple replication origins in haloarchaea was mainly driven by the diversity of Orc/Cdc6 proteins that specifically associate with distinct ORB elements. Interestingly, origins within the same family may have different functions among the various haloarchaea, e.g., although belonging to the *oriCa* family, the active origin in *Halobacterium* sp. NRC-1 (proximal to *orc10*) [10] was not proven functional in *H. volcanii* (proximal to *orc2*) [9]. These observations suggested differential origin utilization under different replicative conditions and demonstrated the advantage of our bioinformatic approaches in the identification of dormant or weak replication origins in haloarchaea.

Phylogenetic analysis of Orc/Cdc6 proteins suggested that multiple replication origins in haloarchaeal genomes can be categorized into at least two types: *oriC1*, which is present in an ancestor of archaea, and the other origins, which are likely specific in haloarchaea. We also revealed that transposases or integrases flank more than 40% of predicted replication origins; this flanking is indicative of the translocation of a portion of the replication origins among haloarchaea. In conjunction with comparative analyses of two families of replication origins (*oriCa* and *oriCb*), we suggested that different evolutionary mechanisms account for the diversity of replication origins in haloarchaea: preservation from ancestors (e.g., *oriC1* was maintained from the original ancestor of archaea, and one type of origin in *oriCb* was maintained from the closest ancestor of *H. volcanii*, *H. borinquense* and *H. lacusprofundi*), differential loss, and translocation among haloarchaea. In particular, a comparative genomic analysis of two *Haloarcula* species revealed that species-specific origins in the main chromosome were introduced along with new genes, whereas in the extrachromosomal replicons, the recruitment of novel replication origins usually accompanied the construction and/or rearrangement of minireplicons. The concept of an “origins pool” was proposed, and the introduction of novel origins in conjunction with the acquisition of new genomic content may be linked to the mechanisms involved in the adaptation of haloarchaeal cells to changeable environments. Taken together, our analyses of the diversity and evolution of the potential replication origins in haloarchaea may open avenues to understanding the significance of the multiple replication origins in the domain of Archaea.

## Methods

### Strains, plasmids and culturing

*Escherichia coli* were grown in Luria-Bertani medium at 37 °C, and 100 μg/mL of ampicillin was added when required. *H. hispanica* was cultivated at 37 °C in nutrient-rich medium AS-168 (per liter: 5.0 g Bacto Casamino Acids, 5.0 g yeast extract, 1.0 g sodium glutamate, 3.0 g trisodium citrate, 200 g NaCl, 20 g MgSO_4_ · 7H_2_O, 2.0 g KCl, traces of FeSO_4_ · 4H_2_O and MnCl_2_ · 4H_2_O, pH 7.2), and 3 μg/ml of mevinolin was added when required [44]. Plasmid pBI101 [32,33] was used for the investigation of the autonomous replication ability of the predicted origins. These plasmids were normally constructed in *E. coli* and then introduced into *H. hispanica* by a polyethylene glycol-mediated transformation method [45,46].

### Autonomous replication ability assay

Each PCR fragment, including the intergenic sequences and *orc/cdc6*, was amplified (see in Additional file 4 for primers) from wild type *H. hispanica* genomic DNA and was cloned into the nonreplicating plasmid pBI101 [32,33]. After sequencing, the plasmids were then introduced into *H. hispanica* or the corresponding origin-deletion strains (unpublished data) using a polyethylene glycol-mediated transformation method [45,46], and the mevinolin-resistant transformants were selected on AS-168 plates with 3 μg/mL of mevinolin. Plasmid recovery in *H. hispanica* transformants indicated the autonomous replication ability of the corresponding origins, which was verified by Southern blot analysis [9,14]. Briefly, the transformant on the plate was transferred into 200 μL of double-distilled H_2_O and 100 μL of phenol-chloroform and vortexed briefly. The supernatant (crude DNA) was collected for Southern blot analysis.

### Identification of Orc/Cdc6 homologs in the haloarchaeal genomes

Fifteen haloarchaeal genomes were available through NCBI, including the *H. hispanica* genome sequenced by our laboratory [20]. When searching the Orc/Cdc6 homologs in these genomes, a BlastP search (BLOSUM62 matrix; 1 × 10^-6^ as an e-value cutoff) was performed against all haloarchaeal genomes ( http://www.ncbi.nlm.nih.gov/sutils/genom_table.cgi) using the Orc/Cdc6 sequences from *H. hispanica* as seeds [47]. To focus on origin-associated Orc/Cdc6 homologs, PSI-BLAST (BLOSUM62 matrix, -e = 0.005) was also performed [47] using the profile from multiple alignments of experimentally functional Orc/Cdc6 homologs as a query. The results are summarized in Additional file 3.

### Prediction of *orc/cdc6*-associated replication origins

The IRs flanking *orc/cdc6* genes were collected, and a motif (predicted ORB) search was performed using MEME software (motif size: 20–40; ZOOPS model) [48]. Consensus repeats in the IRs were confirmed using DNAMAN software (for windows, version 2.6) [49], and those harboring “G-string” [9] were considered as candidate ORB elements. The IRs were verified by hand, and only those contained inverted ORB repeats and were structurally similar to characterized archaeal replication origins were considered to be candidate *orc/cdc6*-associated replication origins. The results are summarized in Additional file 4. Logo representation of ORB elements was performed using the program WebLogo ( http://weblogo.berkeley.edu).

### Phylogenetic analysis

16S rRNAs were collected from the 15 haloarchaeal genomes to estimate the evolutionary distance between them. The 16S rRNA sequence nearest the haloarchaeal-conserved replication origin (*oriC1*) was selected when there was more than one rRNA operon in the genome. Multiple alignments of the 16S rRNA sequences were performed using Clustal [50] implemented in MEGA [51]. A phylogenetic tree was constructed using neighbor-joining method [52] and maximum composite likelihood model implemented in MEGA, and 1000 bootstrap replicates were carried out. The Orc/Cdc6 homologs that were predicted to be associated with replication origins were collected from each of the 15 haloarchaeal genomes. The Orc/Cdc6 proteins, experimentally proven functional in their ability to recognize replication origins in other archaea (*Pyrococcus abyssi*[4], *Sulfolobus solfataricus*[5,6], *Aeropyrum pernix*[7,8]), were also included in this phylogenetic analysis. Multiple alignments of Orc/Cdc6 homologs were generated using Clustal (substitution matrix = BLOSUM; gap-opening penalty =10; gap-extension penalty = 0.1), and the result was adjusted manually to remove columns with many gaps. For maximum likelihood (ML) phylogeny, we used PHYML v3.0 with an LG substitution model and 100 nonparametric bootstrap replicates [53]. The data used to build the trees were deposited in TreeBASE ( http://purl.org/phylo/treebase/phylows/study/TB2:S12601).

### Comparative genomics and gene analysis

Whole genome alignments were performed using mummer and mummerplot algorithms in MUMmer [54] with default parameters. The GC plot was drawn using DNAplotter (window size: 50000; step size: 1000) [55]. Genome context analysis of the regions flanking the *orc/cdc6*-associated replication origins was performed using the NCBI Genome Workbench and scrutinized manually. Gene analysis was carried out using BlastP against the NCBI non-redundant proteins database ( http://blast.ncbi.nlm.nih.gov/).

## Competing interests

The authors declare no competing interests.

## Authors’ contributions

ZW conducted the experiment, data analysis and drafted the manuscript. HL, JL and XL participated in the data collection and analysis. HX conceived of and coordinated the research and finalized the manuscript. All of the authors read and approved the final manuscript.

## Supplementary Material

Additional file 1 **Physical mapping of eight predicted replication origins in*H. hispanica*.** ORBs found in the IRs are indicated with numbered arrowheads, and the sequences are listed below. Click here for file

Additional file 2 **Screening of origin activity in*H. hispanica*.** A. Schematic of the ARS assay. Δ: Corresponding origin (or *cdc6* plus intergenic region)-deletion *H. hispanica* strains (unpublished data) were used for transformation to avoid plasmid integration. * For the two origins, *oriC1-cdc6A* and *oriP-cdc6K*, which cannot be knocked out from the chromosome and megaplasmid, respectively, the wide-type strains were used for transformation and Southern blot was performed to confirm ARS activity (Figure 1). B. ARS assay plates for eleven candidates. Colonies in plates of AS-168 (Mev) were observed after 7 days at 37 °C, and the minus signs (−) represent no visible colonies (no ARS activity). Click here for file

Additional file 3 **Orc/Cdc6 homologues encoded in the haloarchaeal genomes.** The complete set of Orc/Cdc6 homologues identified in the 15 sequenced haloarchaeal genomes. Click here for file

Additional file 4 **Predicted*orc/cdc6*-associated replication origins in the haloarchaeal genomes.** Prediction of ORB-containing replication origins directly adjacent to *orc/cdc6* genes. The ORB elements are highlighted in red or blue colors. Click here for file

Additional file 5 **Alignments of ORB elements in origin families of*oriC1*,*oriCa*and *oriCb*.** A, B and C respectively represent ORB elements found at origins belonging to origin families of *oriC1*, *oriCa* and *oriCb*, and conserved sequences are highlighted with shaded rectangles. Click here for file

Additional file 6 **List of genes in the divergent regions between H. hispanica and H. marismortui. Blast analysis of the genes in the divergent regions between H. hispanica and H. marismortui. **The genes whose closest relative is outside haloarchaea are highlighted in different colors. Click here for file

Additional file 7 **Sequence similarity of regions around the*oriCa*and*oriCb*origins of replication in different haloarchaeal genomes.** BLASTN analysis of the regions around the *oriCa* (A) and *oriCb* (B) origins of replication in different haloarchaeal genomes, and gray shading represents sequence similarity greater than 70%. Click here for file
