# Supplementary figures and images for: Diversity and evolution of multiple orc/cdc6-adjacent replication origins in haloarchaea
Source: BMC Genomics. 2012 Sep 14;13:478. doi: 10.1186/1471-2164-13-478 (PMC3528665; doi:10.1186/1471-2164-13-478)

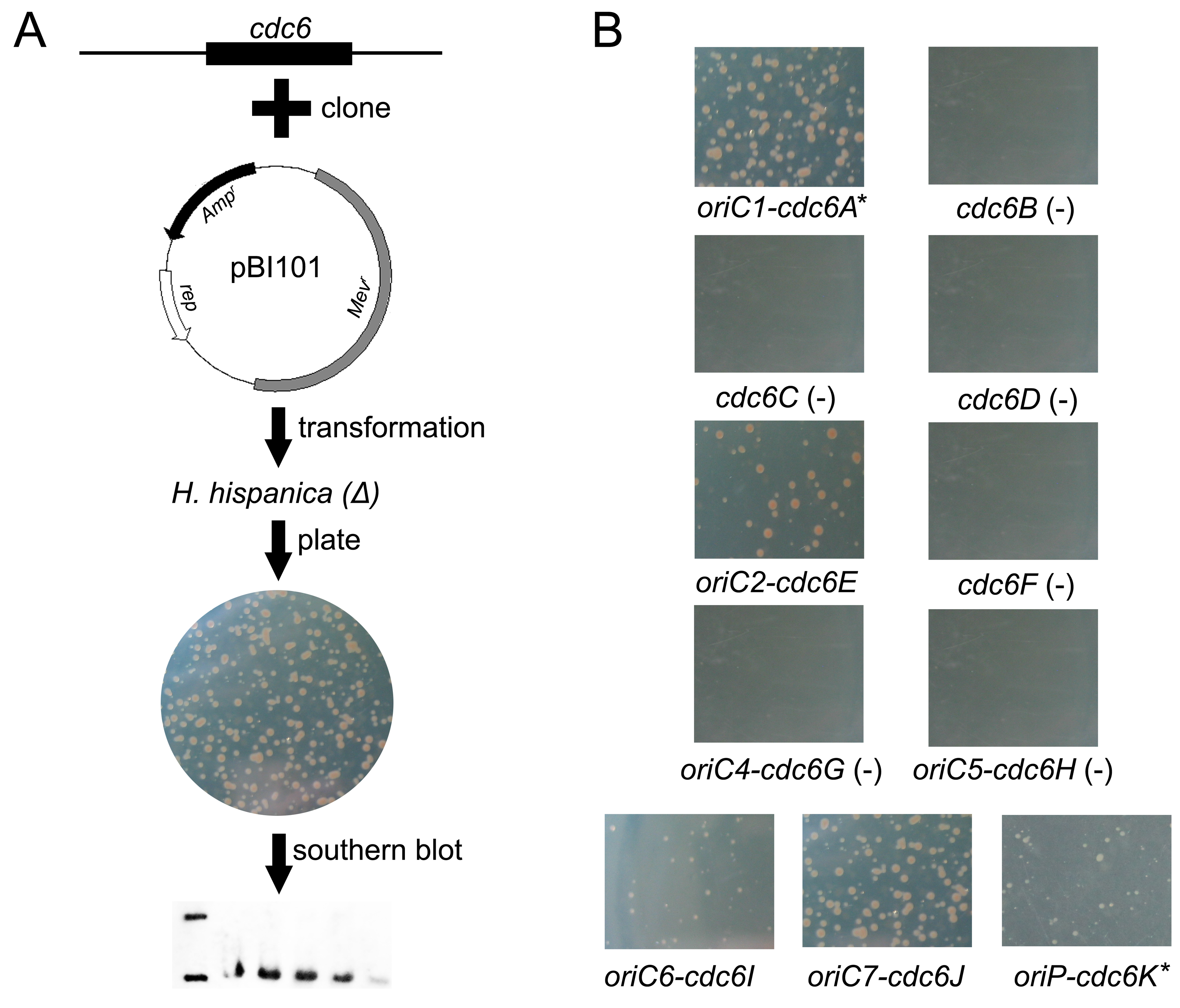

Supplement: Additional file 2 — Screening of origin activity inH. hispanica. A. Schematic of the ARS assay. Δ: Corresponding origin (or cdc6 plus intergenic region)-deletion H. hispanica strains (unpublished data) were used for transformation to avoid plasmid integration. * For the two origins, oriC1-cdc6A and oriP-cdc6K, which cannot be knocked out from the chromosome and megaplasmid, respectively, the wide-type strains were used for transformation and Southern blot was performed to confirm ARS activity (Figure 1). B. ARS assay plates for eleven candidates. Colonies in plates of AS-168 (Mev) were observed after 7 days at 37 °C, and the minus signs (−) represent no visible colonies (no ARS activity). [file 1471-2164-13-478-S2.tiff]

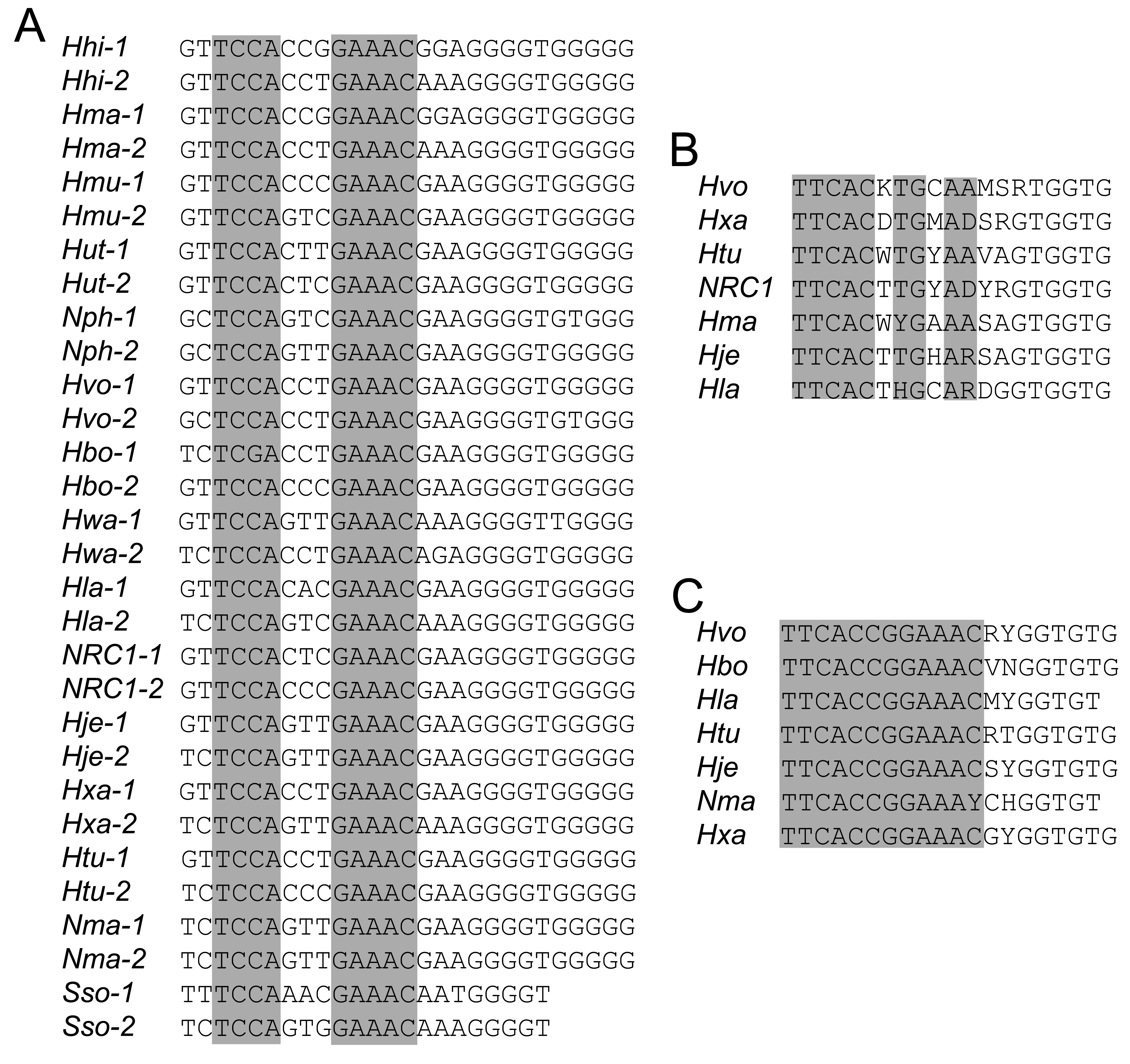

Supplement: Additional file 5 — Alignments of ORB elements in origin families oforiC1,oriCaand oriCb. A, B and C respectively represent ORB elements found at origins belonging to origin families of oriC1, oriCa and oriCb, and conserved sequences are highlighted with shaded rectangles. [file 1471-2164-13-478-S5.tiff]

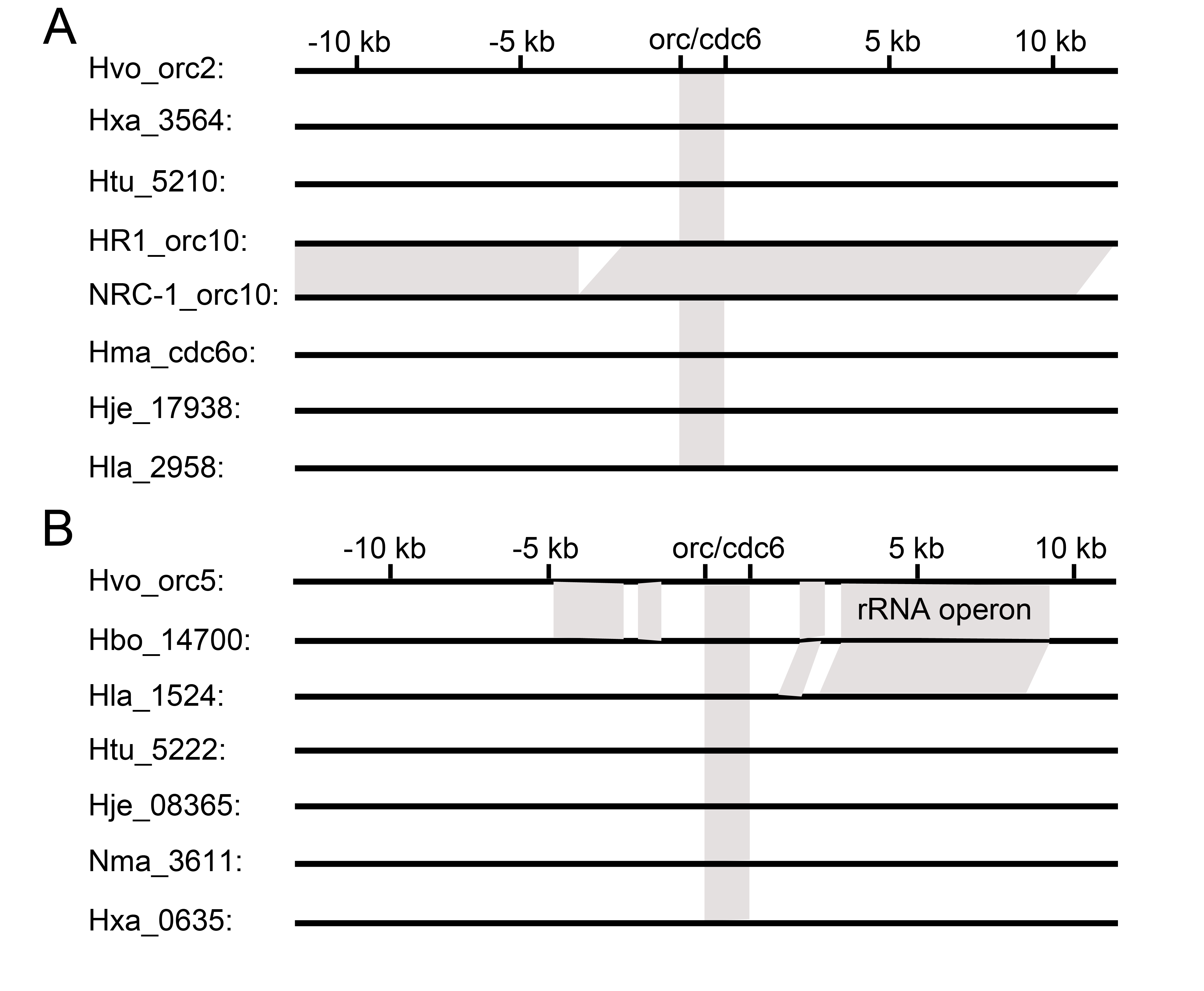

Supplement: Additional file 7 — Sequence similarity of regions around theoriCaandoriCborigins of replication in different haloarchaeal genomes. BLASTN analysis of the regions around the oriCa (A) and oriCb (B) origins of replication in different haloarchaeal genomes, and gray shading represents sequence similarity greater than 70%. [file 1471-2164-13-478-S7.tiff]
