# Supplementary material for: Diversity and evolution of multiple orc/cdc6-adjacent replication origins in haloarchaea
Source: BMC Genomics. 2012 Sep 14;13:478. doi: 10.1186/1471-2164-13-478 (PMC3528665; doi:10.1186/1471-2164-13-478)
Supplement: Additional file 3 — Orc/Cdc6 homologues encoded in the haloarchaeal genomes. The complete set of Orc/Cdc6 homologues identified in the 15 sequenced haloarchaeal genomes. [file 1471-2164-13-478-S3.doc]

**Additional file 3: Orc/Cdc6 homologues encoded in the haloarchaeal genomes analyzed in this study.**

* Orc/Cdc6 proteins whose number of amino acids is less than 300. ^1^ Locus in NCBI is used if the Orc/Cdc6 protein is assigned with name, or the locus_tag is replaced. ^2^ Transposases encoded by insertion sequence (IS)-elements and/or integrases are found near these Orc/Cdc6 proteins.

***Halalkalicoccus jeotgali* B3 (**CP002062-68**)**

|  | Accession number | Locus (Locus_tag) ^1^ | Replication origin associated | Transposase ^2^ |
| --- | --- | --- | --- | --- |
| Chromosome | | | | |
| 1 | [YP_003735318](http://www.ncbi.nlm.nih.gov/protein/300709504) | HacjB3_00665 | + | - |
| 2 | [YP_003735402](http://www.ncbi.nlm.nih.gov/protein/300709588) | Cdc6 | - | - |
| 3 | [YP_003735722](http://www.ncbi.nlm.nih.gov/protein/300709908) | Cdc6 | - | - |
| 4 | [YP_003736848](http://www.ncbi.nlm.nih.gov/protein/300711034) | HacjB3_08365 | + | - |
| Plasmid 1 | | | | |
| 5 | [YP_003738294](http://www.ncbi.nlm.nih.gov/protein/300712481) | HacjB3_15716 | + | + |
| 6 | [YP_003738531](http://www.ncbi.nlm.nih.gov/protein/300712718) | HacjB3_16951* |  |  |
| 7 | [YP_003738570](http://www.ncbi.nlm.nih.gov/protein/300712757) | HacjB3_17166 | + | - |
| Plasmid 2 | | | | |
| 8 | [YP_003738627](http://www.ncbi.nlm.nih.gov/protein/300712815) | HacjB3_17468 | + | - |
| 9 | [YP_003738665](http://www.ncbi.nlm.nih.gov/protein/300712853) | HacjB3_17658 | + | - |
| 10 | [YP_003738721](http://www.ncbi.nlm.nih.gov/protein/300712909) | HacjB3_17938 | + | - |

***Haloarcula hispanica* ATCC 33960** (CP002921-23)

|  | Accession number | Locus (Locus_tag) ^1^ | Replication origin associated | Transposase ^2^ |
| --- | --- | --- | --- | --- |
| Chromosome | | | | |
| 1 | [YP_004794626](http://www.ncbi.nlm.nih.gov/protein/344210306) | Cdc6A | + | - |
| 2 | [YP_004794759](http://www.ncbi.nlm.nih.gov/protein/344210439) | Cdc6B | - | - |
| 3 | [YP_004795860](http://www.ncbi.nlm.nih.gov/protein/344211540) | Cdc6C | - | - |
| 4 | [YP_004796254](http://www.ncbi.nlm.nih.gov/protein/344211934) | Cdc6D | + (deficient) | + |
| 5 | [YP_004796444](http://www.ncbi.nlm.nih.gov/protein/344212124) | Cdc6E | + | - |
| 6 | [YP_004797389](http://www.ncbi.nlm.nih.gov/protein/344213069) | Cdc6F | - | + |
| Mini-chromosome | | | | |
| 7 | [YP_004785663](http://www.ncbi.nlm.nih.gov/protein/344209486) | Cdc6G | + | - |
| 8 | [YP_004785785](http://www.ncbi.nlm.nih.gov/protein/344209608) | Cdc6H | + | + |
| 9 | [YP_004785833](http://www.ncbi.nlm.nih.gov/protein/344209656) | Cdc6I | + | - |
| 10 | [YP_004786038](http://www.ncbi.nlm.nih.gov/protein/344209861) | Cdc6J | + | + |
| Megaplasmid | | | | |
| 11 | [YP_004786119](http://www.ncbi.nlm.nih.gov/protein/344209943?report=genbank&log$=prottop&blast_rank=1&RID=ACE4JPTK01N) | Cdc6K | + |  |

***Haloarcula marismortui* ATCC 43049** ([AY596290](http://www.ncbi.nlm.nih.gov/nuccore/AY596290.1)-98)

|  | Accession number | Locus (Locus_tag) ^1^ | Replication origin associated | Transposase ^2^ |
| --- | --- | --- | --- | --- |
| Chromosome I | | | | |
| 1 | [YP_135272](http://www.ncbi.nlm.nih.gov/protein/55377422) | Cdc6j | - | + |
| 2 | [YP_135723](http://www.ncbi.nlm.nih.gov/protein/55377873) | Cdc6i | + | + |
| 3 | [YP_135910](http://www.ncbi.nlm.nih.gov/protein/55378060) | Cdc6h | + | - |
| 4 | [YP_136192](http://www.ncbi.nlm.nih.gov/protein/55378342) | Cdc6g | + | + |
| 5 | [YP_136193](http://www.ncbi.nlm.nih.gov/protein/55378343) | Cdc6f* |  |  |
| 6 | [YP_136906](http://www.ncbi.nlm.nih.gov/protein/55379056) | Cdc6e | - | + |
| 7 | [YP_137185](http://www.ncbi.nlm.nih.gov/protein/55379335) | Cdc6d | + | - |
| 8 | [YP_137318](http://www.ncbi.nlm.nih.gov/protein/55379468) | Cdc6 | - | - |
| Chromosome II | | | | |
| 9 | [YP_137925](http://www.ncbi.nlm.nih.gov/protein/55380076) | Cdc6b | + | + |
| 10 | [YP_137977](http://www.ncbi.nlm.nih.gov/protein/55380128) | Cdc6a | + | - |
| pNG700 | | | | |
| 11 | [YP_134598](http://www.ncbi.nlm.nih.gov/protein/55376747) | Cdc6k | + | - |
| pNG600 | | | | |
| 12 | [YP_134416](http://www.ncbi.nlm.nih.gov/protein/55376564) | Cdc6m | + | - |
| 13 | [YP_134425](http://www.ncbi.nlm.nih.gov/protein/55376573) | Cdc6l | - | - |
| pNG500 | | | | |
| 14 | [YP_134157](http://www.ncbi.nlm.nih.gov/protein/55376304) | Cdc6o | + | + |
| 15 | [YP_134241](http://www.ncbi.nlm.nih.gov/protein/55376388) | Cdc6n | + | + |
| pNG300 | | | | |
| 16 | [YP_134051](http://www.ncbi.nlm.nih.gov/protein/55376196) | Cdc6p | - |  |
| pNG100 | | | | |
| 17 | [YP_133987](http://www.ncbi.nlm.nih.gov/protein/55376130) | Cdc6q | + | + |

***Halobacterium salinarum* R1** (AM774415-19)

|  | Accession number | Locus (Locus_tag) ^1^ | Replication origin associated | Transposase ^2^ |
| --- | --- | --- | --- | --- |
| Chromosome | | | | |
| 1 | [YP_001688290](http://www.ncbi.nlm.nih.gov/protein/169235090) | OE1076F (orc10) | + | + |
| 2 | [YP_001689216](http://www.ncbi.nlm.nih.gov/protein/169236016) | Orc8 | + | - |
| 3 | [YP_001690034](http://www.ncbi.nlm.nih.gov/protein/169236834) | Cdc6 | - | - |
| 4 | [YP_001690150](http://www.ncbi.nlm.nih.gov/protein/169236950) | Orc7 | + | - |
| pHS1 | | | | |
| 5 | [YP_001690439](http://www.ncbi.nlm.nih.gov/protein/169237233) | OE7105F | - | + |
| 6 | [YP_001690446](http://www.ncbi.nlm.nih.gov/protein/169237240) | OE7115F | + | + |
| pHS2 | | | | |
| 7 | [YP_001690830](http://www.ncbi.nlm.nih.gov/protein/169237627) | Orc4 | + | + |
| 8 | [YP_001690940](http://www.ncbi.nlm.nih.gov/protein/169237737) | OE6225F | - | + |
| 9 | [YP_001690947](http://www.ncbi.nlm.nih.gov/protein/169237744) | OE6235F | + | + |
| 10 | [YP_001690980](http://www.ncbi.nlm.nih.gov/protein/169237777) | OE6288R | + | + |
| pHS3 | | | | |
| 11 | [YP_001690565](http://www.ncbi.nlm.nih.gov/protein/169237361) | Orc1 | - | + |
| 12 | [YP_001690594](http://www.ncbi.nlm.nih.gov/protein/169237390) | Orc3* | - | + |
| 13 | [YP_001690655](http://www.ncbi.nlm.nih.gov/protein/169237451) | Orc5 | + | - |

***Halobacterium* sp. NRC-1** (AE004437-38; AF016485)

|  | Accession number | Locus (Locus_tag) ^1^ | Replication origin associated | Transposase ^2^ |
| --- | --- | --- | --- | --- |
| Chromosome | | | | |
| 1 | [NP_279204](http://www.ncbi.nlm.nih.gov/protein/15789380) | Orc10 | + | + |
| 2 | [NP_280108](http://www.ncbi.nlm.nih.gov/protein/15790284) | Orc8 | + | - |
| 3 | [NP_280907](http://www.ncbi.nlm.nih.gov/protein/15791083) | Cdc6 | - | - |
| 4 | [NP_281018](http://www.ncbi.nlm.nih.gov/protein/15791194) | Orc7 | + | - |
| pNRC200 | | | | |
| 5 | [NP_395642](http://www.ncbi.nlm.nih.gov/protein/16120054) | Orc9 | - | - |
| 6 | [NP_395688](http://www.ncbi.nlm.nih.gov/protein/16120100) | Orc1 | - | - |
| 7 | [NP_395700](http://www.ncbi.nlm.nih.gov/protein/16120112) | Orc2* | - |  |
| 8 | [NP_395719](http://www.ncbi.nlm.nih.gov/protein/16120131) | Orc3* | - |  |
| 9 | [NP_395780](http://www.ncbi.nlm.nih.gov/protein/16120192) | Orc5 | + |  |
| 10 | [NP_395848.1](http://www.ncbi.nlm.nih.gov/protein/16120260) | Orc4 | + | + |
| pNRC100 | | | | |
| 11 | [NP_046015](http://www.ncbi.nlm.nih.gov/protein/10803617) | Orc9 | - | - |

***Haloferax volcanii* DS2** (CP001953-57)

|  | Accession number | Locus (Locus_tag) ^1^ | Replication origin associated | Transposase ^2^ |
| --- | --- | --- | --- | --- |
| Chromosome | | | | |
| 1 | [YP_003534076](http://www.ncbi.nlm.nih.gov/protein/292654179) | Orc1 | + | - |
| 2 | [YP_003534270](http://www.ncbi.nlm.nih.gov/protein/292654373) | Orc9 | - | - |
| 3 | [YP_003534696](http://www.ncbi.nlm.nih.gov/protein/292654799) | Orc2 | + | - |
| 4 | [YP_003535584](http://www.ncbi.nlm.nih.gov/protein/292655687) | Orc15 | - | - |
| 5 | [YP_003535768](http://www.ncbi.nlm.nih.gov/protein/292655871) | Orc5 | + | - |
| 6 | [YP_003536068](http://www.ncbi.nlm.nih.gov/protein/292656171) | Orc4 | + | + |
| 7 | [YP_003536157](http://www.ncbi.nlm.nih.gov/protein/292656260) | Orc16 | - | - |
| 8 | [YP_003536317](http://www.ncbi.nlm.nih.gov/protein/292656420) | Orc14 | - | + |
| 9 | [YP_003536318](http://www.ncbi.nlm.nih.gov/protein/292656421) | Orc11* | - | + |
| pHV4 | | | | |
| 10 | [YP_003533463](http://www.ncbi.nlm.nih.gov/protein/292653567) | Orc3 | + | + |
| 11 | [YP_003533525](http://www.ncbi.nlm.nih.gov/protein/292653629) | Orc13 | + | + |
| 12 | [YP_003533533](http://www.ncbi.nlm.nih.gov/protein/292653637) | Orc12 | - | + |
| 13 | [YP_003533715](http://www.ncbi.nlm.nih.gov/protein/292653817) | Orc7 | + | + |
| pHV3 | | | | |
| 14 | [YP_003533081](http://www.ncbi.nlm.nih.gov/protein/292493939) | Orc6 | + | - |
| pHV1 | | | | |
| 15 | [YP_003537021](http://www.ncbi.nlm.nih.gov/protein/292657125) | Orc10 | + | + |
| 16 | [YP_003537073](http://www.ncbi.nlm.nih.gov/protein/292657177) | Orc8 | + | + |

***Halogeometricum borinquense* DSM 11551** (CP001690-95)

|  | Accession number | Locus (Locus_tag) ^1^ | Replication origin associated | Transposase ^2^ |
| --- | --- | --- | --- | --- |
| Chromosome | | | | |
| 1 | [YP_004035096](http://www.ncbi.nlm.nih.gov/protein/313124832) | Hbor_00440 | - | - |
| 2 | [YP_004035261](http://www.ncbi.nlm.nih.gov/protein/313124997) | Hbor_02110 | + | - |
| 3 | [YP_004035472](http://www.ncbi.nlm.nih.gov/protein/313125208) | Hbor_04290 | - | - |
| 4 | [YP_004036490](http://www.ncbi.nlm.nih.gov/protein/313126220) | Hbor_14700 | + |  |
| 5 | [YP_004036705](http://www.ncbi.nlm.nih.gov/protein/313126435) | Hbor_16920 | - |  |
| pHBOR01 | | | | |
| 6 | [YP_004044301](http://www.ncbi.nlm.nih.gov/protein/313117318) | Hbor_31040 | + | - |
| pHBOR02 | | | | |
| 7 | [YP_004038009](http://www.ncbi.nlm.nih.gov/protein/313116885) | Hbor_33490 | - | - |
| 8 | [YP_004038104](http://www.ncbi.nlm.nih.gov/protein/313116980) | Hbor_34470 | + | + |
| pHBOR03 | | | | |
| 9 | [YP_004044608](http://www.ncbi.nlm.nih.gov/protein/313122681) | Hbor_39380 | + | - |
| 10 | [YP_004044619](http://www.ncbi.nlm.nih.gov/protein/313122692) | Hbor_39490 | - | - |
| 11 | [YP_004044622](http://www.ncbi.nlm.nih.gov/protein/313122695) | Hbor_39520 | + | - |
| pHBOR04 | | | | |
| 12 | [YP_004038327](http://www.ncbi.nlm.nih.gov/protein/313122440) | Hbor_36740 | + | + |

***Halomicrobium mukohataei* DSM 12286** (CP001688-89)

|  | Accession number | Locus (Locus_tag) ^1^ | Replication origin associated | Transposase ^2^ |
| --- | --- | --- | --- | --- |
| Chromosome | | | | |
| 1 | [YP_003176653](http://www.ncbi.nlm.nih.gov/protein/257386880) | Hmuk_0815 | + | - |
| 2 | [YP_003176838](http://www.ncbi.nlm.nih.gov/protein/257387065) | Hmuk_1003 | - | - |
| 3 | [YP_003177635](http://www.ncbi.nlm.nih.gov/protein/257387862) | Hmuk_1814 |  | - |
| pHmuk01 | | | | |
| 4 | [YP_003175707](http://www.ncbi.nlm.nih.gov/protein/257372933) | Hmuk_3249 | + | - |

***Halopiger xanaduensis* SH-6** (CP002839-42)

|  | Accession number | Locus (Locus_tag) ^1^ | Replication origin associated | Transposase ^2^ |
| --- | --- | --- | --- | --- |
| Chromosome | | | | |
| 1 | [YP_004595931](http://www.ncbi.nlm.nih.gov/protein/336252824) | Halxa_1419 | + | - |
| 2 | [YP_004596091](http://www.ncbi.nlm.nih.gov/protein/336252984) | Halxa_1580 | - | - |
| 3 | [YP_004596528](http://www.ncbi.nlm.nih.gov/protein/336253421) | Halxa_2024 | + | - |
| 4 | [YP_004596810](http://www.ncbi.nlm.nih.gov/protein/336253703) | Halxa_2306 | - | - |
| 5 | [YP_004597156](http://www.ncbi.nlm.nih.gov/protein/336254049) | Halxa_2660 | + | - |
| 6 | [YP_004597848](http://www.ncbi.nlm.nih.gov/protein/336254741) | Halxa_3357 | + | - |
| 7 | [YP_004598054](http://www.ncbi.nlm.nih.gov/protein/336254947) | Halxa_3564 | + | - |
| 8 | [YP_004598664](http://www.ncbi.nlm.nih.gov/protein/336255557) | Halxa_4182 | - | - |
| 9 | [YP_004598738](http://www.ncbi.nlm.nih.gov/protein/336255631) | Halxa_4257 | - | + |
| pHALXA01 | | | | |
| 10 | [YP_004586024](http://www.ncbi.nlm.nih.gov/protein/336252056) | Halxa_0635 | + | - |
| pHALXA02 | | | | |
| 11 | [YP_004598864](http://www.ncbi.nlm.nih.gov/protein/336251633) | Halxa_0082 | + | - |

***Haloquadratum walsbyi* DSM 16790** (AM180088-89)

|  | Accession number | Locus (Locus_tag) ^1^ | Replication origin associated | Transposase ^2^ |
| --- | --- | --- | --- | --- |
| Chromosome | | | | |
| 1 | [YP_656788](http://www.ncbi.nlm.nih.gov/protein/110666977) | Cdc6_1 | + | - |
| 2 | [YP_657497](http://www.ncbi.nlm.nih.gov/protein/110667686) | Cdc6_ | - | - |
| 3 | [YP_658656](http://www.ncbi.nlm.nih.gov/protein/110668845) | Cdc6_* |  |  |
| 4 | [YP_658657](http://www.ncbi.nlm.nih.gov/protein/110668846) | HQ2953A* |  |  |
| 5 | [YP_658659](http://www.ncbi.nlm.nih.gov/protein/110668848) | HQ2955A* |  |  |
| 6 | [YP_658663](http://www.ncbi.nlm.nih.gov/protein/110668852) | HQ2959A (Cdc6_) | + | + |
| 8 | [YP_658960](http://www.ncbi.nlm.nih.gov/protein/110669149) | Cdc6_* |  |  |
| 9 | [YP_658961](http://www.ncbi.nlm.nih.gov/protein/110669150) | Cdc6_ | - | + |
| 10 | [YP_659341](http://www.ncbi.nlm.nih.gov/protein/110669530) | Cdc6 | - | - |

***Halorhabdus utahensis* DSM 12940** (CP001687)

|  | Accession number | Locus (Locus_tag) ^1^ | Replication origin associated | Transposase ^3^ |
| --- | --- | --- | --- | --- |
| Chromosome | | | | |
| 1 | [YP_003129971](http://www.ncbi.nlm.nih.gov/protein/257052138) | Cdc6 | - | - |
| 2 | [YP_003129991](http://www.ncbi.nlm.nih.gov/protein/257052158) | Huta_1077 | + | + |
| 3 | [YP_003130520](http://www.ncbi.nlm.nih.gov/protein/257052687) | Huta_1613 | + | - |
| 4 | [YP_003130630](http://www.ncbi.nlm.nih.gov/protein/257052797) | Huta_1725 | - | - |
| 5 | [YP_003130789](http://www.ncbi.nlm.nih.gov/protein/257052956) | Huta_1887 | - | - |

***Halorubrum lacusprofundi* ATCC 49239** (CP001365-67)

|  | Accession number | Locus (Locus_tag) ^1^ | Replication origin associated | Transposase ^2^ |
| --- | --- | --- | --- | --- |
| Chromosome I | | | | |
| 1 | [YP_002564677](http://www.ncbi.nlm.nih.gov/protein/222478440) | Hlac_0001 | + | - |
| 2 | [YP_002565743](http://www.ncbi.nlm.nih.gov/protein/222479506) | Hlac_1078 | + | + |
| 3 | [YP_002565750](http://www.ncbi.nlm.nih.gov/protein/222479513) | Hlac_1085 | - | + |
| 4 | [YP_002566182](http://www.ncbi.nlm.nih.gov/protein/222479945) | Hlac_1524 | + | - |
| 5 | [YP_002567272](http://www.ncbi.nlm.nih.gov/protein/222481035) | Cdc6 | - | - |
| Chromosome II | | | | |
| 6 | [YP_002564204](http://www.ncbi.nlm.nih.gov/protein/222475683) | Hlac_2747 | + | + |
| 7 | [YP_002564283](http://www.ncbi.nlm.nih.gov/protein/222475762) | Hlac_2833 | + | + |
| 8 | [YP_002564394](http://www.ncbi.nlm.nih.gov/protein/222475873) | Hlac_2958 | + | + |
| 9 | [YP_002564429](http://www.ncbi.nlm.nih.gov/protein/222475908) | Hlac_2997 | + | - |
| 10 | [YP_002564636](http://www.ncbi.nlm.nih.gov/protein/222476115) | Hlac_3217 | - | + |
| pHLAC01 | | | | |
| 11 | [YP_002567439](http://www.ncbi.nlm.nih.gov/protein/222481203) | Hlac_3320 | - | + |
| 12 | [YP_002567484](http://www.ncbi.nlm.nih.gov/protein/222481248) | Hlac_3367 | + | + |
| 13 | [YP_002567617](http://www.ncbi.nlm.nih.gov/protein/222481381) | Hlac_3512 | + | + |
| 14 | [YP_002567643](http://www.ncbi.nlm.nih.gov/protein/222481407) | Hlac_3539 | + | + |
| 15 | [YP_002567740](http://www.ncbi.nlm.nih.gov/protein/222481504) | Hlac_3641 | + | + |

***Haloterrigena turkmenica* DSM 5511** (CP001860-66)

|  | Accession number | Locus (Locus_tag) ^1^ | Replication origin associated | Transposase ^2^ |
| --- | --- | --- | --- | --- |
| Chromosome | | | | |
| 1 | [YP_003401575](http://www.ncbi.nlm.nih.gov/protein/284163296) | Htur_0001 | + | - |
| 2 | [YP_003402081](http://www.ncbi.nlm.nih.gov/protein/284163802) | Htur_0510 | + | - |
| 3 | [YP_003402276](http://www.ncbi.nlm.nih.gov/protein/284163997) | Htur_0706 | - | - |
| 4 | [YP_003402720](http://www.ncbi.nlm.nih.gov/protein/284164441) | Htur_1155 | + | - |
| 5 | [YP_003402817](http://www.ncbi.nlm.nih.gov/protein/284164538) | Htur_1254 | - | - |
| 6 | [YP_003403401](http://www.ncbi.nlm.nih.gov/protein/284165122) | Htur_1843 | + | - |
| 7 | [YP_003403985](http://www.ncbi.nlm.nih.gov/protein/284165706) | Htur_2434 | + | - |
| 8 | [YP_003404109](http://www.ncbi.nlm.nih.gov/protein/284165830) | Htur_2559 | + | - |
| 9 | [YP_003404500](http://www.ncbi.nlm.nih.gov/protein/284166221) | Htur_2959 | - | - |
| 10 | [YP_003404690](http://www.ncbi.nlm.nih.gov/protein/284166411) | Htur_3152 | + | + |
| 11 | [YP_003405169](http://www.ncbi.nlm.nih.gov/protein/284166890) | Htur_3634 | - | - |
| pHTUR01 | | | | |
| 12 | [YP_003405444](http://www.ncbi.nlm.nih.gov/protein/284167166) | Htur_3916 | + | + |
| pHTUR02 | | | | |
| 13 | [YP_003406218](http://www.ncbi.nlm.nih.gov/protein/284172836) | Htur_4773 | + | + |
| pHTUR04 | | | | |
| 14 | [YP_003406514](http://www.ncbi.nlm.nih.gov/protein/284176237) | Htur_5210 | + | - |
| 15 | [YP_003406526](http://www.ncbi.nlm.nih.gov/protein/284176249) | Htur_5222 | + | - |
| 16 | [YP_003406548](http://www.ncbi.nlm.nih.gov/protein/284176271) | Htur_5244 | + | - |

***Natrialba magadii* ATCC 43099** (CP001932-35)

|  | Accession number | Locus (Locus_tag) ^1^ | Replication origin associated | Transposase ^2^ |
| --- | --- | --- | --- | --- |
| Chromosome | | | | |
| 1 | [YP_003478163](http://www.ncbi.nlm.nih.gov/protein/289579697) | Nmag_0001 | + | - |
| 2 | [YP_003478520](http://www.ncbi.nlm.nih.gov/protein/289580054) | Nmag_0368 | + | + |
| 3 | [YP_003479064](http://www.ncbi.nlm.nih.gov/protein/289580598) | Nmag_0918 | + | - |
| 4 | [YP_003479076](http://www.ncbi.nlm.nih.gov/protein/289580610) | Nmag_0930 | - | - |
| 5 | [YP_003480063](http://www.ncbi.nlm.nih.gov/protein/289581597) | Nmag_1930 | + | - |
| 6 | [YP_003480197](http://www.ncbi.nlm.nih.gov/protein/289581731) | Nmag_2065 | - | - |
| 7 | [YP_003480447](http://www.ncbi.nlm.nih.gov/protein/289581981) | Nmag_2323 | + | + |
| 8 | [YP_003480812](http://www.ncbi.nlm.nih.gov/protein/289582346) | Nmag_2694 | - | - |
| pNMAG01 | | | | |
| 9 | [YP_003481723](http://www.ncbi.nlm.nih.gov/protein/289583313) | Nmag_3611 | + | - |
| pNMAG02 | | | | |
| 10 | [YP_003482062](http://www.ncbi.nlm.nih.gov/protein/289937460) | Nmag_3963 | + | + |
| 11 | [YP_003482070](http://www.ncbi.nlm.nih.gov/protein/289937468) | Nmag_3972* |  |  |

***Natronomonas pharaonis* DSM 2160** (CR936257-59)

|  | Accession number | Locus (Locus_tag) ^1^ | Replication origin associated | Transposase ^2^ |
| --- | --- | --- | --- | --- |
| Chromosome | | | | |
| 1 | [YP_325954](http://www.ncbi.nlm.nih.gov/protein/76800946) | Cdc6 | - | - |
| 2 | [YP_325958](http://www.ncbi.nlm.nih.gov/protein/76800950) | Cdc6_1 | + | - |
| 3 | [YP_327196](http://www.ncbi.nlm.nih.gov/protein/76802188) | Cdc6_3 | + | - |
| 4 | [YP_327276](http://www.ncbi.nlm.nih.gov/protein/76802268) | Cdc6_4 | - | + |
| pL131 | | | | |
| 5 | [YP_327730](http://www.ncbi.nlm.nih.gov/protein/76803461) | Cdc6_5 | + | + |
