# Supplementary material for: Diversity and evolution of multiple orc/cdc6-adjacent replication origins in haloarchaea
Source: BMC Genomics. 2012 Sep 14;13:478. doi: 10.1186/1471-2164-13-478 (PMC3528665; doi:10.1186/1471-2164-13-478)
Supplement: Additional file 4 — Predictedorc/cdc6-associated replication origins in the haloarchaeal genomes. Prediction of ORB-containing replication origins directly adjacent to orc/cdc6 genes. The ORB elements are highlighted in red or blue colors. [file 1471-2164-13-478-S4.doc]

**Additional file 4: Predicted *orc/cdc6*-adjacent replication origins in the haloarchaeal genomes analyzed in this study.** Predicted ORB elements are highlighted in red color. ORB elements are found directly upstream of *orc/cdc6* genes in most of the putative origins; however, they are also found downstream and even around of *orc/cdc6* genes in a few origins, which are marked with # and *, respectively. Strikingly, two distinct ORB elements are found upstream each of two *orc/cdc6* genes, Hlac_3512 in *Halorubrum lacusprofundi* and Hwa_HQ2959A in *Haloquadratum walsbyi*, which are highlighted with different colors. Primers for determination of origin activity in *H. hispanica* are provided following the corresponding origins.

**1. *Halalkalicoccus jeotgali* B3** (CP002062-68)

**Chromosome (2)**

1. **HacjB3_00665**

137620-CCTGTCCTCCCGCGCCAGACGGCGCGTCCATGCGCGTAGACGAATAGTCAGGAGTACTTAAGCCTGCGTCAGACATACCTACAATAGATTATATAACGTCGTGCGGTGGCGATTCGGGAAGCGACTGCAGAAGTGACGGATCAGCGAAAGAAGCGTCCCAGCCGGTATCGACCTCACGAGAGGCCCATCACTCCCAACCCCCACCCCTTCGTTTCAACTGGAGACCGGTGGAGGAACGAGGCTGATCTCCGGATGGGAGTTAATTCCACTAGCTAATATTATCTATATAATATTATTTCTAGTTGAATTCATATCTGCTACTGCAGTACGGATATGGTTGTTCTGTGTTTGACTTAGCTGTTTCCCTTCTAGAACGACCACGGGAGGGGGGGGACTCTTGAGCGGTTCCAGTTGAAACGAAGGGGTGGGGGGGTTCGAACTCGTCTCGATAAACGTCTCTTCGTCGCGAGGGGAGGATTCGATCGCTAAAGACAGTTTTAATACCCTCGATTACCTTGAGTTCGCCTGCATCGACTGACGACCGACCAGCATCCGTATCCCGAGTGAACCGGTGTCTCGACTCCTCTCCGCTGTGATTCGGGGATGCGCGTTCTGGACTCCAGTTGAAACAAAGGGGTACGCCGGTCGCGGTTCACCAATCC-138281- **HacjB3_00665 (Plus strand, 138282-139775)**

1. **HacjB3_08365**

**HacjB3_08365 (Minus strand, 1542873-1544069)**-1544070-ACCCCACACCACGTTTCCGGTGAAATAGCG

CTTGTGTTTCGCCTCGGCGGACGGCCAAGCGACGCGAGCGCACACACACCGGGTTTCCGGTGAAACGGGAATTGAGGGCGAGTTACCCTCCAATCGAAGCCGGGGTGTGGGACGAACGTCGAACGGGGAACGGGGAACGGGGAACGGGGAACGGGGAACGATCCGGGCGCAGGTTCGGTCGGTAGTAGGCGAACGTTGAAGACGGCTCGAAGCGGCCCTGAGTGGCCATTCAGATGGAGAGTCGAGCCACGACGCGATCGAAGATCGCCTCCTTTCCTATTTTTGCGATCTACTTAATGAACTATACACTGGCTATAATACATATACTATTCGATACCGTCGAGCGTATCGCTCGGAAAGGCCGAATTCCGATGGGGTCCCTAACTGCCCTCGTTTCACCGGAAACCCGGTGTGTGTGTCGAGCCAGCAACCGATCGGACGGTGTCGGGACGCCGTTCTCTCGGGCGGGTTCGTAGTCCGGCGGATCGTTTCGCCCGAC-1544598

**Plasmid 1 (2)**

1. **HacjB3_15716**

**HacjB3_15716 (Minus strand,** **67895-69166)**-69167-GTTGGATGACACCAATAGTACGAAGGATAATAAA

CCCCTGTGTCCGGAGCGTCCGTCGTGTCCGCATATCAGATGTGGCTATATACCCCCTCCCCCCACCTTGTCCGCATAATCTCGCTACACATGATTCCCCCTTTCCAAGAACCGGCGATTGACCTAATGCTAGGAAAGATTAAAGTCTATAGCAGTTCAACCAGACCCGTACTAGAGTACGAGAAAATGTATCTCTAGTAAAGAATACCTCTAGAATAGCTTTCTGGGCTCTAATAAGGCTAAATTATGCGGACAAGGTGGGGGGTATAGACTAGTCGTGCCAATATGGTTCAGTGCTGACTTCTGTAGACTAGCTGGGAAATCCAGTCGATTCTTGCTTGCAGTTCCACCTGGTGTCCTCTCTTGTGTCTGAATTATGCGGACAGGGTGGGGAGGGGGCTAGAACAATTATCGAATCTCATCGAATCACGAGGAAGATCATCAAAGTAGGCGATGGGCCATATCTGTGGCCTTGTACAACAATCTTAACTGAATACGGACCGACTGTTGCACAAGGATCATGGCTCCGACTGCACCTTCG-69740

1. **HacjB3_17166**

**HacjB3_17166 (Minus strand, 395021-396247)**-396248-GTGGAGTGGACTCACACGACGTATTAATAAAG

CCACCGCTTCCTTAGCTTCCTAAGCGTCCGGAGCTTCCGAGTTTATAAGTGATAGTAGAACCACTGCTTCCGTAGTTCCAGATACTTATAAACACCCTCACCCCACTATTTCCGAAGCTCTGGTAAACAAAGATGGACCGCTCCTAAATCCTTCTATAGCAATAGAAATCTTTAAGTACGATATACGTTAACCATAACCGTAGTGGAGGTAGTGAAGGTTAGTCTAGTAGAACTCACACGTACTTTCTTCAGAATTGACATTTGATCACTGTCCTATAGCAGCATCATCCCTTTGGCCCTCTCTCTATTTCCGCGATAGCTTAGGAAACGGTGGAGTGAGGGTGTCCCCATATCCTCTCTATAGGAAGCTTAGGAAATGGTGGAGGGTTCTCACACTTCCTACTAGTTACTCCCCTGTTCAATCCCCTCTAAACGACGGTTGCTCCGGAAACAGTGGTGTGGCGAATAACAAAGCGTACGTCATTTTGAACCTCCTCTATCATCCTCCGAATCCTTTCGATCTGATTGAGACCGCCTTATCATGTATCAGTTTAACCAACATCGCTATCTGTAATCGCTTCTCGTTGGTTAACA-396873

**Plasmid 2 (3)**

1. **HacjB3_17468**

**HacjB3_17468 (Minus strand, 48078-49283)**-49284-CAATATGTGATATGTTGGGGATAGACAGTATAAA

ACCACCGCTTCACGTGTTTCATCTGTTTAGAGTATTTAAAACAGAGGGGGACCCGTGATTTCATGTGTTCTCCGGTAATGATTCGGGTGGAGATTGCGGAAGTCTCTATGACTAGCGGTAGTTTTAATACCTCATATAATGAAATGGTCCGTAGCGTCGCAGTGCTATATTAGTTAAAGTAGGAGTTAGTAACTAGGGTAGTTGTAGTCGGCGGTCTCTTGCAAGAATAGAACACATGAAATCTGGTGTGTCCCTCTTGGTTTCCCTCCCTCTTTGATGTTGACAGCATCTTCTCTTGACTGGGCCGTCTATTTGCGAAGAACACATGAAATCTGGTGTGTCCCTACCTGGATTGTTGCGCTATCCTCAATCTCGAAGTCGATGAGGAGAGCTTAGCTCCCGGTTCATTCCCATTGACGTGTTCGATGTCTCGTTGGACCATTCTGCCAAATGATTTGGTCCAGAAACTCGCGGTGACTATTGGTGTCGACTGGAAACGTCACTGTCTCGACGATCCATTAGGCAGCTACTGGTCGCTG-49856

1. **HacjB3_17658**

**HacjB3_17658 (Minus strand, 90802-92040)**-92041-GCTCACCTAATGGAAGAACGGGTATATAATACCT

CGGCTGCAATCGATACAAACGATTCATTCGTTTCCTACGTTCACTGGAGAGACACCACCGATTCAAACGTTATGGCTAAAGAGTGGGGGGAGACTTAGGAATGGTTCTAGTTTGAAGAAAGATTTAATACATAAGCGTGAAAACCAGAACCGTACTAGAGAGAGTATAGAAACGAATGAAATAGTGGTGGGTCTAGCAGGGGTCTAGTAATTGAACCACTTCTATCCCTGTCTAAGGGGCTCTTACTAGGTGATACATTTGAATCAGTGGTGTGCTTGAGGGGGATTCTACCCTCTATCTCATCTGGAAACGTTTGAATCGGTGGTGTCTCTCTCCTCTACAACCGATTATCTTCGTGCGATGAACTCTCACCACCTGTACGGTGAAACGCGTAGATCTCTCTATTGAACACAATCTCTATACTCATAACGTCCGATTACCCTCTGTG-92522

1. **HacjB3_17938**

146033-TAGCATTATCTTGTGGAGAGAGTGAGATTGCTCGTAGAGACTGGATCTTCTGAAGGCTATTCATTCTCGTGTTTTAGTACCTTGTGCAACATCGGTTCGACCAAAACGATGTTGCACAAGGTGGCTACGAGATGAAACTCAGACAGATAGTGAGAATCCAGAGAGACACCACTCTTGCAAGTGAAGCTGAGAAATAAGACGGGAGAGGGAGTCAAATGGGAGAATAACTAATCAGATAGATAACAGTTACTCCAACTATTCGAGAGAAGTGCTCAGAACACCAACAGAGAAGAGAAGAACTAGTTACATGTCAACGCTTTAGTTGTAATCATGGACTGTTCTTCTAGCTAGTCTCTCTAGTCTAGTGTTTCACTTGTAACAGTGGTGTGTGTCTATGACTTCACTTGAAACAGATGCAGGGGTGGTTTTATATAGAAAGCATGGGAAGAGTCTCAC-145578-**HacjB3_1793 (Plus strand, 145577-146803)**-146804-ACGGACCACACCACCCTTACAAGTGAGCTT

CACTTGCAGCAGTGGTGTGTGCGACCCTCTCTGTTTCACTTGTAAGAGTGGTGTGCTTCACTTGTAACCACGGTGTGTCCCTTGAGGACCTCAGCGTCAGCGACTCTCGTTAGATCGTTCATTCCTCTCTAGCTAACCTTGTACAACCGTATTAATAGAATAACAATCTTTGTTGCACAAGGTAGAATGCCCCCGACAGCACCTTCGTCTTTTCCGAACTACCTTGCCGAAGGATTTCCCAAGCAAGACGACAAGACACTTCAGGAAGCGCGTATATTGACGAGTTGCTTAAAGCCCGTAAGCACCGTTGAAAAGAGCCAGTCACTGAAGACGAATTACCCGAGAATGCTCAGGTTCTTAAAGACGAATCGAGTAGCGCAGTGTATCTCGAATATCGAACCTGCGGTGACGAGAGTTGATCGTGTATGAGCGGCGGTGCAAAACACGGCCCGTATAAGTACCGTGCCTACCGCGACGGTGATACCGTCCACCGGGACTACCTGGGGAAAGCAACCAAGACGGACACTGATACCAATGCTGATTAACACTGATCAGCCATGGCTCCCAGCCGAGATCGATCGTGTCTCGTGGTCGCAACCTTCTAGAGTGCTGAGTGGTAGTACTGAATGTA-147464

**2. *Haloarcula hispanica* ATCC 33960** (CP002921-23)

**Chromosome (3)**

- 1. **Cdc6A**

2994336-CCTATCCTCCCGCCGCCCGGAGTCGTCCGGTATGGCGGCTCTGCCCGTAAAAGATAGGCGAATTCACTTAAGCCTACGTCAGACATTAGTCCTTTTTCTGACATGGGGAGGCATAGCACAGATCAGCGTGTGCGCGTTTCCGCGGATGCAATCGGGACGGGAACTGTTCGGGTGGAGTCCGGTCATCCGAAACCCAGCGATTCGGATTAGGGGTGGACCATCCTAGCCCCCCACCCCTTTGTTTCAGGTGGAACGACAGACATCCCTGGGGAGGGGGTGGAGAATTACCGATCAAATCGGGAGCGGACCAGCTGTTCAAATCGGCGCGACTCGCCATACCGCTAGTTAGAACGGCCTAGACACTAGAAAACTGTATACTACCCTCTACTAGGGATTTGTTTTATCTAGTGCGGCTTTGGTTTATGAGGTAGTGTATTAAACCTTTCCCCTTACTAGAGGACATTTTCGTCACCCTCACCCCCTCCCCCCGGGCCTGTTTCCCGTTCCACCGGAAACGGAGGGGTGGGGGGGGTTGGCCCGTTACGGTCGCTTTGTCCTACAGGTCCCGTCCTGCCGTGGTACCACCACTCGTGTTCACACAGCGTACCAGCTCCCGGAACCACTCCATGGCTCCCCTTCCCCTCCACTCATACCCCTGCCGTGCCATGCCCGTCTAACCCAGTGAAACTGAAGCTATAGTCCTCTTTACGACAACGGAACCCATGCGGGAGCACTCATAAATATGCCAACATATATGTTTCATGGGTGGTGTACCGAAATATTTAATATCGACTCACGGTACGGTTCATGTGGTTCAACTGGACGCACCGGAGAGCGCTGATGCTCACTAACGACGGTACTGACGCACTCCTCGCTCCCTCTGTTCCGGTGTCTCCAACTCTCTCCACACGAAACGAAGGGGACTCTCTCGACT-2995271-**Cdc6A (Plus strand, 1-1575)**

POC1F: AAAACTGCAGAATCCCATCCTATCCTCC

POC1R: CCCAAGCTTTAGCCGTATCACTCGAAG

- 1. **Cdc6D***

1600447-CGGGGCTTACGGCCTTCACCCTCGGGGTCAAGCCCCGAGGCACTCGGCCTGCTCCGCCAGTAGACGCTGCGCCAGTGGTGAAATGAGGGTAAGTACTCCTATCTAGATATAGTCCATCGTGGACTGCATGCCTTCCCGCGCGTGACTTCAAGTAGCCTAGTGTGGGCAGTAGGCGATTACACTCGGTAGTGGGTGTGTGAAACTCCACTCAGACATATATAAATTTATGTGAAAAGCTACTAAAATGGTTTTAGACCGGTTTTACTAAACTTCACTCGTTGCTTGGTCGCTACATTTATCATACAGGGACGGATTTGATAGAGTAAATA-1600775-**Cdc6D (Plus strand, 1600776- 1601993)**-1601994-CGATGTTTCACTCGTTACGTGGTGTGTGGGTCGGC

TTCGATATTAAACCGCAGAGGGGAGCCAAAGCGCAGTTCCGTTTCCGCTAGAGTTCATG-1602088

POC3F: CATGCCATGGATGGGCGAGAAGCGGTC

POC3R: CATGCCATGGCATGAACTCTAGCGGAAACGG

- 1. **Cdc6E***

1800336-CGGAGAGGGCTCAGTCGATCACTGTCGATTGGTTGATTGCGTCGCAAACGACGTATTTTTCGCTGGAACACCACCCTACGAGTGTTCTGACAGCCCTATACAAACGATCCGACACCACCCTACAAGTGTTCTATCACCACCCTGCAAGTGTTTCCAGTACGACTACAACTGGACCAGAACAGTGGACAGGTGTTCACACCCCACCCTGCAAGTGTTCGCACGGGGGTATGCGTCGTGCGGGGGTGCGCGAGCGTCTGAGATTCCCGAAAACACCACCCTGCAAGTGTTTTTGAGACGTTGTTCGGAGTTCCGATGCGCCACTCTAACACCACCGTGCAAGTGTTTCCCGCACTGGGAGAGACAGACGAGAGCCCACGGGAACACCACCCTGCAAGTGTTCAGGAAAGCCCATTTTGAGCGGTTTTCCCGGCGAATCGGTGTTGACACCACCCTGCAAGTGTTTTCGAAACGGAATACCGGGAGGCTGTGGTGGAAACGGACGGACACACCGGTCTGCAAGTGTTCTACACACTGCAGTGGAGGGTAACTGCCACATAATCGCTCGGTAGCACTCCGGGTTCACGGTCTACTGTTGGCACACACACACCACCCTGCAACTGTTCTGGACTGTACAATAGAGGGACTGCCACTGAACACACACACACCACCGTGCAAGTGTTCTGCCATGTGAGTCGGTGGGGGGAGGGGAGCAAATACGCTGG-1801057-**Cdc6E (Minus strand, 1801058-1802386)**-1802387-CGTACGCCACCTTTTGAGTGACTCTATATAAAAC

CAGTGACCACCGTACAAGTGTTTCAAGTGTTTTCACGCCGAACAGTCACACACCACCCTGCAAGTGTTTCTTCGATCCCTCTGGAAAACAGCCACGAGCAGTCTGCTGCACCGACCCTTCATCTAGAACACTTGAGTCCTGCGATTCTCGACTGTCTCGTTCTGGACCGTATCTAGAACTAGTAGCACTATTATATATTTTATGGTAAAGGCAATAAAATCATATTCACCATCGAACGTCCACCTCCATAGAATTCAAGCTACAAGTGCTTCAGAGCACTCATATCTGCATATACGAATAGTCTTCTGATCATGTACTCTCAGTAGAATCGAACAATCTGCCATTTCTTTAAGTATGCAGAGGGAGCCTCCCCCTCCCCACCCCCACCCCTGGAAACACCGAGAACACTTGCAGGGTGGTGTGTGTGGGTCCCGCTTTTCCACCGACATTTCGGTATAAAAGTACTCAGCGTCGATACGGCATGAAACCCCTGCCATAGCGAACGGTTGGTCATATAGCGAGCTAGCCCGTCACCCGCGGCGCACGGCACCGCTCTTTACTTTCTGACAGCCCGTAGACACCGT-1803004

POC2F: CATGCCATGGCTACGTGAACTGCCAGAGG

POC2R: CGCGGATCCACGGTGTCTACGGGCTG

**Minichromosome (4)**

- 1. **Cdc6G**

488056-CGTGTGGAGCCGCCGGCTGCGAGGGCTGCACAAAACGAACATTGTGGGCAATAGCTCAATCGCCTACATGATGTATTAGAACAAACAAGATGCCTGAGTTCGGTTAGATAAACAGATCTCTCATGTGGTACAGGGTGTGTGACCTGAATAATTTCAGCTGCGATGTTATCTTTGATCTGTCTCTTTTCAGGAAAGTCTCCAGATGAGATTTCCAACGCAAACACGCTACGCAGGCGGCGTAGTTCAACTTTGATAGTGTCTTGCTGTATCGGCCGTAGAGAAGTGGGACCCCCCGGTTTTCACGAGAGTTAATATCGGCCAGTGAACCGGCATTTTGTTGCCCACCCCCCACATTTCACGAGAGTTTAAGAATAGCAGCTCCTAACTCATGAAATGTGGGGTGTAGTTGAGCAGAGCAAGTCATCAATTGTGTAGTAACTCAGAGAAAGCACGGAGTCTAGACAGTGAGTGGAGAGGGATTGATGGTCTCTCAGGGAATCAAATACAGAGCAGCGCGACCGTAGACTCCTATGGATATAACCTACTAGCGATGGATATTGTTTGTTGACGACGATAACTGACCATGAGAAGTTGAGGTCTCCTTTCTGCCTTGCTGAATAGAAGAGACAGGTAGATATCTCCCCCGCCCCCCATATTTCATGAGAGTTGCTATAGGGGAGGGAAGAAATAATGTCTGACGCTTCTTGCTGAGTTTCCACTAGAGAATTAAACTGAACCTAACGTATTACGGATGCTGTTTTGACCCACTTATATTAAATCTTCTTCTCATTCAGAGTTTCATTTTGCCCTCTCTTCCCCTCCTCTCCAACGATACTCTCGTGAAATCTGGGGGGCGGGGGTTG-488918-**Cdc6G (Plus strand, 1-1209)**

POC4F: CATGCCATGGGTGGATGAGAAATTGGC

POC4R: AAGCGGATCCTGGACATCGTGTGGAGCC

- 1. **Cdc6H**

**Cdc6H (Minus strand, 108513-109421)**-109422-ACCAACACAATCTCATGGACTTCATATTAATATTGTCC

ACCACCTTGCCGCTGTAAAACAGGTAAGATTGGTATGCTATATTTGAAAAGTCGTATATCAGTCGCTCAGTTCCCATCTATTGGTGATATCCTACTCCAGGATACTGTGTGAATTTAGAAGGTAGTAGTACTCCCACACCCCGTGTTGCCGCTGTAACAGTTGAGATGAGTTTCCAGATGAAAGCGGGCACAATAATGAGTGATAGCATACCTACACGCTATCTACACGCGCTTGAACAGATCCTGTGTAGAAGGGGTGAAAACCGGTATTTCACGCTACCGACGCTAAAGCGTCGTTATTTTAGTACGCTAAAGCGTGGTACGGTACCAAACAGCATAGATCATAATAAAACTGATGATGCCTGTCCTAATTTAGTGGCTACGCGGAGTTATCAACTTGATATGGTAACACGCTGTGGCTGCTTTGCCGATTCACTTACAGCGGCAATGAGGGGTGTACAAACATGACCTCGCATAAAAATAAGAGACAGGCAACTGTTCTCGATTGTTTACAGCGGCAACGAGGGGTGCGACTGCAGACTAGTCTAATACTAGCGTGTCATAGAGTACGTCACACACCCTCTCCTCCTTGCGATCACCGCGGATGATTTCGCCGTCTGGAAAGTCTTCCAATCTGGTGCCACACTCTGTAAATTACCCCTTATAACCCTGTTCATCGTATTTTGAGACGACCGCGTGAACCACGTTCTCAATCTTGACTGCGGCGTGGCCATTCAGAGTCGGAAG-110206

POC5F: CATGCCATGGTACAGAGCGGGTGAGCCT

POC5R: CGCGGATCCCCTTTGGTCCTGGTCGTT

- 1. **Cdc6I**

150036-GAGGTCGAACCCCCACACCCCGTGTGCGATATGAAATCGGGAGGAGAGTGGGAGGGGGTGGTCGAAGAGATAGACAACGAAACACCAGAAATCCGCGATTTCATGGAAAAGAATTCTCAAAGACAGCAATATTAAGCTGACTACATCTTATGGAAATCTTTATATAGCTACCCTGTCAACTATATCCGTACCGGAACTAGGGTCTGTCGAAATGGAATAGTATATCTCGTGACATAACAACTCTCAATTGTTCCTTTGCTTTTTCCTGGGTTGACGGTCTTTGCCGGTCCTGTGTCTTCTCGTGGGTTCTCCGGGCGAATACCAAGTTCCTCAGATGATTCATCTCCGTTCCCCCTCTCGCTAAATTCATATCGCACACGGGGTGTGGGGGTAGCTTAGTGACGAGATCCCATCTCACTCAGGATAGCCGAGAGATATTCTTGGTACTATCCGGCCCGAGACTACGTCTGATATTTCATCTTGCACACGGGGGGTGCCTCCGCCGAGTTCTTTTGACTCGCCTTGACATCAACAGCTGTTTGATGAATTATTCGGGATTTCATATCGCACACGGGGGGTGACTACGACCAATTGATTTCGCCCGCGGTTACCCAATCTCATCTACTACCCTCGCCTAAATGCACTCTACCTCTTGAATTCGGATGATTTAACCTCGCACACGGTATCACTATTTATTTATAGTTTCGCCCTAACTGCGCAAAC-150758-**Cdc6I (Plus strand, 150759-152105)**

POC6F: CATGCCATGGACGGAGTCGAGGTAGGG

POC6R: CGCGGATCCGTTCGGAGTGGGTTGTTG

- 1. **Cdc6J**

**Cdc6J (Minus strand, 383352-384539)**-384540-TTGTGTGTAGTTGCAACACGGAGTGTCTTAACCTTTGG

TTCCGCTGCTTCCGCTGTTCGGTTGGGTATATAAATGATTGACCCCGCCACTTCCGCTGTTAGATGAGAGGGGAACACCTGACTGACCACTCCCCCACCCCACCGCTTCCGCTGTTAGATGGGAAATGGGATAGCAATCCTCCTACTCCCCCACCCCACTACTTCCGCTGTTAGATGAGAAATGGGATAGCGACCCTCCTACTCCCCCACCCCACCGCTTCCGCTGTTAGGGCGGTAGCCGAGAAGTTCCCTGGTCGAGGAACGGGATCGAGTAACTGCGAGTGGTTAGACAGCGGGATTTCGCGGGATCATAGCTGTGGAGCGACACACCCACCCCACTGGTTCCGCTGTTAGGACCGAAAGTTGGTGGTGACCTATCCAGATCGCGGCCTGAATATCTCTAGAGCTAGTGAATAACTTCAAGTGCTTCGGCAAGAAACCAAAGCCGTATGACGTTATCACAAAATAGCAAAGCACCCCCTCCCATGGGGGTGGTGCCGTCTATTTATTCCCTAACAGCGGAACCAGTGGGGTGGGGGTGTGGACCCACTCTTATTACAGTTGCTGGGGCAGGCCATCCACAACTTCCAGTTGAGGCGGAGAGTCGACTGAGGCGTCTATCGACTCGACACAGCGGTGAACCATACGGCCCAAACTTACGCCGACAGATGATACCCGGAGTAGACTTTATTCATGAGCGAGAGTAAAACAACCGCTCGGTAAGCGTACGGATTGACTGAATCCAGAACTGTAATATTACTTGAAGCTTCATTGTCCATTATGCCCGACAAAAAACCACAGCCAAATAATCCATTAGCTCCGTATTATCCCTCAGAAACGCCGAGTTTGTCGGGGAATGGTCGAAAGTGGTTTGCCACACTTGTTCTCGGGTTCTGGCTATTTCTGGCGGCGATTGCGCTCGCTGTTGAGCTTTTCCTGTAATTTCGCCACAGTTGCGACTGAGAACCGCCGGCACGAATAGACACACCCGGCAGCGCCCAATATCCATTTTCAGCGTATTACCCGCGTCTCTTCACCTCACGGCCCCTCTCCAGTTTCGCTCGCACCAGACTGGGCTCCCGATCACTCACCGATAATCGCTGTGGAGCCGGCCTGCCCC-385697

POC7F: CGCGGATCCGTGAGTGATCGGGAGCC

POC7R: CGCGGATCCCATCGTAAGCAGCGGTT

**Megaplasmid (1)**

- 1. **Cdc6K**

404716-CAGCTAGCGTAGAAGGTATCGATGCACCACGACTGTGTTTGAACCGCTCGAATAGGAGGAAACAGCAGAACAAGATGGAAGGGAACAGCAGAACCCAGCGGGTGGATTGTTGGTTATCGCGATAGCTGGCAGACTGGGCTGTCGCTGAACGTGGGCAAGTAGTATTCAGTGATCGGTTGAGTACAGTAACTGTCGCGGCAGATGAGCAGGAGCGCTGCGACGACTAACGTGACCAATGCGGCGCGAACAGTGAATCCCGGACCAGAAGCAGAACCCGAATCGTTGAGCGGGCAGGATGAAACAGAGAACACCACAGTGTCTGCAGTAACACTTCGTTAGAGGTGTTCTAAACCCATATTACTGCGGACATTGTGGCGTCTGACGGGGAGGTCTGGTGCATAATCGTGAATAGACACCACGATGTCCGCTGTTCTATTGGGACAGTTGAGAAATCATGCGCCGATTACTGCAGACATTGTGGTGTTGCTTCGCCTGGGCCACACCACGATGTCCGCTGTTCTAGTGGGGGTGGACACGAGAACAGCGGACACGGCGGTGTGTGCTTGAACAATAGAAGGGCTATAAATTGGCCACAGAGAGTCTGAAGACCGTAAATTAGGCGAGGAATGGGAGAGAACACTCCCACCCCACTTTGTCCGCTGTTCTTCAAAAGAGAGGGAGGGGGGAAGGAAACAGCCTCATTTGATTCGAAGGGTAGTCAATTTAGAACAGCCTGGATACTATCTGAACTTCATCTAGATAGAATATAGACAGTTTAGATTTTACTTAGTCTCAACTAGATTGTGTCTAGATACTACCCAAATCTTATCTAGACTAGAGATAGCGTCAAGTATTGCTATAGCGTCTTTCTCTATAGCTATTAAACTGCACTACGGTCTAGCTCTCAGCATAGTATTATAAACTTTTCCTTAACGAATAGTTTTCTGAGTTACCCCCACCTACCGTTTTCTAGTTACTGCGGACATAGTGGGGTGGGAGGGTCCCGCCATTCTTTTATAAGCAATAGAACAGCGGACACTCCGGACACTGCGGACAGGGGTGGTTTTATGTATGTTCCTTCCAATACATCAGGCAACGACT-405816-**Cdc6K (Plus strand, 1-1245)**

POPF: CATGCCATGGTCTCGTCTGGGTTGGTAG

POPR: CGCGGATCCTAGCGTAGAAGGTATCGATGC

**3. *Haloarcula marismortui* ATCC 43049** ([AY596290](http://www.ncbi.nlm.nih.gov/nuccore/AY596290.1)-98)

**Chromosome (4)**

1. **Cdc6i**

936322-ACTCAGTGTTACTCTTTCCACTTATTAACACCTAACCGATAAGCCGTCAAGCGTCTCCGTGTACGAGTCAGCCCCTTGCATCCACCGGGCCAAGAGTGTACCTGTCACCGCCCGGTGATGAATGACGTCGTACTGCGTTCGAATCGTCCGTGCCCGGATTGTCGTATCGCTTCCTTCGTGAGCTCTCATATGGACTACGCGGCTGCTGCCGTGGGTGGCGCTTTGGGGTGGGTGTGCCGTTGATACAGGTGTGCTGGTCCCTGCCTGACTAGTGGAAAGCTTGTCCCAGACGCACCTGACACCGAGTGTACTGACTGGTTGTCATTGTTTGGTCGATAGATTCTCGTCTCAGACACACCCCGCACCGAGTGAAAACTCGGGACCTCCACGATATTTCTGATGAGCGGCAAGTAACCGGGTTTCGGGACGCAAAGCGGACAAAGCCGCGAAGTCGCGGGTTGAATCGAATATGCGACCCGACTGCCGTTTTACACTAGTCCGCGAGCGGCTCTCGCAGGCACACACCAGGTACCGAGTGTACTGACCGCTGATCGTTATCGGCACAAGAGTGCTCTGGTAGCAAATATCTCTATCGAATGGCTGGAACCAGATCAGATGGGAACATCCAGTACAGCCATTGGCCCCTGGGACACACACCTGCTTCCGAGTTAGTTCAGTTCTCAAATTCATGTGTTAGAGGGAAATCAAACGCAGATCAACTCTAATGACTTTAGACACTAGTTGGAAATTTATCAATACTAACTCGGAAGCAGGTGTGTCCGAACTCATCTGAGTTTGTGACTTGCTCACATATATTACTATTGGTAATTAAACTAACTCCGAATTGGGTGTCTGCACGGTAAGAGTTCAGTACACTATCGCGAATTCGAACCTCTTCAGGACACAGGCCACTCGAAAAGTCAAGTGTTGTCAGCATGCAATCTGGTTTGAAACTCCAGCAACCACTAACTCGGAACTTGGTGTGTTACTACCTAATTCAAAGTTAGTTTTGATATGCTTCTGCAGCGGCTTTTATAGACTCTTGACCTCTAACTAACTCGAAATCAGGTCCAAAGAGTATAT-937405-**Cdc6i (Plus strand,** 937406-938680**)**

1. **Cdc6h***

1127861-CGGAGAGAGTAGCGGTCACTGTCGATTGATTGCGTCGCAAACGGCGTGTTTTTCGCGGGAACACCACCCTTCGAGTGTTCTTTGGAGGAGCGTGGCTGGCCAGCTATCACTCCAGCGGCTGTTCACGACTGGACAGCCTGTACAGCCCGGATATACCAACCAGACAGGGATTACGCTACTGTACGAAGTCCATGCGAGTGATACTGAGTCGGTACACCACCCTACAAGTGTTCTGACATCACCAGACAGTTGACCCGACACCACCATACAAGTGTTCTATCACCACCCTGCGAGTGTTTCCAGTTCGACTACAGCCAGACCAGAATGGTGAACACGTGTTCACACACCACCCTGCAAGTGTTCGCACGGAAGGTGTGCAAGCGTCTGAATCTACGAAAACACCACGCTGCAAGTGTTTTTGTGGCGTTGTTCCGAGTTCCGATTCGCCCTTGCACACCACCGTGCAAGTGTTTCACGTACTGCTTGAGGTGTGTGAGAGTATGCTGAGACACCACCCTGCAAGTGTTCCAAAAGGCCGTTTTGGGCGGTTTTCCCGTCGGATCGGGTTCACACCACCCTGCAAGTGTTTTCGAAACGGAATACGTAGGGTCGTGGTGGAACCGGACGGACACACCGGTCTGCAAGTGTTCTACACACTGCAGCGGGGAACAGATACCAGATAACCGCCTGTTTGCACCCCCTTTCACTGTCTACTGTGGGGACACACACCACCCTGCAACTGTTCTGGACGCCACAATAGTTAGACCGCCGCTGAACACACACACACCACCGTGCAAGTGTTCTGCCGTGTGAGCCGACGGGGGGTGG-1128688-**Cdc6h (Minus strand, 1128689-**

**1130017)-**1130018-CGTATTCCACCTTTTGAGTGGCTCTATATAAAACCAGTGACCACCGTTCAAGTGTTTCAAGTGTTTTCACGCCGAACAGTCACACACCACCCTGCAAGTGTTTCTCCGCTCCCACTCGAAAACAGTTACGAGCGGCCTGCTACACCGATACCTGACTTAACACACTTGATTCCTGCGATTCTTCATTCTCTAGTCAATGACTAGATATGGAACTAGATATTATTTTTATATCTTATGGTATATGCAATATAGTCTGGTTACTGTTGAATAGCACAGTCTATAGAATCTACTTCGGAAGGGGTCTGTAACGGCTAAATCTATGTATTCAGGTACAGTACCGACCACACAGTCTCGGCGAGACAGAACAATCTCCACTCTATTTAAATATAGTGACGTGGTTCCGCCACTCCCCGCCCCCACCCCCTGCAAACCCCGAGAACACTTGCAGGGTGGTGTGTGTGGGTCCCGCTCTGTCACGAATATTTTGGCAGAAATCTCACTCAATGTCCACACGGC-1130533

1. **Cdc6g***

1400751-CGAGTGGTCTCAGGCGAATCAAGTTTCGGGTGTCATTCTGGTCGGCTCGGAGGCAGGCACGGAGGCCGAGATAATGCAGTGAGAGTACGTTTCGTATGCTTTCGACCGGTGAGGGAGTCGGAATACATCGAGAGTGGATTTCGTGTGCTTTCTACCCGGTGGTGATGGGGCAATCAGTCCAGAGTGAGAGAGTACGTTTCGTGTGCTTTCCCCCTCTTCCTTGGAGAGAGACGAGGATATAACCGATGAGCCAGCGGGTCGGTGGAGCAAACCGAGGTGAGTGCTCAACACCCCACTGTGTCGAGTGTTCTTCGAGAGAGTCGAGGATAGGCCTCGTAACACACCCCACTCTGTCAAGTGTTCGGCGTGGGTAGCTGGATGTCTCCATGCCCCACCCCACCGTGTCGAGTGTTCTTCGAGAGAATCCAAGATAGGTCGCACAACACACCCCACCGTGTCAAGTGTTCGACGTGGGTAGCTGGATGTCTCCATGCCCCACCCCACTATTCCGAATATTACTCTTGATTGCTATCGGTGAAGAGTGATCAAAAGACACTCCACTACATCAAGTGCTAACCGGCAGACGTCGGCGAGCTGTATTTTCACACAATACACTTGACGCGGTGGTTTTATACATGCGGGCCGAATCAAATGCACTAGAGG-1401413-**Cdc6g (Plus strand, 1401414-1402625)-**1402626-CCTGGCGGATTCGGTCGAAGTCTGT

TCGAGTAGCCCCGAATCATCCTGTTTGAACACTCGACACGGTTGGGTGTCGGAGGATATGGTTCCACCCCATCTTGGGATGAACACTCGACACGGTGGGGTGGGGGAGGATGTGGTTCCACCCTGCATTGGAAAGAACACTTGGCACGGTGGGGTGGGGGTGATAATGAACGTAAGAGTGGCGCGGTCAATATTCAACCGGACAACGCCGCAGAACTGCGAACAATCTTAGAGCATCGTGCGAATCAAGCCTTCGTCGACGAAGCGTGTGACACATCAGTGATCGCGAAGGCAGCGGCGCTCGCCGCACAGGAC-1402964

1. **Cdc6d**

2413087-CCTATCCTCCCGCCGCCCGGAGTCGTCCGGTATGGCGGCTCTGCCCGTAAAAGATAGGCGAATTCACTTAAGCCTACGTCAGACATGAGTTTTCTTTTTGACATGAGGGAGCATATCATGGCATAGACCAGCGTGCTGGCGTTGCTACGGGTGTGGAACGGTTGGCGGGGAGCTGTTCGGGTGGAGTCCGGTCATCCGAAACCAGACAATTTAGATTTGGGTTCCCCATCCTAGCCCCCCACCCCTTCGTTTCAAGTGGAACGCCATGCACCCCTGGGGAGGGGTGGGAGAATCGTCGGTCGAATCGGGAGTGCACCAGTTGTTCAGAGCAGCGCGATTCGTGACTTCACTAGCTAGAGCGGCCTAGACACTAGAAAACTGTACACTAACCCCTACTAGCTATTTGTTTTATCTAGTACGGCTTTGGTTTATATGATATCGTATTAAGTCTTTCCCCTTTCTAGAAGACACCATCGGCCTCCCCACCCCCTCCCCCCGGGCCTGTTTCCTGTTCCACCGGAAACGAAGGGGTGGGGGGGTTGGCCTGTTGCAGTCGGTTTCCGCTGCAAGTGCCGGTTTATGATGCACCCTGCGCATATTCATACAGCGTACTCGCTCTTGGAACTGCGCCACCGCTCCCGTTCCTATCCAATCACTCTCCCGCTGCATCATGTCCATCCGACCTCGGTGAGACTGAAGCTATAGTCCTCTTTAGGACAACAGTACCCACCCGACAGAAACAATCAATATAACAACATATATGTTTCATGGGTGGTGTACCGAAATATTTAATATCGATTCACGGTACGGTTCATGTGGTTCAACTGGACGCACCGGAGAGCGCTGATGCTCACTAACAACGGTACTGACGCACTCCTCGCTCCCTCTGTTCCGGTGTCTCCAACTCGCTCCACACGAAACGAAGGGGACCCTCTTGATCGACT-2414030-**Cdc6d (Plus strand, 2414031-2543519)**

**Minichromosome (2)**

1. **Cdc6b**

**Cdc6b (Minus strand, 6826-8064)**-8065-ATTATGCGTGAATGAGTGAGGCTACCACAATAGTTGTTTCGAC

CACCGTTTCCGCTATTTCCAGTATAACCAGGACTGGGTGACCCTTCCGATATATGGGGAGGACCCCACTGTTTCCACTATATCACCTGCGCTCTATCGCGGTGCGGTTGAAGACCAGTAGAATGGAATTAGGTATATACGAGATAACTGCCGCTTGGCGCTATGTGGAGCGATAGTACCCCACTATTTCCAGTATATAGCGTGTCGTGGGGGGACTAGACACACCCCACCGTTTCCAGTAAATCGGAGTCACTGTATCATGAGTTGTGACAGATGGGTCAGACCACTGGGTTCCAGTATTTCCAGTAAATACCTTTTTATAAGAGTTAGAGACCCCACCGTTTCCAGTATATCGGTGAAACAAGTGTAATCAGGAGACTGAAGTAGGATGGAATGGTGTAGATTATTGGTACACCCTCCCCCCACTATTTCCAGTAAAGAACAACGAAGTAGCGACGACTACAGAAAATGAACTTTGATCTAGGGAAAAGTTTATAATAATAGCATTATAGCTAGACCGTAGTAGAGCTAGAGCGGTGTTGTTGTAGGGACTAGTTCTAGTGAAAGAAATGAGGTGCGGAGCCTAACTTACCTGTCCTACTAGCTTTAGTGGAAATAGTGGGGGGAGGGTGGCCATTCTTACTTCTTCTATCCTAATGTGTAATTCTAATAGTAGAAATAGTGGGGTCTGACGGGTGTCAGACAACGGGAGTTGGAACCCGAAACTTCGCGGAATGGCATACGGACTTCTTGGAATACTTGGGTTGCTGGTGTATCTGGTACAAAAGGAGCGTCAAAAGGCTGGCCCGACCTGACTAATATATCTGTCCGCTCGAGCTCCTCACTGATGTCCCGGATAGGGCAAATCGGTACGTTCGAAGGTCTACCTTGCAGTTAGTTCACTCGCTTTCACAGGTGAAGAAACTATATCCAACGACTGGTCTCACAGGCATGGGACCCATTTTTTAGCCCCAGTCTGGTGAGGGCCGTTCCAGTGAGAGCGTCCTGACCGTACC-9122

1. **Cdc6a**

**Cdc6a (Minus strand, 50205-51485)-**51486-CGTATACCCGATAGATACAATCTATGTGAAATAAAACCA

CCGCTTCATGGGACTCATTTGTTTCATGAGATGCCCCCACTGCTTCATGAGACATATGCGTCGGTTTTCACATGAGTTGGGACGTTTTGCACTAACCCCCTCCCCCCAGATTTCACGAGAGTATCGTTAGAGAGGAGGGGAAGAGAGGGCAAAATGAAACTCTGAATGAGAAGAAGATTTAATACAGATAGATTAGAACAGGATCCGTAATACGTTAGGTTCAGTTTAATTCTCTAGTGAGGTCCCAGCAAGAAACGTCAGACATTATTTCTTCCCTCCCCTCCCCTACAGCAACTCTCATGAAATATGGGGGGAGGGGGAGAGATCAACCTGCATGAGTAAGGAGACATCAACTTCTCATGGATTGTCGCTAACAAACGACATCCATCGCTAGTAGCCTATATCCATAGGAATCTACGGTCGCGTTGCTCTTCATTTGATTCACTGAGAGACCATCAATCCCTCTCCACTCACTGTCTGGACTCCGTGCTTTCTCTGAGTTACTACACAATTGATGACCTGCTCTGTTCATACTACACCCCACATTTCATGAGTTAGGAGCTGCGCTGCTATTCTTGAACTCTCGTGAAATGTGGGGGGTGGGGAACAAAATTCCGGTTAACTGGTCCATATTAACTCTCGTGAAAACCGGGGGGTCCCACTTCTCCACGACCGATACAGCAAGACAGGCACTATCAGAGTTGAATTACGCCGCCTGTGTAGGTGTTTGCGTTGGAAGTCTCATCTGGAGGCTCTCATGAAAAGGGACAGATTAAACATAACATCGCAGCTGAAATTATTCAGGTCAGCTATCCTGTACCACATGAGACATCTGTTTATCTAACTGAACTCAGGCCTCCTGTTTGTTCTAATACATCATGTAGGCGATTGAGATATTGCCCACT-52429

**pNG700 (1)**

1. **Cdc6k**

**Cdc6k (Minus strand, 189243-190487)-**190488**-**AGTCGTTGCTTGACGTATTGGAAGGAATCTATATAAA

ACCACCCCTGTCCGCAGTGTCCGGAGTGTCCGCTGTTCTTTTTATTATAAACGATTGGGGGGACCCTCCCACCCCACTATGTCCGCAGTAACTAGAGAAGGGCGAGAGGGGTGAGAGAGAACCTATATATTAGGGAAAGGTTTATTATGGTCTGCAGAGAGCTAGACCGTAATGTAATTTGATAGCTGTAGAAGAGAGCACTATAGTATTACTTGAGAACATACCTAGTCTAGACAAACAAGATTTCAATTCAAATGTTGCCGTTTCTGCTTTCCCTTCCCGTCCTGCCTGAAGAACAGCGGACAAAGTGGGGTGGGAGTGTTATCTCCTATTATTGATCTGGTTTGCAGCTTCCAGCCTCTCCCTGAGTAATTCATAGCACTTCTACTGTCTGAGTACACACCACTGTGTCCGCTGTTCTCCTCTCCGGCCCACAAGAACAGCGGACATCGTGGTGTGACTACGATGGAATAACACCACGATGTCCGCAGTAATTGACTCCTGATTTCTCAAACGCTCAGTAGAACAGCGGACATCGTGGTGTCGATTTAGTATTATGCCTATCTTTTTTCGGTCAGACGCCACGATGTCCGCAGTAATCTGGCAATAGAGACCATCTGATACGATGTTACTGCAGACACCGTGGTGTTCTCTGTGACACACTGCTTGCTCGATGATCTGGGAAGGGCCTCCGTGAGACGTACAGTTCTGACCCCTTTCTGTAATCTCCTCTCAGATTGGTCGACCCCAGAACGCTCGACAGTGTCGTCATCATAATTGCAATAGCAAATACAGAATCCACCATGGCTCTAGCTTCCGGTTCCAGCGTCAATGTTGGCGTCATATTGGTCGCGCTAGACGCTGCAGCGTTCCTGTTCATCCGCCGGGACCGCTGCTGAGCTCAGAATGTCACTGAATGGTGCATTCTATATAGCTTTCAATAACCCACTTGCCAGTCATCGTGATAACCAATAATCCACCCGCGACGTTCTACTACTTTCACTCATTCAAGTAATTCAAACACGGGAGTGGTGCACCGATATCTAACCATCTCTGTGGTAGCTG-191589

**pNG600 (1)**

1. **Cdc6m***

133264-GCGGACACCTGTGACCTGACGTTCGCTGGCGCAGTCGGCTGGTATTCGATAGCCCGGTTGGCGATGGCTGCAGAATGTCGTGGACGAAAGGGGTCCCCCCGGAGTGTGAATGGGACCCGTCAGTCCGACACCCCTCGGTGTGAATGGGCAGCCACCCTCCTCAACACGGCGTTCCATTTCGTCATTGAAAGACGTAGCAAAGCGGAGCCAGCGAAACGGTTTGGCGGATATCCCACCCCCACACCCCTCAGTGTGAATGGTCCCACCACGTGGGGCACCACGATTAAGCTGCTCCTAATTGCGGAACCCGACGAGCGCGGACGCCCCTCCGCCTCCGCGCTGGCGCGTCGGCTTCGGCACAAGTGCCTCGCCTTGAAATAGGATACTACTCGAAGGGCTGTTTAGACTCGTATATATTAAAATTATCTATTACTGCGGTGCGACACACCAGCCAACCACTGCAAACAGCCTATTTTACCGTATTACCCGCTCTACGGGTTGTTTCAACCACACGAGACTGCTAACGGCTCTCCAACGACACCAGCATCATCGAAACAGCTGCCATCCCCTATGCCATACCATTCACACCGAGGGGTGGGGGTG-133866-**Cdc6m (Minus strand, 133867-135135)-**135136-AGCCGGACCAATTCATCCCA

TTCACATATAGGTTGCCCACCCACGGTGTGATTGATTTCAAATCCCATTCACACTGAGGGGAGTGTAACTGATACAGGGTACTCCCCCCGGAGTGTGAATGGATTCGGCTTAGAGAAAGAGATTCACAATCTGAATACATATCACTCCACCATTCACACTCCGGGGGGTGTCGACGTTGTCGCCCACTTGTGGTTTTTCTCACCGAAGACGATCTCCGTCCATTGACCCATGTATCTCATGGGTCCATCTATCGATAAAATAGAAATATGGTGGTAAAGGGGCGCTATCGACATCTCAGAACAAGGTCATCCGTT-135470

**pNG500 (2)**

1. **Cdc6o**

**Cdc6o (Minus strand, 26102-27328)-**27329-AGGGAATCTTTCACAAGAGGGATATATAAAACCACCTC

TGCATCTGTTTCAAGTGAAGCCACTCACACACACCACTGTTTCGAGTGAAGAACTAGACTAGAGTAGTTACTAGAACAACAGTCCACTGTTTCATCTAAAGCGCTGACACTAGTCTAATTGGCCAGAATCCCGATTCTGGTAGTAGCGGTATTGAAACTCTTGTTTTTAGCTGCTTTGTGGTCAGTGACTAATTGCTGGGGTTCCACAGGGCCTCCACCCTTTCCCGGCTTCACATGAAAGAGTGGTGTGTCTCTCCCCCCGGGGTTTCGCTGTCCCATCTCGAAGTTGATGCTCTCAAGCTAACCAACCTCTAGCGTCAGTTGGGGAAAGTGTTGGTTAGTTATGGCCGCGACTGTGGAATTTCAGGCGCCACTGTCTTGACGGACTATCTGACAGTGTTGTCAGAAGGAACAGAGGGAGGATGTCAGATAGAATCGTTCGCTCTCAGACTTCTTTTCCTACACCTCATAGAGGGAAGGAAAAGGGAAGTCGTTTCACGTCACTCTAACGAAACGAGAACAAGCGTAGTTTACGCACCACATTCGTGTGGATTTTATCGCAGGGGTCTAGTGGGGAATTCGTTACCGCTCTTGTCGACCACCTCCATCTGACACGCTACTCGCGAGTGTGCTGACAACACTGTCAGAACGCGTC-28021

1. **Cdc6n^#^**

105429-GTACGCACCTCTCGAAATTTTCGAAGTTTGCGAAGTTTTCGGGCCATTCGTATATGTCGTATATTACGCCATGTACATAAATTCCAGTGCGACAGATATAAGACATATATTGCGCAACTTCCGTAAGGCTTGGAAGTTGCGGAATATCCGTAATATACGTAGTGTCCGTAATTTCCAGCTATTTCGAATAGTACGGCTGTCACTGTCGAATCAAGGGTAGCTAACTGATACTTACCCTACCCCCATCGGAGTCGAGTCCGGGTACTAGATTTAGCGTCTCAAGCAGTTGTCAACCACTTTTCGGACACCCACACACCGTCTTCGAAGTATCCTCCTGGATTGGTGTTTGTGGAGAATAGTCGATAACCAACTCATGCGGTGCAGTTTGAGGAATAATGGATAATTGAAACGGTAGAAGATAAGATATTAAATTGACTATTATAGGAGATAGATTTAATAAGAAGGGAGAAAAACCAAACCCGTATCACTACTTGAGTAATAGTATGAACAGACTAAGAATCATCAAGAGCATCGTAAAGTATGCCCATAACTCCACTACTCGCTTATTTTAAGCAAAGATACTTCGAAGACGGTGTCTCTCTCTCTATCCATTCTGATTATGCTTCAGAACAACTTATCCGTGGAACCGGCCACAAAGATACTTCGAAGACGGTGTGTGGCCCCCCCTATGTAGATATG-106127-**Cdc6n (Minus strand, 106128-107549)**

**pNG100 (1)**

1. **Cdc6q**

19157-AAGTGAATAGCAATCAGAGGGTAGTAGCTGTTCGCATATATGTGGTTTATATCGATGATGCAGAGCTTTGTAGTGAGGATAGGGTACATATCTGCTTTCGAATAGTTTGGACAAGACACACACACCAAATTTTGAGTAAGAAGATAGAAGACTGCGGGCTATGAGGCCACATTACGGCACCTCGGTAGCTGTTCTTATTCCAAACTAGGTGTGTCTCTCTAGTCTAGTTATTCTAGTATGTAGTGTAGCATATGTGTACAGTCCTACTAGATGGACTGAAACAGTCTTAGACGTGTCCTCCCCTGTCACACTTTATCTGTTTCTTACTCCAAACTAGGTGTGTGTGTCATACAGTTCCTTACTCAAAATCTGGTGTGACTGGGCCAAGTTTTGTGTAAGGCAGAAGGCTGCTTGTTAGACGCCAGAGTGTCGCTTAGAGGGCTATTACTCATAACCCGGTCCGAAAGCTTATTTCGTGTCTACTGTTGTCTTTTGATAATGGATAGTGACG-19668-**Cdc6q (Plus strand,** **19669-20877)**

**4-5. *Halobacterium salinarum* R1** (AM774415-19) **and *Halobacterium* sp. NRC-1** (AE004437-38; AF016485)

**Chromosome (3)**

1. **Orc10***

38987-CCGGTATTCCCATGCAAGGAAGCCGCGCCGTTCACGGCGCGGAGGATGTCACTGTTGGGTATTAGGTTTTCAGCCAGAGGAGGATATGTTACTGATTCACTTGTATTGGTGGTGTGTTGTTCTTCTAGTTCCTCACGTTTCGATTCACTTGCAACAGTGGTGTGTGGGTGTTCACTTGCATCAGGTGAAACGGTGGTTTTATTATCCTCTCTCGGGGTCTTGCTATCA-39214-**Orc10 (Plus strand, 39215-40447 in HR1)**-40448-C

GATTGTGCGATGGATCTCTTTGTATTGCCGTGGATTCATGCTGTCCGGGTATTCGGCCAGGAGACATCTGGACACACCACTACTGCAAGTGAAGTTGATGGCTAGAGGTCGCCTGTGGCGCACTACGGGAGTGTTTTGATTATTGTTGGATGTCTAACTGC-40609

1. **Orc8***

911958-CTCTGCCATACTCCCTTGGTCCGCCGCTGGCCTCAAAACGGTTTCTCAATCCGGCCGCTGGTCGCGGTCCCCCACCCCAAGTCTCAAGTGTTAGTCGGCGAAACCCCGGGCCAGATTGAACAGGGCCTCTCGGTCGACAGTCCGTTCCCAGTAGCGGTCGGGACGCGACTGTGCGGCCGGCTCGCCGGCTGTGGGCGGCACGCGATCCGACTCGGCCGGGGTCAAGCGAGTGGCTCGGGCTGGGACGGGCGAGGAGCACGTCGTGTTGGCGGTGGTGCCGTAACTGCCCGCGAAGGACGTCGAGAAGCGGTGAGGCTGGGATTCGAACCCAGGAGGCGTGACGCCACCGGTTTTCAAGACCGGCGCAATAGGCCGCTCTGCCACCTCACCGTACACGACGCGATTGAACGGGGGGAGGGTTGAAACTGTCGTTGTCCGGCGAGCCCGGGGTCGGCTCTCCCACCCACCCCGTATTTCGAGTGTTTTGCGGTGCGGACACCCCACCCACCCCCTCCCCCACCCCGATCTGCAAATGTTCGACTGTGTGCTCCGGGGGCCACCCCCCAACGGTGGCAGGGATCGGTGACACCGTGTCATCGGTTTGCACTGCCGCGGTCCGGATCCCGACGAAACACCCGAAACAGCCGAAACACGAGAACCAAAGTGTTATGAAACGGTGGGTAGAAACTCTCGGCAAGCG-912657-**Orc8 (Plus strand,** **912658-913851 in HR1)-**913852-ACCCCGGACTGTGCTCTGGAGAAACACTCGAAATCCGGGGTGGGGG

TGTTCGCCCGCACCATGTTATTTGCTGTCATCCTGCAGTGGTCCCCACACCCCACCCCACCCCACCGAATCAGCACGCCCAACATTCGAAATCCGGGGTGGGGGGG-914003

1. **Orc7**

1794670-CCTTGCTCTCCATTCGCACCCCATGCCTTGTATGTTTGACGCAGGACGTGTCTGGATGCGTATAAAACCCCAGTGAGAGGGCGTAAAATCCGTCAGACGAGGGTCTGACCGTGGTCAGATCGAACCCCGGCTCCGGGATCGACGCCGAGACGAGAGCGCACCCCGTCAGCCACGATGCAGCCACACCCACGACAGGCGATATCAACACACACGAACATCCACAACCCCCCACCCCTTCGTTTCGGGTGGAACTCCAGAACAGGGACGGCCCCGGCCCACCCGCCAGCCAAGATCAGCGCCCGAACGCAACTAGAACTAGACAACTACCGTATTCTGCTCCTAGTTTTTGCTAGTTTGCGGTTGTTGTTCTGATTGTAGGGACTTAGTAGTACCGCTACTCTAGAAGGGGGGTGGGGACCCCCCACCCCACAGAAGACTCTCGTTCCACTCGAAACGAAGGGGTGGGGGGTTGGCAGCCACGTTTGACCTCAACCTCTGTTTCAGGTGGAACGGCGATTCATCGTCTTTCTCCGCGGGTCGCCCCGGCTGGTTCCGGACCGCATCCCTCCCCCCTCTAACCGGCGGGACATCGGATGGCGGGCGCCCTGGTATCGGGTTGCTGTGATCGTGCCTGCCCGGAAGCGTCCCCGCTATCGTAACTTATTAGTCCGACTCGGACCCACTGTTTCATCTGCATCAACTGGAACGCTGTCGGAGCGGGGACGCTATACCGCCCGCTGATGGCCTTGCTGGCCGTTCTCTCTTCCCGACTGCGTATTCCACCCGAAACAAGGGGGTGTGCGTGAATAC-1795479-**Orc7 (Plus strand,** **1795480-1797036 in HR1)**

**pHS1 (1)**

1. **OE7115F**

78370-CCCTGCGGTTGCAACAAGATACAGCTGGAACCACACACCCCCCTCGTTCGGAATGAGATTATACCCTCCATACCACCTGATTAGAAGCTGAGTTTCCACCCGATGAGAAGGTTGCAGTAATGGATTCGTTGGGTATACGCCCTCGACGTATGGTCTACTGCTTCGATTCAAAGGATAGAATAGCTGTACTTCCAGTGTTAGGCGTATTCACGAATACTTCTAACTAGTGCCGTATTCTACTTAGTGGGCTCTTAGCGTAACTAGGTAATAAAGCTAGGGTAGTAACTAGACAAGTGATCCTTCGAAGTCTACGCTATCTTGCGGTTTGGAGAGAATGGCTCGTCCTAGTTAAGAACCGCCTAGTTGGTTGATGGAAGATCGCGCCCATACGGTCAGTCTTACGGGATCTCATTCCGAATCAGGGGGGTGTGTCCCTCCAACTCCAATCTCCATCCATCCTGTTTAAGCTGGCATCAATATCGTCCATTAGTTCGGGGAACAGCTCACTCCGAATCAGGGGGGTCTCTTTAGCATTCCCGATTCTTGGCTGATGGACCAACTCGTACTACCTCATTCCGAACGAGGGGGGTTGATCTGAACCCGCTGATCATTTGAGCTGCTGGCAATCCTCTCGTTCTAGGAAATAGCTCACTCCGAATTAGGAGGGTGAGGTCATCAGGAGACGCAGATTTGAACGACACCTCACTCTGAATCGGGTATCATCGGGTATGGCGGTGCCACCTATCGATCTTCCGCTGTTTAGCACCGATATCCAACGAATCTCATTCCGAACCAGGGGGGTAACGACTAAGTAGGTTCCTTCCCAGGATGAATCT-79205-**OE7115F (Plus strand, 79206-80600)**

**pHS2 (3)**

1. **Orc4**

**Orc4 (Minus strand, 23146-24378)**-24379-TGAATATCACACGGTGTGAGCGGAGGGAGTATAAATCTAG

TGGAGAACACCACCCCACGTTTCCGACGTTCCTTGTCCGATATCAGCCGGAAAATGCCGTAATTCGGTGGAATTGGATTGCTGACAATATAGCCGCGTTTCCGACGTGAGAGAGGAAACCCGTAGACGGAAGCGTCTCCTCGATGTCGAATCCCGAACCCACACACCCCACGTTTCCGTCGTAATCACCGAACAGGTGGGGAGGGGACTCGAAGAAGACATCCACGACGAGATACCAAAGACGAGAGCGGCCTAGCCGTCCAATACTGGCCGTCTACAGAAGCAGCTGGAAAGGATCGTTTCATCTGAGGGGGCGAAGTACTTCGCCCCCTCCTTTGTCGAAGTACACTTAATGAACGTTTCTCAGCTATGCCACTTTAATCTATGTGTTGCAGAAGTTAGTTCATCTCTAGATTGGTTCTAGGTCAACATAATTAGTGGTTGCATACCACTATCGTAGCCTCTCCCTACGCCCTCGTGGAGAAACGACGGAAATGCGGGGGGTGTGGTCCCTTCGAAATCAGCGTCACCCCCTCCACCCACATCCAGTTACGACGGAAACGTGGGGTGGGTGTCCTCCCTCATCGAGTTTTACTCGTCCGGGTTGACGGGCCATCCATACTCTGCGGCATCTCGTCGAGTTCTGTCCTGAGCAAGGTATCTTCCTCCGGACCTGTTC-25096

1. **OE6235F** (128645-130039)

This origin is the same with **OE7115F** in pHS1.

1. **OE6288R**

**OE6288R (Minus strand, 154689-155954)-**155955-AATGGCAACGTGTGAGTGGTGGAGCATAACCCTGT

CGTCCGAGTGGTAGGGTAATGTCCAGATGATGGGGGCGCGTCCGATGTAAGCTGGTGCGGCGTGGTGTTAGGTCAGATTGATCAGACACGGGGGGACGGGGGTGTGTCCAATGTAAGAGTAGAGCAATAGGAACAGCGACGCGATTACACGGCAGTATCGACTTTGATCAGGGAAACGTTTATTACACCACTACTCAAACAGTATCCGTACTCCTCATGACGTTGCGGTGTAGTGTAGATGTTGGTTGCTGGTTGTGCAGGTGCGACTGAAGCTTCGCGAGTTTACATTGGACGCACCCCCGTCTCCGGGGGTAGCGGAAGTGGTCTTTGTGTTTGAGGCGAGAGCTTCGACAGCCGTGTCCTCAATCAACGCCACCAACAGGGAAACCGTCACAGCTGGAGGACGCCTCGATGTCGGGATCGTCGACGG-156419

**pHS3 (1)**

1. **Orc5^#^**

93337-CGGGGTCCCGTACCACCGTTCCTGCTGGGCGGCCCGCCGAGATTCATGCGGGAGCTGGACGTGTCGGCGGCGAAAAGGGAGGGGGGACTCCCTCCCCCCGTCATCGATGTGTAAACGGGATTGCCGCAGGGTGGGGGGATCGGTCGAACCAACCAGTAGGTGAGCTACGACCGCGATACACGCCGAGGACAGCTCCCTTCTGCCGGTTTTGTCCCTCTCAGCGAACTGTATTACTATTAAGGAAAGATTTATAATAGACCAGGGAAAACAGTAACCGCACTGCAATAGCTGTATGTTAATTTGGTAGTTGTAGGAGAACACGTAACTAGTGCTTGACAGCATTCGAACTCCGTCTAATACGTCGCCTTCCTGTCACAGCGGCGTTGAACCCTGCTTCTGTCTTGGTCTCACTTCGTTGCCAGTGTGCCATCGATCCCTCGGTGGTATCGTTTACACATCGATGACGGGGAGGGGGTGTGGCCTTGCCACGATCGCCAGCAGGAGCCGACGATCTGATCAGAGACACGACAGGAGATTACACATCGAAGACGGGGGGTGGGGAGGGAAGCGAAAGGCAGCAGGGAATCGCCTGGCAA-93932-**Orc5 (Minus strand, 93933-95327)**

**pNRC200 (2)**

1. **Orc5^#^** (210588-212033)
2. **Ori4** (291390-292622)

These two origins are the same with the corresponding origins in pHS3 and pHS2, respectively.

**6. *Haloferax volcanii* DS2** (CP001953-57)

**Chromosome (4)**

1. **Orc1**

TTGTTACTTTGGGCGTACACGTGCGTAACTCGAACGGATACCGAGCATGACTCCGGAGTACCGCAGTGTCTCTCGAACACGACGAGCGTTCTGAAGCCGCCGCGCCGACCTCCCCACAGTCTGTCCTCGCCGCAGTAGCCGTGATTCTGATATAGTGACACGTGCCGCGGGGACGTGAACCACTCGCAAGGCAACACCCCCACCCCTTCGTTTCAGGTGGAACGGGCCGAAGGGGTGGGTGGGGGGAGACCGCTAGACGGCGGTCGGATGTTAATTGAATAGAGAGGGTGAGTAATATTATCCATTTTCTCTCTACTACGGGTTTGATATGCAAATATCTGTGATAAAACCACCGCTATACAAAGACTACCGAACCCCCTCCCCTCCTGTTCGCGGCTCCACCTGAAACGAAGGGGTGTGGGGGAACCGAGACGCAGGGGTTTCCCGTCGAACTGGAGAACGACAGTTAATTACAAGAGAAATCGAGACGTTACACAAGGTCAATACTCGCTCTACTACCCGAATTCGTCCAAATCGTTATTCGGACATCTCCACGCGAAACGAAGGGGTTACTCCCATCGAATTTTCTGTCGGGGACACATCGCACGTCGAGTTCCACTCCAACGACGCCCGTTGGTCAGTCACCGCCACCGCTCAGTTACTGTCGGTACTCGGTCTCCGACACCCATCCGGCTACCCACCATCGTTCAGTCACGCTGCCGCGCAGTTGCCGGCGCAACTCGTTACCCACCACTCGGCCGACTCGACCTGTTTCGACTCGAGCCGAGCCGACTTTCCGACGCGGCTCGATTCGCCCGTTCGCCATCTGGTTCGTCTCCCAGCGACCGCCGGTACTGACGACATCGCCGGGGGAGCGAAACTGATACGCGCGGGTTCTCGTGTTGAAACGGTCCCGCCCGGCCGAGTTCCGGTCGAACGGCGGCGGACGGGTCGGAGAAGTAAGGTTTTTGTACCAACTGTTCGTTTGGTTCCTTTGCATCATCTGGAATCGCCTTCTTCGGCGTGATGCGTAAATGCCGACTATCACCGGCTTCACGCACCCTATACGCCGCCGCGGGCGACTATTCCACTTGAAACAAAGGGGTATTCACG-257-**Orc1 (Plus strand, 258-1952)**

1. **Orc2**

**Orc2 (Minus strand, 569203-570429)-**570430-TAGCGTCGGTCCGTTGTAGCGTCCCCTTATTTAAAACCC

CGATTTCATCTGATTCATGTGAATTGTGACGACGACGGTGTCGCCGGAGTCAGACCGCGTGTTCTCGTCTGGAACCGCCGTCTCTCGGTTGATGCTTGCTCACCCTATACTGTCCCATGTTATTGTATTTCGTGGGTCATGTGAACTCGTTTGAACAGACACACACCATGTTTGCAAGTGAAAGAGAGTACGCGAGAGTAGCTAGTAGAACAACAGAGCATGTGTTCAACTAAAGCGTTGACATATTTCGGTGCTCACGGTGTACGTCTCGGTGGGATGCGTCTCCCGTTAACGAGGCGTAGACGGCCTCAATCGGAGTGAATCACCTGCAATGGTGGTGGGTGACGCGCGACGAGTCCCGTTCGAGGTTCACTTGCACCGGTGCTCTCCCCGCGCAGTCGCCGGTCCCACCCCCTCCGCGGCGTCACCGCGCTTCACGTGCAACGGTGGTGTCTGTGGGTACGCCGCGACCCATCGCTTCCCCGCCAATTCACTTGCAAACGTGGTGTGTGGGTGTCGGACCGCGGCTTTCGCGTTCCGCCGCGGCGACCCATTCACTTGTACGGCCGGTCTGTCCAGCCGCGTCGTCTGTGACCCTTCGGCACCACCGACACGGCGCGACCGACGCTCCGCTCGACTCGACTGGTCGACGGGGTGACAGTCGTCCCGCCGGCGACGGCCCCGCCCCTCGAAATGTCTTTACCTCGG-571176

1. **Orc5**

1593326-ACGTTACCGATTCGTCCCCCTGGGTAATTGCATTGACGCCACGGGAACGACACAGTCCGTCGGGGGAGAACGTTTCGAGGCTCGTTCCGGGAGGCTACGCACGGATGCACGAGAGTCGCATGCACACCACGTTTCCGGTGAACGAGTGAGAGCTAAGTTCGGTCCGAGACCGGTGTAGACGTCGGTGTCGGCAGGTGACGGCTCGGCCGGGTCCGGTCGGTCGTCGACGACTCACCACATCGTGTTCGCGACACCCCACACCTCGCTTCCGGTGAATCCCGAGACCGGGTGAGACGGGTAGAGACACCACGTTTCCGGTGAATGAGGGGAAGATAGAGAACCGGAAAACCCGGTCACAGAGCCGGGATACCACGAGCCGTCAGGACGGAAAGCGACAGTCGGTCGCTCACCGGTCAGCTCTCCGTCGCCAACTTCGTCCGGCTCGGCGGAGAACGTAGCAACGCTGACAGACACCGTGTTTCCGGTGAAATACGATACAGGGCAGACGAGTCCCGCGGGGGTCGGAAAGCGGTCAAACAGCCGATGAGTCACGAGAGCGAGGTCACGCGCACACCACGTTTCCGGTGAACCGAACCACGCGCCTGGCAGCGGCAAACGCATGGAAACACGGGTGTGCGCGTTCGTATCTTTCCCCTCATTTTCGGGGGCACCCGAATCGAACCACTTATATTGAACACACCGCGTTTCCGGTGAAAACACCTGCAATAAGAGTTCTATCTGGGGAATAGGGACACTACGGGAGACCGAGATGAGACACCACATTTCCGGTGAGTCAGCCAGGCGAGTCCATCAGGCGCGGTCCAACACTCACTCGACTCGCGTCGGGGTTCCTGTGTAGTTCAAGTAGCAATAATTAAATAGTATATTCGTGTTAACGACCATTGATACACTCGTGAAGTAAAGAAATACTCGTAACTCGAGTACGGTTCGAATCGAACCGTTCGTCTTAGACGTGTGTTTTTCGGAGTTTGCGAGTCAGCACAGTCCGTGATTTCGGTCCGTTCTGGAGGGTCCTACGAGGTTTCGCGAACTCCCGTGAATCTGAGCGATTCGTGGCGAGACGGAACTTCACAGTCGACTCTCGTCACAGACCGCTTCCAGCAACAGCGCACACACGGCATCGGTGCCGACGTAACTCGTCAGCTTCGTGCGCGTTCTCCGTCCTCAGGTGGGACGCGAAGTGAGAGTAGCCGGCCTCGTGGGGTCAGGTAGACTCCCCCACACCGTGTTTCCGGTGAATCAATACCTCCGTTTCGTGTGGACGGAAAACCGGGGTTCGTGGGCGTTCACCGGAAACGTGGTGTGTGTGTGCTGAGTCGAATGGTAAGTCCTTTCACCGGAAACGTGGTGTGTTGCGAAC-1594706-**Orc5 (Plus strand, 1594707-1595912)**

1. **Orc4***

1889700-ACTCCCGAAGGTGTGAGGGCGGTCGGTGGCTCACGAACGACCTCCTCCGTCGGTCTGCCGCCTCCACCGAGTGATACGAACGAACTGCTCCATCGTTCCCGCAGCCAGAGCACCGACGACCGAGCGAATCCGCTGTTCGACGGCGACGACTCGAATCCGGTCGCGCTCGACGACGCGGTCACACCGATGCGACGGACACAATCACTAACTCAATTGACGTTGGATACTGTTAGCTGTGTTCCCAATCTCACACCTGTGGTCCGGCGTGTCCCTCCAAACACCCCCCTTTCCCGTCGGCGTGCGCGAAACGCACGTGACCGGGCTCGTGGTGCTCATCGGACTCATCTTCGGCGGGATGTATCTGTACACTCGCTGGCGTCAGTATCTTCGGTAGACACAGCGACGGACCGCGTTGCACCCTCCCGACGAACAATACCACCGTCGAACTGACGGCCGAGTCAGAGAGTACAGTTCGTACGCTCTCCTCTCTGCTGCCTCGCGCTTTCAACGACGAACGAACGCAGGCGAACTGTACTCTCTCCCGTCCCGCTTCGGCCATCGCGTTCCACGTTAGAGAGTACATTTCGTATGCTTTCCTCAATATGAAGCGTTCTACGAGGGGGTGGGTGTGGAGTTTTGAATGTATCGCGTTGTACAGGTCACCGCAGATGCGATGACGACATGACGGTTCCTCGGGGAGTACCGTTTCGAGTGTATCTGATCTCGTGTCCTTCGCATCGGCCTCGGAGTCTCTGTTTCCGATTTCGTGTGTACCACTTCCTGAGACGCATCTAAACAGATGAAGTCGTGGGGGTCAGATTTTCGTCCCGGATTCTCAGAACGATTTCTTTGCAGTCTATTTCCTTCGTCTCCGGTATACGAACTGGTTCAGTTTACAGTTTGGTTGTCACCCCCCATCGCTTCATGTGTTCTTGTGGTTCGTCTCTATCCACAGCAGCGCTGCACTACCACCGGTATCTCCGTCTCGAACTGGCTTCGACTCGAAGATATTGGCATCTTGAACCCCTGTATGGGTGCTAAACACATGAAGTGGTGGGGGTAGAACGATTACCAAATCCTCTCGGCTACCTGAACAGCCATGACCAACAATGTTGGAGACTCTCCCACCCCACCACTTCATCTGTTCTGATTATCTGCACTGTTTCTGTGTGGTTTGGGGGCTCAATAGTTCGGGTGGTCGCTGGCTCTTAACCATCATTGTCAGTCGTAGACAGATCACACAGCATAGAACAGATGAAGCCACGGGGTTCTTCACTTATATATTCGGAGAACAGATGAAACAGATGAACCGGTGGATATATCTGGTATACAGGTCTGCAACACGT-1888353-**Orc4 (Plus strand,1888354-1889583)-**1889584-CCCACAGAACAG

ATGAAGTGACGGGGTGTGGGTGGTCATGGCTCCGTTCTGTCTCTCGAAAAAAACAGATGAAGTGGTGGCGTGCCCCGGTCCTCAACTGCCGTCTCACGCGACGTCTGCCTTCCTACCCGAAACCGGTGTTTGGTCGTCTCGTCTCGCGCCGCGAGGGTTGCGTGCAGACCGTCAGGACCCAGTCCCGAAACCGGTGTCTGTTCGGCTCGTCTGCTTCTGCGATCGACGCCTGGCTCCCATAGCCCCCGAATGCGGTGTCCACACGGATGTTTCTGACTCGGAAGCACGCGAAATGCCCACTCTGTATCAGATCGTCTCAGTCTCTCAGTGGATCCTATCTGCTGCTGAAAGACAGTACATCTTGTGTGCCTTTCCCGACTGCATTGGCTTCGATTGGTCGACGAGTCACCCCATGCCGATATCGAAAGAACAGATGAAGTCATGGGGGGAGGGAGGACCGGATCGGACCGTTCTGTGTGGCTCGTTCGACGGCGCGGTTACGGTGATACGACGGACACAATCAACTAACTACATCGATGCTCGCACCGCTATTCGCGTCTCGCACTCTCCCCCCGTGGTCTGGCGTGTCGCTCGGTCCCCAACGAACA-1890203

**pHV4 (3)**

1. **Orc3**

ACCACTTTGTCCGCTGTTCTCGGTGCGAAGCGGTACCCCCCGTCGGCGTGAGACGGCTCTTTTCCACGGATTTCCAATCGGGAAGGTTAGTTTAGATGTATAGATAAGCGAATAAAACAAGCCGTCCTCAAACAGCTACTATATAACTATTATAGTTGCCCGTATTACGGACTTAGTTTAAATTATAGTATTATAAACCTTTCTCTATATCAAGACAAACAAACCCATCCACTTGTTCCACCGGTTACCGTCGTCTCCCAGAACAGCGGACAAAGTGGAGGGGGTGTGTTCCCGCCTAAGTATATAAAGAACGAGAACAGCGGACACTCCGGACGCGGTGGTTTTATCAACGAAGTGGTCGAACCGTGGTAGT-151-**Orc3 (Plus strand, 152-1402)**

1. **Orc13**

**Orc13 (Minus strand, 55398-56897)-**56898-TATGAACCCCCTCGAACTCCGGATATAAAAATATAGGCC

CTTTTAGAGTTGTAACCACGCGAAAACAGGCTATCTAACGGCTATAGCCCTGCATCCTCGAAATATGCCAATTACCGTCTTCGGAATCTATCACCCTCCGTTGTCGAGTTGTAATCGATCGTCAGGAATCGCTATCATCGAAATTCCTCGATGACCTACACCCCCCTTTTTCGAGTTGTAAGCCTTCGAGGAGCGGTAACATACCTGGTCCGGTTTTCCCATCCGCCCCATTTTCGAGCTGTAAGCAACGGTGAGTCGCACTAAGTATAGAAGCAAATCTGTACCGTCAGGGTTTTGCGAATGATGCTCAGGGTGAAACGGTCTATCCCACGCCCCCCGTTTTCGAGTTGTAAGCCCCAGAATTGCCGTGAAAGGGCGACTCTCGTATGACCAGTTCACGAATACGAGAAATACCGGCCTCCAGATACGATGGTTGGTGAAGCTCGTTTAGTGAACTCGACTGTTCACCCCCTCCCCCCTTTTTCGAGTTGTAAACTCGTGATGACGGGAGGGGGGTGAACAGCAGGAGTGACTGCAGAACGGTCGATTGTGGCGTATACATACGATAGGGGGACTGAGGATTGATTTCTCTAACTTGCAACTATAAATGCGAAAGTTTATCCCATAGACCACCTTACCACATCCGTAGTAGAAATAATGAAAATATATCTTCTAGCTCCGCTAGAAGAGTGAGATCATTGGGTATGGAGATCATCATATAATTCCGAACCCGAGAATAATCACGCCTCAACGCTCCTACGTCCAGTTTTGTGAACTCTGTTCTCACTACATAGCGTCTGTAGCAGTCTGCACCCAACGACTTACAACTCGAAAAAGGGGGGAGGGGGTT-57786

1. **Orc7**

**Orc7 (Minus strand,** **257391-258668)-**258669-CGATACCAGATACCGCGCAACCTGGCTACTTAATTATT

CTCCCACCTCGACAAGAGTGTAAATTAGCCCATAACTAGGACCTACTGCCTAGAGCGTCCAATGTCAGTCGGTAAAAGAGACGAGGTAATCCAGAGTGAAAGGCGTCCTTCCCGTGCAAGTGATTTGGACGATGAAACACCCCTCACCAAGACTGTATAGCGGACAGAAACCAATATTTGACCACCCCTCGTCCCGAGTGAATTACCACTAATCTTCCACTGGGCTCGAAAATGCACACCCCCCATCAAGAGTATAACTCAATCAGAGCAAAGATGTCAAACATTGAGCAGACACAAAGACGCCTGACGCACGGCATACACCCCTCGTCCAGAGTGAAAACAGTCGCGAGGTTCGTTATTCGGTTGAACAGAAGTTCGAGGAGATGCTTCCTGTTTTCAGTCGTAGCCATCGATACAAAACACAGCTGTTGACCTGTATTCTGCACCAATCTTGAGGGTATCGAGAGAATAATCACATAAAAGTCACGACGTAAAGACACCCACCCACCCCTCGTCCAGAGTGAAACAGCACGAGAAGATCCGGGTAGCAAGATGAGAATTGCACGACTGTATCGCGTGCAAACGACACGAATCACAGGAAGAGAGACGGAAACACACGCATCGAGCTTCCGGGGAATCAACCAGAACGCACATTTATCAGAATTCTTAGTAATGTCTAGAAGATTTATATTTTATTGGTTCGAATAGCCCACTAGCTACATTCTCCACAACAAAAAAAGACGAATTAGTATTCGTCTGGATCTCGTGAAGAACTACTCATCTACTTTCCTCTTATTTCGCCGTCAATCTCTGTCTTAGCGACTCTCTCCGTGGGTTCGTCCGTGTTCTGTCTCTTTGACCTCTGTTTCTTGTGGATAACACCCCTCACCCTTTCAATCGTTTTCACTCTTGACGAGGGGTGTGTGTTCCCACAATTTTCTCCTTCCGGTTCAGAAATTCGAGTTAAACTCTCGATTCCTCGCTC-259691

**pHV3 (1)**

1. **Orc6**

GCACTCGTCGGGTACTCGGTCGGCGGTGAGCGACGGGGTGTTGTCCGACGTGTTGGCGTGGTTGGGAGGTTGTGAACTGGAAGTCGGCAGACCGGTCACGGATTCGTTGTTCGTTGTTTGTTGTTGGATTTTGTGCTGCTTTGTTATTTTGTTGTTGTTATTATTTTGTTGCTGTTGTTTTATTGTTTTTTGTTGTGTTGAGTAGTAGAACTAGTAGTCTAGCTACTAGTGATAGTGTGATGTCGAGATACCATGACTACTCTTCGAGGACCGGGTGTCACTCACGAGTAGACCATCTGTCCTCGGGAACACACACACACCCCCCTCGCGTTTGTAACGGCTACGCCAGTCCCTAGGGAGATTCGACACACCCCCCTCGCGTTTGTAAATATAGGGGCAAAATCAACCGATAATTCCTCACCCGTTGACCCACCCCCCACGCCGATTCGCGTTACATTCGCGAGGGTGGTGTGTCTGCACTGCCCCTTTCACCACTCGACGTTACAAACGCGACGGTACTCCCCCGTTCGATTACAAACGCGAGGGTGGTCTCTCGGAGGCCGAGTTACAAACGCGAGTGTACCCCCCACCGACTGTGTCTTCCTCGTGACCGCCTCGGCCGTTACAAACGCGAGGGTGGTGTCTGACGGTCTTACTTGCGCGATACTACTCGTTGATCGTCGAATCCAAAACCGGGTCCGAAGTGCCGTTCAAACGGCCGTATTTGTCGCTGTTACAAACGCGAGTCCACCCCTTCACATTTATTACGGGTGAGTATGAATACCGAACTCAC-200-**Orc6 (Plus strand, 201-1433)**

**pHV1 (2)**

1. **Orc10**

GACGGCAGTCGGATTGGACGAACACCAACACACACACACCGCGTGTCGAGTGGCTAGGGGGAAAACGGGGTCGGGGGTCTGACGAGAGTCTATATATCAGCAATACCTTTACATGACAGTAGATATAACAGTAACCGTACTGGGAACAAAACACGTATAATTACGGTTGTTGTAGTAGTGAATTATTAACAACCGAGCTTCCGCGTCGCGTGGTTGACCCGACCCACTCGACATACGGTGTGTGTGTGTCCATTCCGACCGTGATTCCAACACTGTCTTGCGATCTTCACCTTTTTTCTGCCGACCCACTCGACACACCACCCGTGCCACTCGACACACTCGTCACGGCAGTCCCCTGCACTCGACGCACTCGACACGGTGGTGTGTCCATTCGACGCACTCGACACGGTGGTGTTCCCCTGCACTCGTCACACCCGACATGGTGGTGTCCTCACTCGACGCACTCGACACGGTGGTGTAGGTATTCGACACACTCGACATGAGGGTGTCCACTTGACGCACTCGACACGGGGGACCGCCCACTAGACACACTCGACACGATGGTGTCCACACGACACATTCGACAATCTAGACCCACTCGACACGGTGGTCACAAGGTTTAATGTGGTTGACTATTATGCCTTAAACAGAGGTTGGCACAGAACCC-100-**Orc10 (Plus strand, 101-1327)**

1. **Orc8^#^**

**Orc8 (Plus strand, 56360-57616)**-57617-CAGCCCACCCACCCCTCGTTTCCGACGTTCATTCCAGCCGTT

ACCAGTTCCTTCCATCGGCGGTGGCCACACACGCACCCCTCGTTTCCGACGTTCTTTGTATAGGTCCACCCCAAGGTGGGTTTCCGCTCTTATTCTAGGAGCAAACATTATTACATATGCGCCGAGTATCACTCAGTAGCCGCTTTTTAATTTTGAATTGGGTGGGACATAGTCCCGCCCATCAGTGAGTTTAATTCACCCTCTCACGAGAACGTCGGAAACGTGGGGTGGGGGTGTTGGTGTGTTGACGTGCAATCACATTCACGTTCTCCTACGAGGGTGTGTAGACCTGCCCCGGTTAGGTCCGAAGGAGCCGATGACTGAGAGACACAAAGACGGAGTCTGCTTAAATTCCTCGTATGGTCAGGTTCACGCCCCAGCTGAGATGGTATGTGGGGTACGATAGCACGGTCAAGAACGGATGGAATGAGACGACCCACCATTACATAGGTCCGTCCCCACTCGACAGGAATATATAACCACTGACACTACTCTATTGCCCTTTGTCACCGGCCAGATGAATAACGACTGATTCGGCCAGAAGTCAGTTGGAGAGACGTTCAAACGAATGGTTACGACAAGTTACGATAGTCTCGTTCAAAGTTACGACAGTCGTTACGATAGAATTCGGATAATAGCGGAACTGAACGGCATAGTTTGGACAGACTCGGGTTGTCGGTTACGACAGAAGCCGACTAGTATAGACAGGGGGGACCCCGTCGGAACTGGGTATCGTCCAAGTGAACAGACGACGCAGACGTGTTTGGACTCTGAAGTGATTGTGAGTCAGCGAGGAACAACGAATATGCGATGAGATCGACCTTGGTATAGTCCAGTTACCAACTGGAGTCCACATTGTAGGGGCCTCGACAGCAATTGCACATCGGCAGGGACTATTGGCCGGACTCACATCCGACAGGGAAGATCGGTGTCATGCTATGAGACGCTAATCTCGACTTTTTAGCAGAATTGCGTACTGAGTCACAGAATCACGCAAACTTTAATCCATGCGCCCACTCAACTAGAAAATGAGGACGCGGACTTGGCCCGCTGGCTTAAACCCTCGGGACTCGTCCGGCTAGGGACGTAAGGTAATGGTTTACGTCCTGTTGACGTCCGTACTATGGTGAAATTGATCTCAAGAGGTTCTCCTGTACCTTTTCAGACTCAAGCATCCCAATCGTATTCTGCCACTGGGACGAGACATCTCGAGAATCATCGTCAGGCGTACGGCCCTCCACCCACGCAGCGATTCGGGTGCGCTCTTTGTCACCTTGACACCCGCCTTCAGGTGGCGGAGGATGTCAGCGTTTGACGATTGGTTGACGACCTGAACGCTTCTCATCTCACGCTCCCGGAATACGTCGTAACTCCACTACGAACGCCCCATCTCTCGATGCTATTCGTGGGATTTGCAGGCTGAAAATACTCCTGTGGGCCGGTAGCGGTGTTGATTCTACGTGGGATCAAGTGGCCAATGGGACGGAAGCCTTCGTGCCTAGTACTTAACCAACAGTTACGACTCTGGCTCACTACTGTTGGTTAACTCTGGCCGCTCTTATGTAGAACTG-59229

**7. *Halogeometricum borinquense* DSM 11551** (CP001690-95)

**Chromosme (2)**

1. **Hbor_02110**

190000-ACGAGACAGTCGGCATTGACACGATATGGGGAGAATCCTGACAAAGACGTTGTTTCCACGTCTTATGGTGATCACCACGACATATGGTAACAAGAGGCTAGATTAATAAGTATCATCGGTTCTGGCGCTTCTCTTCTGTCGAGAGTGTGTCTCCCTTTCAGTTGAGCTTGTTCTTCAGTTCGAAGTGAAACGTCCCCATCCCCGGTGTTTCGGGTCGAACTGATGTGAGAGTAGGGTCGTGAAAATAGCCGATTCGAACCGTCATCGTAACGACTACCAATGTATATCCGAATGGAACTGTCGTTCGAAGTGAAATTGGGGGTAGTGTCGGCGGAACACCCCCACCCCTTCGTTTCGGGTGGAACTCGAAAAGACCACCCCCGGTGGCTCGTGGAGAACACTAGTGGCGCATTAATACAAAGAGATAGTTGTTGCAAAATACTATTCTTCTCTACTACGGGTCCTGTTGAACGGTATCCATGATAAAACCCTCGCTTCGAAATAGTCGGTTCGAGGCCCCCCCACCCCTGTTTTCACGTCTCGACCTGAAACGAAGGGGTGGGGGAGGTCACCCACCCCACCGTTTCGAGTCCATAGCCAATCCCGACCACCAAAAAGCCCAATCTCAGCTAGTACAACCCATTATACTCAGTGATCTAGACCCGAAACACTCCCCTTGAAATCCCCTCGACCCCTCCTTTCAACTCGAGCAACCACTCCCCAATTATTTCTACACATTAGAATAAATCGAAATAATAACAATAACAATAACAATAACAATAACAATAACAATAACAATAACAATAACAATAATTAATCCACAGTGTTGTCCTTACGTTGTCCGCTCGCTTGCCCTCGACGGAACACGATAGGAACTGATAGACAAGTTTTTTGTAGCACCTGTTCGTTTGGTTCGTCTGCATCATCTGGACGCTCCACTGTCGTGCCTCTCGTGGAATACCGCAGTCTCATGCGGTGTTTCGACGGTTAAGGGCCACTGACGGAGTGTATTCCAGTCGAAACAGGGGGGATTCACAT-191037-**Hbor_02110 (Plus strand, 191038-192768)**

1. **Hbor_14700**

**Hbor_14700 (Minus strand, 1381922-1383127)**-1383128-AGTACAGGACACCGTGTTTCCGGTGAAATGG

CTTGCCATCTTCGGGCGCGACACACACACCACGTTTCCGGTGAACGCCCAAATTGCTGGGGGTTCGTCCAGATGAAACGGGGGTTTCGATTCACCGGAAACGTGGTGTCCCCGATTACCACTGTGCTGAACAACGCGAAAGGAGCCGCCGATTAGAGGCGATCAGTTATCAAAGCGTACGCTGGGAAACAGCGGAGAAGGACGTGATATGTACGAGAATAGCAATAATACGGAGAATCAGGAACTCGTGCTTGTCCCGGGTTTCTCGACAGAAAACGACTGTCCCAGTTCTCTTGAATGGATATTACGGATCGATTTGAACTCAGAAACGACATCCATCCTAGGTCGGGTTTCAAGAGTCGCTTCGCTTGCAACAAGTTCGAGTCTCGAACTTTTTCTTGTTTACGAGTGTATCAATGGACTTTAACACTAAACAACTATTTAATAGTTGTTATATGCTGGATGAGAAGCACCCATACCGTGATCTAGGTGGGGAGATGAGACGAGAACTAGGCAGAGAGGCGAAACGAACGAGAGACGACGGACCACCGACCGACTTTTTCGACTGTTTTGCCGTCGATTCCGACCCGATAAGGAACGGTCTATTCACCGGAAACACGGTGTGGTCGTTATTCACACGGCAATACGTATCCGTACAGTACTCCTGTCTTACTGCCTGATATACGGCGAATTCATCGGAAACAGGGTGTCTTCGGAACACGGCCATTTAACAATCGAGGAAAATCCCTCTATGCCGCCGAAATCTCGAACAGTCTCAAGTGATTCCGGACTAGGTTGTCGCCTCACTGGGGAACCATCGGCCGTCAGAGCACCCCACCCTCACCACACACCGATTCACCGGAAACCCGGTGTGTCTCTGTCTTATCCGATCCACGTGCTGTTCGTGAAATCTCACCGGAAACACGGTGTGTTTCCCCGGTGCACAGTCCCACCCGTCACTGCCGTCACCGGGGTGAAAAGCCGGGGCGACGTTTCACCGGAAACGAGGTGTCTGTTCGTCTACATTCGACACGAGGGTCTACTTGATGTAGACCCCCGGCCGGTGCCGTCAAACTGGGACCGCCGCTCGTTCGACACCCCCAGGTACAGATTCATTCACCGGAAACACGGTGTCTCTACCGGATATCCGCCGACGTGCGGTCAGCAGTTGGTACCTACGACATCACTCAGCACCTACGACATCACTCATTGCGTCGCACCTGCTACGCTATTTGCAATCTCTTAATCCAGAACGTCATCACCACGACACTCACCCACATATCGTGTTATCGAAGATACAATATCTCCAAACAAAGTCACTACAGACCGAAACAAAACGACGATTTTATCCGGATGATCTGTCGTCTCCGTCAGTTCGATAAGGCCTCCGCTCGTATAGTAAGTGATGACGTCCTCGGTGTTCCGCTGGGCACGGCACGGGGTCTGTCGAATCGGTGCGTGGGGTGCTGCTCCGTTCCGGTGGGTTGCTCACGCC-1384652

**pHBOR01 (1)**

1. **Hbor_31040**

**Hbor_31040 (Minus strand, 145499-146695)**-146695-CCTGTACCGCCTTCTCACCGCCAACTTACATAA

ATCCCTAGCGTCGTGTGTTTCGTCTGTTTCGTAGTACTCCTGTGTTTCACGAGCTAGGTAGTATATATTACACCCCCACCCCTCTTTTCGTGAGTAAATGGGATAGAAGGGGTGGGTAGAGTACCAAAAAATCTATTTGCCGGTACCAAAAGATTCAAATTATATGTATAGTTCTATTTCTAGAACTTGTTCTAGGGCTAGTTAGGGCTAGTTTTAGAGTAATTCACTGGGATACAGCTTCACTAGGGATACAGTCCGAACAACTCAAACTAGATAGACGGATATAATCTAGTTGTTCCGAGAAGTTGGAAGGGTTGGTAACATCCTCTTACGACGGCAGCCGTGAGTTGTCTTCTCCTGTAGACTGGATAATTCACGGTGAGTCCCGTGGTGATAATATCTGAGTTACGACAGGCACTATGGGGAATGTGAACAGACGAAACGAGGGGTGTCCAGGCCCCGGTACGGCACCACCCCTTCGTTGGATCTAACAGACGAAATGAGGGGTGTGGGTCCTAGTGCGATGTGTTCCATCCCTTCCGTTTGAGCTAACAGACGAAATGAGGGGGGTGTGGGGTACAACTCGTGTTGCCCCTTACTGGTGAGTTAACAGACGAAAAGAGGGGTGGTGCCCGAATGTGCTACGATAACAGACGAAATGTGGGGGGCCGACCGCTATCAGAATCGAGTATCACATCAGAATTATCACTCAGATTTAACTAACACTCACTAGCTTTCTCATCAGCTAGCCGGTCCGTTGACGTTCTCTTCATCCACTTCGAGAGGGGGAGTCACGCCGGTTCATTCTCTGTGTCTGCATGTGGTATCACAGAACGGCTGAACGACTCTCCCGACAACAATCGATCATAATTAATTAACAACCTCAGGGTGTAGATAGATA-147629

**pHBOR02 (1)**

1. **Hbor_34470**

168357-CTAACAGGTGTGACTGAGGACTTGAGTGAACACCCACACCCCCGATTTCCGTCGTTCTGTACGAGGATAGACTGCCAGACGTAGCGGTGGGACAGGTTAAATGGTCGTTATGTTGGGGGCCCCCGTGTTTCCGTCGTTCTTGAGAAAGGAGATACTTGGAGACACACCCACCCCACGTTTCCGTCGTTTCTCATTACGAGGGGGGAGGGGTGTAACGGAGCTACAAGCTAGCTCCTTTCTTATAATTCAGCTAATGGGCTGTTTACCCATATTAGATATAAATCTTTCTATTATTGTGGTTGTTTGAAGTAGTCGTGCCGTCTGCAAGCACTATCTTCACCTTGATTCATGTTACCGTTTCTCAATTGCCACTTGTCTGATGCAGCTCTTCATGAACAGTTCCTCGTGGATCAGATTGGAGGTAACACTATCGCGGAAGAACGACGGAAACGTGGGGTGGGTGTGTTCAGGAGGTTCTCTATGTATCCCGAGTGTCTTGCCTTCTGATCAGCAAAGAACGACGGAAATGCGGGGTGGGTGGGTCGAATAAGACCGAGGACATCCACTACTCCGAGTTGAGAGTGTGGGAGGTTAGGTCGCGGGAGAAGTAGCGAATCGGAGAGAACGACGGAAATGTGGGGTGTCAGACGGGGGATTCGATTGGAAAACACTGTTGAGTGAATGGTACGGGTCTGATTGGAACGAGTGAGAAACATGAGTGAGAAACAGATGAGTCCGACTGAGAAATATCAATTCTACCTCAATAACTATCTCAATAATTTAGTTCCCTCCTCTTGTTACTCACAACGACGGAAACGTGGGGTGGGTGTGTTGAAACGTCGGAAATGCGGTTAACCTATCATTGAAAATGAGAAGCCTGAATACGGCACTTTGAGTGTTTTGAGATCGGATATAACGTCGGAAATCCGGGGGTGAATCGAAGGTGGTTTTAAGTTCTATGAGTCTCACCATCCCCT-169334-**Hbor_34470 (Plus strand, 169335-170594)**

**pHBOR03 (2)**

1. **Hbor_39380**

**Hbor_39380 (Minus strand, 143718-144968)-**144969-CCGTATCCACTTTCTGTATATGCGATTAGATAAA

GATAGGGGACCAGTTCCGCGGGTGTAAAACGAGGGTTTCAGGAACTAATTATCCGAGAATACACAGATACAGTGAGTTTAGACGAGGGGACTACCGTCGCGTGTGTAAGAACCAGTATCAGAGACCACCTTCACGTCTGTAACTCAGATAGAGGGTAGCTCAAGAGACCGTCGTGTCTGTAAATGAGGGGGTACCGTCGCGTTTGTAAGTAGGGTACGGTCGTGTTTGTAACAACACGGACACCACCCTCGCGTCTGTAAACGACACAGAACCCATCAGGCCCACTCATCAGATGGTTTTGGGATAGTTGTCAGTGGATAGTTGTCAGTGTAGCAGAACTCACGAATTGAATGGTTTTCATCATCACACACACCACCCTCGCGTTTGTAACGCAAAGAGTGGGGGGTGGGGGGTGCGGAATTGGCCTGTCAAACGGCTTCCTCTGCCAGATTTACAAACGCGAAGGTGGTGTGTTGATTTCTCCCCACTACCCAATAGTAGTTACAAACGCGAGGGTGGTGTGTGTACGCCACAGCCTTCTCCGTTTAGTACCCCTGCGAGAGATAGTGTTCTCACATATCTTACTTGTCACTAACAAATCCTAGTTAACAACTACTAGTTAACTTACTAGAAATCAATTCACAAAAAGAGTAAGTGAAGACTTACAGACGCTACTATCGATGCTACAATCCGCCTGTTTGAA-145705

1. **Hbor_39520**

**Hbor_39520 (Minus strand, 158382-159620)**-159621-ATTCCCGATGACGTACCCTAGATTTGATATACGC

TTACCGTCCGGTGAAAGTGATTATGAGAACTGTCGGAAGGGTGAATCTGTCTAAAGCCAGCAGAAGTCTGTCTGTCTGTCTGTCTACATCGTGTCTGGGAAATGTAGTCAACAAATTTATGATATGTCGGACACAGTAACATATGATATGAGTGAAACTGAAGCTCCAGAAGAGCGTATCGAGGAAACCACTACCTGGCCGGACCTAGCTGTCAGCCTTTACGACCGGCTTACGGGCCGTGGTGCCGAGATAATCTACGAGTTCGAGGATATGCATGTTGACGTGCCGAGCGGTACCGGCGAGGACGCCGAACACGCTCACTGGCGACTCAACGGCACTGTCCGCGTGACGACCCGTGAGCAAGACTGATCTCGTCGCACCACTGTCGGTTGCCACTGATGACCTGACCCTTACCGTCGCAGGGGTTACGATGCCGGTTCGCTCAACCGGTGACCGGTTGTTCGTCGAAGTGCCGACGCTTCGGAGCGCGGTTCGGGTCGCTCGTGCAGTCAATGCCGTCCCGAGCGGACCCACACGTCTACTAACGGTAACTGACCTGACGACTGAACTCAGGGTCCGTGGGAGGACCGTCGCGGTCTTTGGTGCTCACACCCGGCCGGGACCGCTTGCCCGCGAACTTGGAATCGCCCCGGCCGAGTTCAGACTTGGAGGCGCGTTGGCCGCCGCTAGCAGCGGTGCGACTGCCGCCGTAGATCACCTTCTCACATGGCTTCGCTGACCTGAGACGTAAGTGGGGTTCTGGTGTGATGATCCGCTTCTCTTCAGGTAACGGCGTCTGCCGCTCGTCGGACACCACCATTTCCAGTAATACAGTTATCTGAGCAGTATTTTCGGTTGTAATTTCTCACCTCGCCACCCCCTCCCCACTATTTCCAGTAAAGCGGGAACAGAAAGCCACCCTAGCTATCACTACGGTCCGTTTCGGCGTTTATTCTTGCCGGATAACCTGGACGGTTCCACGTAAACTTCACATCAACTCCAGATGACTCTCCGTACAACCTCTCATCTAGCTAGCCTACGGACCAACTTGTCTGACTACCATATGAATATTTCTAATTTCAGTATGTGTTCTCAGCCAACGGGCTGTATTTGTGGTGGTTTACTGGAAATAGTGGGGAGGGGGTGTTGCACTATCCTTATTTACCTCAGCCCTTCGCAGTCTACCGATTTCGTTCTCACACTATTTTATTGGAAACAGTGGTGTTCGACTATCTTATAAAGAATGCTTTACTGGAAATACTGGAACACCTCGGTCTGCCTTCATCCGGGCTGCTTTCGTTCAGGCTGTCTTCATTCGGACTTCCTCCATCGTGATAGACACACGCCGGCAACCGACGTTTACTGGAAATAGTGGGGTGTTCCTCCCTCGTGTGTCCCGAATGTTTACTGGAAATGATGGGGTCTCACACGAGTAGTAGAAACATCACCCATTGGTGGTTATACTGGAAATACCGGAAACGTCGGTCGAAACAATTTTCCTCGCAGACTGAATCAGTCGAGTACAAT-161181

**pHBOR04 (1)**

1. **Hbor_36740**

**Hbor_36740 (Minus strand, 77814-79067)**-79068-GAACACGTCCACTCATCGGTGGCTTATAAAACCACC

GCGTCCGGAGTGTCCGCTGTTCTCGAAGTATATAAAGAATGGCGAACACACACCCCCTCCACTTTGTCCGGAGTTCTAGTGAAAAGAGGAGGTGGGGGTCGTGACGAGCAGCAGTAACCCTTAGTAAACCGACGGTTTTATTGTGTAGCCAACCAAGTTACTCCGTAGTCCTCTTTGAAAGAGATAATGAGTGAGGGAACGAACGAATATTCTCTTTCTAGTGTGTATAAACCGCTAAACCGGCAACTCTGGTGCGCGTAATCATCCGACATCTCATTCGGTCACCTATCCCCCTCACCCCTCCACAAGACGAGAACAGCGGACAAAGTGGAGGGGGTGTGTTGCGACAGTTGGAACGACACGGAGCTACGTTGAAACCGACTTAGAACCGTGCTGGACCCGGCTACGAACGTGTTTGGGACACAGAGTGCAGTAAAATCGCCTTAGCGAGGAGCGTGACAAGCTATCGACTGGATCGTTCGTCCCATTACACTGTTTTGATGACGATGCCGATTCACCGAATCCCAAGAACAGCGGACATGGTGGTGTGTTCGAGAGACGATTGACGTTCGAGCTACGACTCAAATTCGAACTCGAGATTGATCTGTATTCGAATCGCATCTGAAACTTGTCGATAACTCCGGACAAAGTGGGGTTCGAGACGACTTTTATTCTCTGACAAATAGCATTATTTTGCGAATTTAAACACGTTCTATAGGGTTATTACTCCGGACGCGATGGTTTCGCTTTGAGAAACGAAGTGACTGCTACTTGGGTGTCGAAATAAGCTCTGCCAACTCAAATAAACTGTGCTAACTCAGTATGATGGCTCGAGATGCAATCTCTGGTTCGACCAAACCGACGCTATACTGACCGAACTGACGTCACAGTTCGTCCGGGTACTCTCTCAGTTGTAGAACAGCGGACAAAGTGGAGGGGGTGTGTTCGACAGTACGTTCGAGAATAGCGGTATCAATGGGGTAACACAGCTGAGTTTTCGTCACGATCTCCAAAATGCCGCGGTCATTCTCCTGCGCACGCGAGCGAAGAAGATAATATACTGACCTAGTCTATCACCGACACAGTCTATCATTGACTCTCAACGAACACCACTCCGTCCGCTGTTCTTTGTGAGCTCTCGACCGTTGTTACCGTTCGAGAGACGGAAGTGCTTAGAGTTAAGTTTCGCGCCCGAGTGATTTAGAT-80309

**8. *Halomicrobium mukohataei* DSM 12286** (CP001688-89)

**Chromosome (1)**

1. **Hmuk_0815**

783883-CCTCGTTCCTCCCGCCCGAACGTCCGTATTCGGGGGCTTGAGCGTAGTAGAAAGGCGAATTCACTTAAGGCTACGTCAGACAACCATACCCGAATTGATAGTACGTCGAAAGATACAGCGCGGAAACCTGTCGAGAAAACGCGACGGGGACGAGATAGGAAAGTTGCGGGACGATAAGTCAGCCTGGCGGGACGGTAAGCCAACCCGAGGGAGGAGTGGAGAACGGTAACAATCTGGGAGGGACTTCGAGGGGGAGCACAACACCCCCACCCCTTCGTTTCGACTGGAACGCCATAACCCCTCGTGGGGACCAGCGGGAGAGACGGGACTCGCGCGGGAAACCTACGGGTGGTTCGCGCGGGAGGTTTCGTCTGGAACGCTACTAGAGAGACGTGAGAACGTGTAGAACAGCGTCTAGAATACTAGATACGACTAATTACTTGTCTCTGCTTCTAGTGCGGCTTTGGTTTTCACGTATCATACATAAGTCTTCCCTCATCTAGTGAGACCTCGACAGACTGCCGTACCCCCCACCACCCTCTTCAACGCGTTCCACCCGAAACGAAGGGGTGGGGGGGTTCGTGAACTCCCTTCCGAAATCCCACTCGAAGCTGTTCGTCTCCTGCTAGCACCTCTCTCACGTCGACTCTCGAATACACCCGACAGTACAGACCCCCGTCCGTTTCTGGTCCGCCCGCTCCCGCCCGCCGACCGTTCTCACTGTCGACACTCTCCTCACTGTCGAGTGTGAATTCGTCACGAACGTCTCCGTCTCGGGGGAATGCCTGCAGGACCGGACAGCTATCCACATCTCGAAGGTACGGTGAAGCTGCGGTCAGTAACGACAGAGACACGAATCGGGGACCCGACGTAGGGCTGGATCGACAGGAGTCTCCGTTCCGACCGAAATGTTTAATATCGAGTCACCCACGGGTTCTTGTGGTTCAACTGGACTCGCGCCGTTCGGTGAAATTCGCGTACAACGTCTTTCCGAGCGGTCTCAGTCGCGATTCCACACTGCGCACCGGTCTTTCCACCTGAAACGAGGGGGTTGTCACCACGACT-784948-**Hmuk_0815 (Plus strand, 784949-786499)**

**pHmuk01 (1)**

**2) Hmuk_3249** (39319-40662)

**Hmuk_3249 (Minus strand, 39319-40662)-**40663-GATACCGTGATATAATCCACATCGCAAATAAAGATT

TGGAACCGCCTGCGAGATTAAATGTAGAGAATATACCGCATAGAGTCGATAATATCTGCGAAGAGATGGAGATGGGACGTACCGTGGGCAAAGTCAAATACCGACGTGTGCAGAGACGGACGAGTGTAGAGAAGGGGGTGTGTAGAGAAGGAGATGTGCAGAGAAGGAGATGAGAGGACACGGACCCCGTGTGCGAGATGAAACGAGAATCCGGAAACCACGACGACTGTGAAGATCGAGGGAACACCGTTTCAGCGGGTTCGGATTGCGTCCCGATTCGGACGACGTGCGGACCCACACACCCCGTGTGCGAAATGAAATGCAGACGAACGGAAAGGTACTGGTAGAGAGTCGAGTTCCGACGGCGGTCGGGATCCGTCGCCCCCACACCCCGTGTGCGAAGTGAATCGCCGTGGAGAACAGAATGGGGAAGTTGACCCCCACACCCCGTGTGCGAGATGAAATCGGCAAAGAAGGAGAGTCCCCTTCCAGCCGGAGTCAGTGACGGATATCGTCGCAGTGGTCGAGGAGCCGCGTTAGAGGTCTATAAAGACGTATCTAGGGATGGCTGACGATCGATAGGTTACTGCAGTCGTTTTAGATCGTCGGTATATGTTATGTGTATTTTATCTAAGCTATCTAGACTATCTAGCATATTTTCATTCTCTTTCTCAGTTCTTCTTGCAGTACGGCTTTGGTTTTTCCTTCTTAGTAATAAGTATTCCTATTGTACTAGCTTATTTTCTATTTTGGTCTTTCAGACTCTCTCTTTGCTGAAAACATCGATATTGGCTGCTTCCTGTCTCAGCTCGCCCGCTCTCCCTAGCACCCCACCCCCACTCTCTCACCGATTTCATATCGCACACGGGGTGAGGGGGTTCACCCACTCACT-41590

**9. *Halopiger xanaduensis*** (CP002839-42)

**Chromosome (5)**

1. **Halxa_1419**

729722-CTGTAGGGCCCTCCAGCACCGCGGGGTGCATCTGAGCGTACTACTGACTGAATTCACTTAAGCTTATGTCAGACATATCTATAAACCAACAACAGTGAGTTATCAGGAGGGATGTGAAAGTATGTCTCTCATTAATCCGATATGTAGGGTGGTCCATGGCAACGATTGGCTCGATCCGGCCGTGGTCCCCCACCCCTTTGTTTCAACTGGAGAGTGCCGAGGGTGCGGTGTCCCGGAGCAGTTCGATCGGTCAATCGGACCCCTTTCCTTCGAGTCGAGCCGAAAAGAGAGGGTCTCTCCGGACGGAATCGGGCGGACCCAGCGGTTCTAGTAACTCGAGGTGCTCTAGATCGCGAATCTAGTTGAAACTAGTAACTAGCTGCAACAGCCCTAGTAAAGTCCTAGATTTGCTAGTCGTTCGGATATTGTTTTTCAGGCAGTCACCTAAAGCTTCTCCTTCTAGAGACACCTTCTCCCCACCCACCCCTTCTTCAGGCCGTTCCACCTGAAACGAAGGGGTGGGGGGCTTCCGATGATCATCGTCGATCTTCCAAACTGATCACGAGTCACGATATCGAGGCCCACTATTCATCAGAATCCTGTTCTATCTTTCCAGACACTGTTCGACGATATCATATCGTCCGGTCTACCGGCTTCCAGAACGATGTGCCAGGGCGTTCGCCGACCGCCTAATCCATCATTAGGTCACACGGGCATAGCAGCAGGGTACCCTAATCCCGTACTGCAGCGTTGCAAGCGAGTCCAGTCTCGATTACCCCCGTGAATCGAAGGGAAGAATTTAATACAACGCTCTTCCTCTGTTTCGTTTGCATCAACTGGACCCGGACCGGGTTGCTGGAATCGTTGATGGGCCCCTACGGCCCGTCTACCTCGCTACATTCCTGATCTCGGGTTCGGATTTCCACCTGAAACAAGGGGGTGACTGTACGACGG-730675-**Halxa_1419 (Plus strand, 730676-732370)**

1. **Halxa_2024**

1344933-GGATCGTTTTCCCTTCGACTAACTCCTCTAGTTGCGGTTTCACAGCAAGTCGTCGCTAGTTGCGGTTTCACAGCGAGTCGTCGCTAGTTGCGGTTTCACAGCGCGAAATCCCGGTCGGACCGCCGGAGACACACCCCTCGTTGCCGCTGTATAGCCCTTCGAGAACGGTTGTAACGCCCATTTTCCGGAAGTATCCCAGATAGTAATCTCTCGAGCGAGGCACTGCGCCCGCGACGCGATCTCGCGAATGGCTCCGTTCCGGTCTCAGTTTCGGTATTAATAAATATGAGATCTTCGTGATAGCCCACTAAGTGAGTTGAACGTCGTGACGTTCAGTCAAAATTGAACGTCCGCGACCGCTCGAGTCGACTCGAGGCGCTGTTTTGACTCGGTTTTTGGCCCGTCGGGGCCGCCACCGACATGCTGGACTCGACCGACCCCGATTTCGAGTCGACGGAACGGTCGATTCCTTACAGCGGCAACGCGGGGTGTGTGTCTCCGTATACAGCGGCACCGAGGGGTGGAAAACGAACCGCTCGCTTCCTATAGAAATCCTTTTCAATCAATACAGCGGAAATACGGTGTCCGTCCCGCTCCTGCCGGTTCGTGTGTGGGACGGAATCCATCGCAGACCGGGCCGTGATACAGCGGCACTCCGGGGTGGTCGGTTGCTATACAGCGGCACTCCGGGGTCTCGCCGCAACGCCGTGCCGTAATCGCGGGTTGTCCGTGACGGGTGCCGACCCGATCGCCTATACAGCGGAAATCCGGGGTCTGCTTCGGCCTATACAGCGGAAACGAGGGGTGTAGATAGGGCGACTTAGTTCTTGAATCCCTGAAATCTCGAGTATACAGCGGAACTCCGGTTCCGAACGTTTAAGAGAGCGCCGACGAATAGCTCGCATAACTGA-1345843-**Halxa_2024 (Plus strand, 1345844-1347043)**

1. **Halxa_2660**

**Halxa_2660 (Minus strand, 1986154-1987440)-**1987441-GTCCGCATTCTCGAAGACGTGTATTATAAAAC

CACGTGAAGGCGTTCAGACTGATAACGTTCAAGTATGGCGACGAATACGCGGTAAATCGCTCGGTCGACGGTTCTCGAGTTCAACGCCCCGTGCAGACTGAAATCGCACTCTGCCTGCGGGTTCAGACCGGATTCGTCATACCTACCCACACCCCTCGTTCAGAGTGAAATCGGTCGAACGGGTGGGCGGACTCGAGTAGGTGTGGTGTCGGGGTAGATCACAGGTCCACACCGACGGAAGAGACTCGTCGAACGGACTCGACGATATTCGACTCCTCCTAGAACAGACTTGAAACCGCCACAACCGCCGTTTAGCCATCCGATTTCTCGTCCTCTCTTCTCTTCTCTCTTCACCAACCTAGCTAGTAGTATTGTATCTTACGAATGAAGAAATATTATCTTGATCCTATCCGAACTCGAGAACGATCGGACCGGTTCGGACGAGATCCCAGCGTAGCCGGCTACCGGCTCCCCCTTCGATCGGCCTTTCACTCTGAACGAGGGGTGGGGCCCTCCGCAGTCACTAACGGGCCGCCCTCGAGGGACTGAGCCAGTCGTTCCGATACGGTTCGCGTTTCGTCCGGACACCACCCCTCGTTCAGAGTGAATTCCGCCGCGCTATCTCATCCTCCGAATCCGGTGCATCTCCGCCGAATTGCGCCCGATACACCGATTTTCCTGTTCACTCTGAACGAGGGGACTGACTCTATCCACGACGGCCGTCGCTCTCGGGCCCGATCGGTAGACGGTTCGAACGGATTTGCCCCTTACACACTCTCGACGACACCGTTTCACTCTGAACGAGGGGTGTCCCTGAGCGAACCGTCCAGCAGCTCGAGGCGAACCCTACTTTCACGGTCGTCGCCCGCGTGTGAGTGGAC-1988358

1. **Halxa_3357**

2693745-GATTAGCTCTCGTCGTCCGATTTGGTGGGCGATTGCTCGAGCAACTGTCTGCGAGAGGGCGGCCCGTGAAGCCGAGCAGATGAACCGATCATACGGGAGGTATCGAGCGGGTGTGGAAAAGTGATGCGTTCGTGACGGCGGACGGAGTTAGGGTTATGGGACCTCGAGGCAGTTGTGATGTTTGTCGGGTTTACGGATCGGACCGATTTGTTCTTCCTTTCTCTTTCCTTCTCTGTCTCGTTGGACTAGTCTAGTATCTAGTTCTCTAGTACAGTTATTCACCTGCACCGAAGTCTATTACACAAATCGGAGTTGGGATCGCATAGAATACTCGATCTGTCCTCATTCTCTGGCAGAAATCTCGAGGATTGCAATCTGATAGGGTGGGGAGGGACACACCCCCCTCGCGTTTGTAAGCCCTTGTCGGTGGGTGAGGGTTCTGTACGGGAACACACCTTATTCGCGGATGTAAAATATCGCGTTAGACGGGCCGATTCGTGAGAAAATCGGCGATTATCTCACTCCCTACCCACCTATTTCCCGTTACATCCGCGACGATGGTGTGTGTGCCGAACCCGGAATGTGGGTGCCGATCCGAAAATCGCGTTTCCGTAGTTTTTGACTCGAGTCGATTCGGATTCTTCCTCGCAGTTCAGTTGCTCGCCGAACCTCGAACTCGAATTCCGATCGCAATCCGGCGGAGAGGCGACGTTCGGCAGTTACATCCGCGACGGGGGTGTCCGTCCGTGAACGGCGGGACAGCCGCCCGATTAGACGCGCGATGGCGGCGATTCGTATCGATACCGTCGTTCGTCGGGTATTGTCGCCACCTAGATGCTAACACAGGCGATTTACATCCGCGATCGATCTCCATAGCTTTATTTCAATCCAGCCGGTAGTCGCCCGACACTGCA-2694658-**Halxa_3357 (Plus strand, 2694659-2695930)**

1. **Halxa_3564***

2889238-GGTACCTCGAGTGCGAGTTCGCTTTTCGATCCGTCGCCGGGATTCTCGCAGCCGATCGTCGCTCGAGCGCGACGCCCTCGGAGAGAGACACACCACCGTTGCAAGTGAAACTGGAAAGCGCGGGGAGGGGGACGGGGTGGTGAGCGGTAGTGTCGGCAGGTCGTCGATCACACGAAACCAACAAATTCGGTTTAGCAGACTACTTGGCGCCGTGTATCCGGGATACGGACGGATCGCAATTATTGTCAGCGCTTTAGATGAAACCGTGGACTGTTCTCTCTAGCAGTACCTAGAATACTAGTTTCACTTGAAACAGTGGTGTGTCTGAGTTCACTTGCATCAGATGAAAAAGTGGTTTTAAATAGTACATCTCTCTTGGTAGGGGT-2889623-**Halxa_3564 (Plus strand, 2889624-2890850)**-2890851-GCCGCCCCCGCTTCACACACCACCTTTTCGAG

TGAACGGAACGTACCGTCCCGCTATCTTCCATTTTCACTTGCAACGGTGGTGTCTCCGTGGCGGCATCGATGGGCCGAATTCTCCCTGTCTCGAGTTCGGGTGCCCCGTCTCGAGGCCGAGCGACTCGCAAACGGACCGCGTTACCGCTCGCGGATTCGACTCGAGTTTCACGTGCATCGGTGGTGTGGGTCGTTCACATGAAGGAGTGGTGGGGACCGTTCACGCGCACTTGTGGTGTAGCTCACAATTCACTTGAAACGGTGGTGTGTACTCGCGAGCGGCGGTACAGGGCTGTTGCACAGCGATTCGACCCCTTCGTCCGTCGAACCAGTCTCGTCCCGCTAGTTACCACGTATTTTATACGAACTCCCGCACACGGGACGAGTATCAGCGACGG-2891280

**pHALXA01 (1)**

1. **Halxa_0635**

374773-GGATGTCCTGTATTCGGCGAGATCGTTCGGATATGACCTCTCTTCGAACAGCGAGGAAATCCCGCCGTTCACGGCGAGGAGACTGGTACCGGTATCCCTGATCTGGCATGGGAGAGGCCGACGGAGCGGCAACACCGTTCGGTTCCGAAGCGACGAGACACGATCGGAGTAGCGGTGCTGCGTGTCCTCGGCGATCCCGGGAATCCTAATCATTGGGAAACCTGGGAATCCCTCGAATTCAATGGAGAGTCACTCGAGACAAACGAGACAGTTGGAACGAGATACCATACACCAAACAGAGGCACACACCGCGTTTCCGGTGAAAGCCGCTGAAAGGGTCACGGGGTGGATTGATTTCCGTCTGTTAGTTCACATTATCTTCACGGATCTACTCCTGCCATACCATACTATTTATTATCATGTAATGAGTAAAGTCCATTGAATGGATCGCATTCAAATAGATAAGCGACGAAGTCGCAACATATGAACTCCTGAAATCGGGCTCTATTGTTGGGTTACGCTACTCCTCGCATTCGCACTGTCAGCTGTCGTAGCATCTTTCCAGTGAATTCGAACCCCCGTTTCTTTTGGACACCATCCCCGCTTCCGATGTGTTTCACCGGAAACGTGGTGTGTGTGTCTCGGCGAAACGTTGAAGAGATTTCACCGGAAACACGGTGTCCAAGTAA-375462-**Halxa_0635 (Plus strand, 375463-376677)**

**pHALXA02 (1)**

1. **Halxa_0082***

73252-AAGGACAGCTACTCTCGGACCCCCCGTGTTCGAAGTGTAAACTAGTAGCTGGCCGGCAAAGCGTAGAACCCGATGGTCGTTTTCACATCGATGTCGGGGGGAGGGACCACCCGATTACACTTCGATGGCGGGGTGTCGTCCGTACCGGTTACACTTCGACGAGGGGGAGAGAAATTGCTCGTGTCAATTATCATACGATCAAATCGCAGGAAGGCGCGGAAGTTCTCGTTACACATCGATGACGGGGAGACCCTCATTGGATATACTTCGGTGTCGGTTCCAACATGGGAATCAGAGATCAGCCGAATACGGAGTATAGATTGTCCAGACCGCATGGATAAACATCGAAGGAGGTGAATCTTTTTAACGCCTCGGCATACAGCAAGCGTATGGTCGGTAGTGATCAGGATGGGATGGACCAATCTACCCTCGAAGAAGGACCTCCTTCCAGAACTTCCTCGAGTGACGCAGAACACGTAGAAGCCGGTAGAGAAAATGGCGCAGAGGACGCGGTTTCCGGACAAGACCGAGGAGCGTCTAAAGGTAGTACTCAGCGATCGATTCGGGAT-73821-**Halxa_0082 (Plus strand, 73821-75110)-**75111-GACCACATCTTCGGAGGATTCTCTTGGAACACACCCCCCGTCATCGATGTGTAA

CTCGAGTCCGGTTAGGAAGAGTCTCAATCTCCATTAGTAGCAGAATCGATATTGAGTCTGTACTGGAGACTACCTCTGTTGCCTTGATGTAAAACTCTCTAAATATAGAACCCCCTCCCCCCGTCATCGAAGTGTAAACGGGCGATGTAGACCCATAGGGAAATATGAAGAGATGCAAATACCGATGAGAAAGTGGGATAAGAGTGCTAATTTCGCGGCGTTCAACGACTTCGAAGCCTGCCTAACCAATCACTCGGTTTGTCGAAGTGAACTACCCCATCCCTCGTGATTAGGTGTAGTACGGATGTGGTTCTCACTCATCCTACATAAAACTTCGTCAGACAATAGTCCATCTTAAGATAAAGTCGTATTCTGGTGATTGAGGACCTCTCCTGCGTATTTCCAGCAGTTCTTTCTGTTGCTGCTATTTTCTTGCTCTGATCCCTCTTCTTCCGCAGGGTTTTACACATCGATGACGGGGGGAGGGAGTGGGCCTTCTTCTTCTTTTGCTGTTTATTCGTCGCTGGGCGTAGTGAATGCTG-75707

**9. *Haloquadratum walsbyi* DSM 16790** (AM180088-89)

**Chromosome (2)**

1. **Cdc6_1**

CCTGTCCTCCCACGCCTCTTGAGTGGTACCAACTGTCCCGAAGTGGCGTATGGCGGTATCACGTTGTTGTGGTCACTTAAAACCGCGTCAGACATCAACATAGAATATGACATCCAAGAATTCCCCGCTCAAAATTATATCTAGCATCTGGCATCTTAGGTCTAGCCTTGATGAATATAGTGGATAGCACTATTGCACTTGTTTTCACGGGTAATTGATATTAACAAATCGGTTTCGATTGATAATATCCGTTTGATACTCGGTTATGCCGTGTGCCCCCCACCCCTCTGTTTCAGGTGGAGATGCTAAGAAGTGTGTGCGGTTACTCACTAGTCGAGATATTGTGACAATCTTCTAACTCTTTATACTTAATTACAACATAATATACGATGTACATAACTACTAAGTTGCAGTACGGGTATTGTTATATAGTATATTATATAAAGCTTTGCTCATTAATACTGTATATAATCGAACACCCACCCCCTTGTGATTACAGTTCCAGTTGAAACAAAGGGGTTGGGGGGTATTGTATTTGATACAGCGAACAGAAAATGCCACTATCATTCCTAAGTGATGATCCCGCTGACCGGGTGGATGGATACATCATATCGATTATCTGAGTATCTTAAGAATCTGTCAGACCAAACTTGCGTTCAATAGGTAGGTGTATCATTGTGACATATTCACTCGACTCTCGACTCTCGACTATGTTGGTTTGTTGCATCTTATACCTCACAGGGATGTTAGTATGGTCGTAACTACAAGAGATAGATTTTTGTATCGTGCACTCATCGATTTCCATTGCATCAATTGTATATCGGTCTAATTGATCAAAGCAATATATTGACCGTTTCAATACCTAAACCCGCCTCATTTGGACGCTGTCATTGGAATTTCCACCTGAAATCAAGGGGATGTAGACGGGTGGACGGGGGAATTACCAA-722-**Cdc6_1 (Plus strand, 723-2426)**

1. **HQ2959A (Cdc6_)** (2258609-2259895)

2258070-AAGCGAAACAAAGCAAACGCAAGGATAATTGCTATAACAACCCCCCAGAGTGTGAATGGGATCCGTCAACGACACCCCTCGGTGTGAATGGGTGAATAGATAGCGAATTAAGATAACCACACCCACACCCCTCAGTGTGAATGGTATACCACCGCTCATACAAGATTCAATCTCTCCTGTTCAATGTTGATACGGTCACAACTGGTGACCTTACTTCACTTCGCTCAGCGACCGGCTGCGGCGCAAGCGCCTTGCCTTGTAGAGATATTTTACTTGATTAGTTGTTTATGATAATGTATAATAAAACTACCTACTGGTGTGGTAGCGGTGGCGTTGCCAGTGCAGTTGAGCACACTGGACAGACAACACAGCAACAAATACCGCCCGATGGTACGGACATCCTTGGGTATAGACAGTCAAAAGCATGTTCGAGACAGTACTGCTGTGTGTTCTCTCTTCGATTAAACCGGTGGACGAGTGTTGAGTTAGCATTATTGTGTCCTATGGTGGACCATTCACACCGAGGGGTGTGGGAGGTA-2258608-**HQ2959A (Minus strand, 2258609-2259895)-**2259896TCACATAAAGGT

AACCGACCCTCGATGTGATAGGTCAATCCCTATTCACGTCTTAATACGATTGAGAGTACAATTCCGTATGTATATGAATTCTAGAAATTGAAGGATCCCATTCACACTGAGGGGGGGTCAAATCAGGAACCCCATTCACACTGAGGTGTGCTGGATGGCGATTAACAAAACAGAGCTTTTAGTTTAGAGAATATCATTGATATTCGATCTTATGAAGTACATTGTTGGTGGCGAGCAGTCGGGCGATTTTTTGTATTTCTCTACGGCACCTGAGCTTCCCAGAACGGGAGTGAGACAGCGTGAAAGAACCGATCTACCGAATTTACTCAGTTCGGAGAGTCATCACCAACAATCTGCTTCACAAGAGCGTTGATATTAGTCTCGGAACTTCTCACCGAGGAGATTTCGCTCGGCGGGATCGCCGTCATGTGCGAACGAGACGAATTCGTTCGAAATAATATCTGTGACAACCGGGAGTTTGAGCTTCTGGAAAACAAGCCCACCCCACTGGTTCCGCTGTTAGCAGATTGAAATGGGCGTACAACTAGACAGAAACTGTATCCTGATTATTACTGACGATAGTAATGATGTAGTCGGAATAATCTGTCCTAACATGAGTTCAGTACTTCCAACATACCCCACTGGTTCCGCTGTTACTGAAAAATAATTAATCAGCCAATAGAATCAACCGAGTATATCTGACAACTGAGCAGTTACAAACGATAGTGTAATTAACAGCGGAAGTGGTGGGGTGGGTATGTTCCACGATTAGACAGCAAAGACATCTGATATGTCGGTTCGGTTTATATGC-2260718

**10. *Halorhabdus utahensis* DSM 12940** (CP001687)

**Chromosome (2)**

1. **Huta_1077**

**Huta_1077 (Minus strand, 1053027-1054232)-**1054233-CAATATTGGACAATACAAAGTCATTATATTTT

AATCTATCGACCTCGTACCGAGTGTATACACAGATAAGACGAATACTCCATAAATCAAAGAGCATCAAATCCATTTATATTCAATATTTGTTGGTACACACACCAAGTAACGAGTGAAAGCACAGGAAAACTCACTCAGAGACAGAGTAAAATATCGCTGCCGACGCCACCGACGCTGGACCGGTTCGAGACACAGTGCTTCACTCGGTGCGAGGTGTGGGAGTACCGCTCCAGCCATACCGACGCTGCAGGCTTTCACTCGGTGTGAGGTGTGTCTCACCGCGCCGGCTCTTCCTCAATCATCACGGGAGTTCGACGACTGGCTTCCCGACCCTTCGCGTAATGTCCACTGGTCACACGAAGGTAATCTCAGGGCCTTCCTCAGTAACTCGACCGAGGATGTCGTTGCAGTATTTGGCTTCTCGCCGAGAGAGTGATTTACTCAGTCTGTAACTTGGATCTGTTCGTCCGAATTTCCATGTGTTGATATTTGTATCATCGTGACTTGTGCTGGATCACCAGTCACCTCGGCATACTCTTGTGGCGAGATCCGGAAGACAGTATCTCGCTGACGGGCTTCACGAGCGAGGGCCTGGAACCGTGCACCGCCTCGCCGGCCGCAACTGATAAGAACTGACGAATCAATGAATACGACGTCTCGATTGGGGGCCATTCCGAT-1054944

1. **Huta_1613**

**Huta_1613 (Minus strand, 1593546-1595321)-**1595322-GTGGTTTTCATCCCCCTTCTTTCAGGTGGAGAT

TCGCCCGCAGATCCGAGATAGCGGCCGAAAACGGCCTGTAGAACCGATATGGCGGATTTCGAGATGGCGATCTTGGTGCAGTTGAACCACAAGAACCGGTGGGTGAAAGGCTATTAAAAATTTCGGTTCAGATCGTGCCCAGTCGATGATGATGGATTCGGAGAGGTGATCATGTCCGGTGAATCAGTTTGCAGAGGTCCAAGCACCGTCCGGATTGGAGTGGTCTGGTTTCCGGATTGGAGGGAGCCTGGTTTTACAGGCTTCGATGCGAATTCCCGGAGCTTGCCCCCGAGACGATCCACACATCTGCATGAAGTCTGGAGAGGTTTGTCATTGAGTGCTATTGTCAAAGACACCCTCCCCCACCCCTTCGTTTCGAGTGGAACAGTCCAAACACCCGGGGAGGGGGGTTCAGCAACCGGGTAAGGACAATCTAGAAAAGTCGGGTTTTATTAGAGAGTACATATAACAGTAACCGTAATGGGAACCAATTCTCTCATTGAATAAATTTAAAGTGTCTAGTAGATATTAGTACTAGATCTACCTAGAACAAAAGTACTAGTTTCCACTAGAGGTTCTGTATGGCACTCTCATCTCCATTGACGATGGTTCTCAGTCGGTACTAATCGGTGTTAGTTGGTCCTTCCTGAGACGATCCGCTGATATACTGTCGAACGGTTTCGGCGATCAGTTTGGCCTCACCCGATGCTTTGCCGTTCTGCTCTACAGTGTAAGATACCGAATCATCCAATATGCATCACGACTGCAGTAGAAACGATCCCGTGCATTCCGGAGACTGATAGTCTATTATATACTGGTCTATACCAGTCTGATGTCATGGTTTCGTTATATCTACCCATCGCCAATATCGAATCGCCACCGAGTCGGTCTGCGTGTGGATTTTCGCTCGTTCGGACGATCTGTACCGGTCAGCGCATGGTCCACCCGGTGGTTCACGGTGTTCCACTTGAAACGAAGGGGTGGGGGGT-1596343

**11. *Halorubrum lacusprofundi* ATCC 49239** (CP001365-67)

**Chromosome I (3)**

1. **Hlac_0001**

GTCGTCGCCGCCGACGTTTGGGGTTGGGCCCGGCATTAGCCGAAGTACTCCGCGATCTTCGTGTACACTTCGTCCATATTCTCGCCCTCCAGCGCGGACAGCGGGATCGTCTCGTGTTGTGGGAAGGCGTTCGCGATCTTCTGGACGTCCGACCCCGGGAGGTCCGTTTTGTTCGCGAGAATGAGCGCGGGGAGATCCTGCGACTCGATGATCCCGATGAGCATCGTGTTCACCTGCGTGAACGGGTCCGTCGTGGAGTCAAGCACGTAGATGACGCCGTCGACGTCCTCGCGGAGCCAGTGCATCGCCTCCGCGACGCCTTCGGTCGCCTCGCGCGACCGACGGACGGCGTCGTCTTTGTCCATGTCGTGATCGAGGAACTCCTTGTAGTCGACCTTCGTCGTCACGCCCGGCGTGTCGACGATGTCGATCGTGACCTTGCGCCCGTTGCGCTCGATCTCCACGTTCTCCTTCCGGCGCGCCCGGCGAGTTTCGTGTGGAATGTGGCTCTCCGGCCCGACGGCGTCACCCGTCCAGTCGCGTGCGATCCGATTCGCGAGGGTCGTTTTTCCGGCGTTCGGCGGTCCGTAGATCCCGATTCGCTTGGGTTCGTCGGCCGTGAACAGCCCGTCGGTGACCCGTGATATGCTGTCTTTGAGATTCGTGAGCAGTCCCATCCTAACCTCCAGTCCTGTGTTCTCTCCCAGATACACTGTGCGGTGTGGTAACGCCCGATGCTGCGCATCCAGCGTGTGTACGGGGAATTACGATAGAATCCACGCCCCTCTCACTTAAGAACTGCGTCAGACACGGGCGATTTGGTGATATTCGGGTCGTGCAGTTGTTAACACGCGGGATATCGGTTGTTTACTTATTCGAGCTTATTTATATATTCTTAATAATCTGGAATTGGATATGGTAGATCGACGAGAGACGGGCTAACGGGAAACAAATCTTATTCTATTGATCTATCCCGGTGATCCATCTCGGTGATCCATCCTGTTGATCTAGCTCGTTCTGATTCTCTCCCCTCCCCCACCCCTTTGTTTCGACTGGAGACCGTTATCGGTGGAGGGGTGGATACGAGTGGTCGTGTCTCGGATGTAAATACGAGTGTTCAAACGATTTACCGTAACTACTACTAACAACAAAATAAATGATCGGGCTTTGTCGAGGGTACTGTTTTACAGACATTATAATAAAACTACCGCTATACTATATCCTCTTCTCACCCCCCTCCCCCACCAAATCCGAGTTCCACACGAAACGAAGGGGTGGGGGGTCAGGAGAAGACAAACAGATAATCCGAGTGGATAACCGGCACCGACTAGACGATCACGAGACTGAAACGGGGTGGAATCCGAGGACAGCTCCGAAAAACGAGTGGATCGCATTACGGCTGTCTGGACTCGAATCGAGACAATACACCCACGTTTCCCGTCCATCGAGTTTCCCCTCCTAACGTCTCGATCTCGCTGTCGATTTTGTCACTGATCTTGACCGACACGGTCCGTTTCGGGTCCAGAGAGCAACACTTTTGTACCCCGTATTCATCTGGTTCGACTGCATCAACTGGACATATTCGACCATAGTTCTGGTCGAATATCGACGTTTCAGGCCGAAATACGACCTCTGCCGCCGTTGCGCGGGCGACTTCCACCTGAAACAGAGGGGTTCCA-779-**Hlac_0001 (Plus strand, 780-2453)**

1. **Hlac_1078***

1076676-GACCGCGACTAAGGCTGTTGAGATCGCCGAAGTCATCGAGAAACCCGATGATCTGCGAGCAACCTCGCGACAATCGGACCCTACAGGCTTACGCTTGGGATCTTCCACGCGTCCCACCTCGTTCAGCCCTCTGATCGTCGTTCACCCCTCTGATCGCCGTTCACCCCTCTGATCGCGACGAGTGTGAACTCCCCGATGCGGCCGGCCCTCTCATCCAATCCGATCGCACCGAGGAACGACTGGACGAACTAGCGGGTGAGACCGACGAAGCCGTGGTCGACGATTGATTGGTTGATCGCTGAACCGAGACGCACCCCTCATTTCGTCTGTTGTCCGCGCTGGTAACCAACGGTCAAAGCACGTCGGGCTCACACCCCTCGTTTCGTCTGTTCTCTCAGCAGACCGATGGGGACGCCACGATGAGCTATCGGCCACACCCCTCGTTTCGTCTGTTCTGTGCCACACATTCCGAGTACCGATCTCTGATCGGCCGCAGCGACAAAACAAGCGAAACAAAGTAAACAGACGAACGCCACTTTTAAGTTAGTAAATAGTGTTACTCAGAATATCAACG-1077249-**Hlac_1078 (Plus strand, 1077250-1078446)-**1078447-TCAGTTTCCAAACGGAAGAACAGACGAAATGAGGGGTGTCGAT

GCCTCAGAGAGGACCCTCTAGCTGAATAAGAACAGACGAAATGAGGGGTGTGATTCAAGCGAGAAGGACCCACCACGGACTCCAGCGACGAGAACAGACGAAACGAGGGGTGTCCGGACTCCACGAGCCGACCGTCAAAACTCCACGAGCCGACCGTCAAAACTCCACGAGCCGACCGTCAAACGCTGTCGAGCTATCGCACTCTCGAGCCTGACCGAAACCCCGACAAACCGCG-1078724

1. **Hlac_1524**

**Hlac_1524 (Minus strand, 1541528-1542730)**-1542731-AGGATCGACACCGGGTTTCCGGTGAAATGCGT

TCCGCTTCGACCGTGACGAAACGGCGTTTGAGACACACACCGTGTTTCCGGTGAAATAGGGCGAATGGGGTGGGGAGGGAGGATCGATGGGAAAATATAAAACGGGCCGGGGAAGACTCGACAGCCAAATTCACCGGAAACCCGGTGTCACTGCTCCAAATCGACCACCAATAAAACGGATGGCGCAGAGAGAACAAATCGGGGGGTACAAACACCACAGTATCAGGAGACGCGTATACGATAGAGCACAAGACGCCAATTAGCGCATAATACACGAGAAAGTGGAGAAATACAAAGGAGTGTAGAGAACGAAGAATACAGATACGAGATACGAACCCGAAACGGGAGACCGAATTTCCGGTGAAAGACTAGCCTTCGGCTCCTAATTATCTTATTTCTTGGCTTGACGGACTATACTCAAAACCTGTTTATAATTCTTTCTATGATATAACAGAGATTGAGGCGACGAGTCCGATATCTAAAATCGGAATCTACCACCACAGCCCACCACAGCCCGCCACCACTAGACGCCCACCATCCACTAACCCCATTTCACCGGAAACATGGTGTGTCTCTCAAACAGATAACTCTCGAACAAGTCACCAACGGTCATTCACAACTGTCGCCTACAACCGTCAACAGGCAGCTGTCGACAAACAGGCAGCTGTCGACAGATAACCGCACGAGATGCAAAAAATCGATCGACGAGCCGCGAGGTACCGTGAGTCTGTTTACCCCACCTACCCCACCTACCCCACCTACCCCACCTACCCCACCTACCCTATCCTACCTACCCTAGCCTACCCTACCTGG-1543575

**Chromosome II (4)**

1. **Hlac_2747***

TTTGAATGATGATCAAATAGGGAGTCACACACCACCCTGCAAGTGTTCTCTAAATACGAAAGCGTCTATTTCACTCAGGTTGATATAAGCTCGCAGTATATAAAACGGATTCTTAAATAGTTCTTTACTAGACTTACTACACTCTAAAACCGATAGAAGGAAGCACGATTTTCAATTCAAGAATACCGGCAACAACAACAACAACAACAACATTTATAAAAACAGTAAGTAGTAACGATGTTTCTACCTGAAATTCTATCGACTCAGCCGTTCTAATGTATGTTTAGCAGGTTACTTACGTCCAGTTATTGGGAGTAACCCCTCACCTTCGGCTGGAAGAAAACACTTGCAGGGTGGTGTGTGAGGGGCTACTTCCGAGAACACTTGTAACACTTGCAGGGTGGTAGAAGCGTTTATAACGGTACAGTCTATCATAGTTAACAA-225-**Hlac_2747 (Plus strand, 226-1554)**-1555-GCGGAGCTCTATCAGTATAACACTTGCAGGGTGGTGTGTCGTCTAGAACACTTGCG

CTTGGTGTGGTGTTGAAAACACTTGCAGGGTGGTGTGGTCAAATACGCCGCGTACAATATTCTTTCACGTGGACTTGATCAAGTAGGAGTGATTCACTCCCATTCAATGCCTACGGAGACTGCGCTCTCTACGGGAACCGATTCGGTTCCTACAAAGCGCGTCTTGGAAACAGGAAGCCCCGTCCTCTGTGAGGCCGTAAGGTCGATCGGGGTGAGGTAGTTCACTTAGTGTAGGTTCAAGTTTTGTCTTATGAGTGATGGGCTTTCTATGTCTCTCGATTTGAGCTAGCAGGTCGATGCTTAGAGCTACACGTAAAACACCCCTCTATCGATGTGTAAACTTCCAGCTGAGCCTATCTATCGTCAGGACGCGATATATTCTCGTACAGACCATCTTCTCGGGCTTGTC-2019

1. **Hlac_2833**

**Hlac_2833 (Minus strand, 95530-96762)**-96763-TGAATATCACACAGTGTGAGTGGGGGGAGTATAAAT

CTAGTGGAGAACGCCACCCCACGTTTCCGACGTTCCTTGTCCGATATCAGTGGGAAAAAGCCGTAATACGGTAGTATTGCATTTCTGATAATATAACCGCGTTTCCGACGTGAGATAGGAAACCCGTAGACGGAAGCGTCGCCTCGATGTCGAATCCCGAACCCACACACCCCACGTTTCCGTCGTAATCACTGAATAGGTGGGGTGGGGACTCGAAGAAGACGTGCACGACGAGATACCAAAGACGAGAGCCGCCTAGCCGTCCAAAGTTGGCCGTCTATAGAAGCAGCTGGAAAGGATTGTTTCATCTGAGGGGGCGAAGTACTTCGTCCCCTCCTTGGTCGAAGCACACTTAATGGACATTTCTCAGCTTTGCTACTTTAATCTATGTGTTCCAGAAGTTAGTTCACCTCTAGATTGGTTCTAGGTCAACATAATTAGTAGCTGCATAGCACTATCATAGCCTCTCCCTACGCCGTCGTGGAGAAACGACGGAAACGCGGGGGGTGTGATCCAATCGAAAACAGTGTCACCCCCTTCACCCACATCCAGTTACGACGGAAACGCGGGGGGTGTGATCCAATCGAAAACAGTGTCACCCCCTTCACCCACATCCAGTTACGACGGAAACGTGGGGTGGGTGTCCTCCATCATCGAATT-97458

1. **Hlac_2958**

**Hlac_2958 (Minus strand, 209219-210445)-**210446-ACCTACACATCCTCACAGTAGTATATAAAACCAC

ATATTCATGTGTTACAACTGAACGTCATGACCCGTTATCTTCCTCATTCAGACACACCACCTTTGCAAGTGAAAGGGGTGACAGAAGTAACGCAAGTCTCTCGTCTGGTGGTGATTTTCTGTTCTGTACCATATCGTATACGACACCAGATCACGGCTAGTGAATGTGACACCCCACCGTTGCAAGTGAAGCATGAACGACACGGATAAGTAAAGCGGATCCAGTAATGTGGGTTAGTTACAATAGTTCAAACAGTGTCCCACACCACAGCTGCTGCTGTCTTCACTCGCAGGGGTGCTGTGTGACTAACACGAAAGAATCTGAACAGTGGAGAGGAGGAACATCAGATAGTGATAGGAACGACAATTCATCGGGGACCATTCATCCGGACTTCACTCGCAAGAGTGGTGTGTGTCTGGTCCACTCCTTCGAGGGGTATCGCGCTAAAGAGGCACTTGAGCAGAGCCAGATCGAACACGGGATGCGCGGGCTCACGAAACACGGCCACCACCTCTCGCTGGTCGCATCACTCCAACTGGCCCTCGAAGACGAGACGCCCGCGAGAATCCGCGACGTATTCCCGCTGTATCGACGCATCGCGGAACACTCCGACGTGGATCCGCTCGTTCGCCGACGGATGCACGATCACCTTGCCGATCTCGCGATACTCGGAATTCTCGATAGACATGCGCGGAACGAGGGACGTGCCGGCGGCCAGTACTACGAGTACGCGTTTAACGTCCCTCTCGAACTTGTTATTCGCGTGGTAATGGATTTTGAGGGCGTCGCGTTCCCACGAGAGGTACGCATGCTCAAGGAACACTCGACGTGACATCCTCGCCCGCCGTGAACGGCGGGGCTTCCACCGCAGGTGGAATACCAGCAAATGTACTGGATTGAGGTT-211383

1. **Hlac_2997***

257140-TCACATCCGTTGGCGGCAAGTGATCCGCTACCACGGAGAATGACCCCTCCCTCCCCCCGTCATCGATGTGTAAACTCTTCAGAAGAGGGGAGGGGGTGGAATGTGAGAATAGTCAAAACAGCTAGAAACGCCGGAATACGTCTTGAAACGTGTTTAGACATCCAAATAAGGTCTAAATTGGGTTAGTCCTTTGTCTGACGAAGATTTATATGACCTCCATTATAACCATAGCCGTAATGTACCATTACACGAGGTTTTGAGAAGGTTACTTCAATAAACCGAGTAATGGTATAGCCGCTGATCAGCGCTGTCGTTCTCCCCGATATCACCGTTATTAGACTCTAAACCAGCTGTCAACGGTTTCACCCGCACTTTCGCCCACCCCGACTCTCACATGTTTACACTTCGATGACGGGGGGTGGGTGTTCAATACTCGGGACAAACGCAGGTGATACTCGTGGTCTGGGTGGAGAAATGGGCACTCTCAACTCCTGCGCGAAATCCGAACTTGTCGAATGCGAGAGCCGAGTCACGCTCCTATGAGATCTGATTCGAGTTTACACATCGAATACGGGGGGTGTCTCTCGAGAGAATCTTCTGAATCTATCTGCAAATTCA-257758-**Hlac_2997 (Minus strand, 257759-259258)**-259259-CGATG

TTTATCCGGCAGTTCTACCCAATTTATCTCCTATATTCGGATGATCAGTGCCGTCTCTGTTGGGACCGACACCGAAGTATTTCCTATAAGGGACTCCCCGTCATCGATGTGTAACGCGAGCTACTTCGCCTTCTTGCTAATCTGATGGTTCGATATTCAGTACAGTCAATTCTACCCACTCCATCGACGTGTAAGCTGTCTGTCTGACACCCGGAATCGAAGTGTAACTTGTGGGTTCCACTCCTCGTCATCGATGTAAAAACTGGTTTGGTCTTCCAGTCTTCCGACTGCCAGCTATCGATTTACACTTCGAACACGGGGGGAATATTGATAGAAATCTATTGTCCCCGACTCCGTCATT-259624

**pHLAC01 (4)**

1. **Hlac_3367**

**Hlac_3367 (Minus strand, 122508-123887)**-123887-CGTGCATACTTCGTGTATTCAACTCACATAAACCC

CCGTGTCCGTAATGTCCGACTTGTCCGAATAACCCCCCACCTCGTCCGGAATTACCGTCGTTCCGGCGACACACGGATTATTCTACACAGTTTCACCTGTGGGCGAGAACCCCACCCGGGCCCCCGGGGTTGTCCGCAATTATCCGGCAAGCGGTAGGGGTGGGGAAGTCGGTTCAACTGGGTCGATTTCCAGAGAGATCATCGATTCAGCGTGACTACCGTGGTTGCTTGGTAACGTATATAATAAACCTTTTCATTAGAATAAGTAAGTAGTAGATAGAGGAGCTAATTCGGCATTTTCTATCGCTGACTACAAGTACTTAAGTACTACAGCTACTCTACCGCTTTAGTCTCCATACACCCCTACGATAGAATTGCAGACATCCCCGGGGGCGGGGGTTGGAATAGCCGCCTCCTTCTCGTTGCTGGACTGAACGCACTCTATTAGACAACACCAACGCGGAAAGAGGTGACTAGAGCATCCCAAAAGGTTGTTCTCGGTCATCGGCGGCTTGTTGCCAGTTTCGAGCAATTCCTGTCGACCACGTCGTTCGTGAGTCTTCTCCCCTACTCTACGTTGATGAATTGCGGACAACCCCGGGGGTGGGGGTGCTGTCTAGTCGATCAGCTCGACTATCATTATCGGCAGTGTCCTAGTCGTCGACGCCCGACTCCAGAAAGCTTCACTGAGCGGAGTCACGGCTCATCGGTACGAGAGACTTGTCCGTTAACCAACGCAACTCCATGTACGAATTCTTTGTTGGTTAAGACTTGGCCGATCATTCTTCCTAACTCATTTGCTGGTCGAATTACGGACGACCCTGGGGGTCTCTTTACGGAGAATCGAGGAGAAAGCCTCGCGCTTCAGCGCGGGGAGGATGTCAAGCCCGACGAGAGAGTAACCGATCGCAGACGAGATTACCGAGATACAGGCCCAACGACGTGGTTTGTCACTACGTCCCGTACAATTGCGGACATCCCCGGGGGTGGGGGACTGTCCAGATGA-124928

1. **Hlac_3512**

253972-GTACCATACTCACCCCACCAGTTCCGCTGTTAGCTTGATACATTCTCGTGGGTCATACCCCCACTAGTTCCGCTGTTAGATAGACAGTGTATACGGTGTCGAGATGGCTGTCGGTTTCCCAGATCTCGATTCAACGATCATCGTCAGGTTGCAAAGTAAACGCGAGTATATCACGGTCCTTTGCTACACGACAAGAAGAACAGTGAGGCAGACCCACCCCACTAGTTCCGCTGTTAGTTCTGAACCGTGCAAGCAGCTGTCGACTATCCACCTCACCCACCCCACCAGTTCCGCTGTTAGACTAGCAAACAACTAGCGGTCGGTCGGCGTATTGCCGTGTTAATGTATATCTATTGAAGATAGATATATGTAGTTATGGTGGAAACGAGTCCGTAGGACGTTAACACCATACGACAAAGCACCCCCTGACATGGGGGTGGTGTAGTCGTATACGATTTTAACAGCGGAAACAGTGGGGTGGGGGGGTACGACTAAGACGCGTCTGCAATAGGTATAGAACAGACACACCCCTCCCGCAGAGTGGAACTGAGATATGCTGACGTACCTCTTGTCCATCGCGACCTGCCACCGGAATCTCGAAGTCGACTTACCTTCTCGACTCCAAACAGACACACCCCTCCCGCAGGGTGGAGAGTCGATCAAGTGTGTATGGGTGAGCTGATTACGATGATGCACTATATAAACAAACCAAAAATAGAGTATATAGGAAAGCAAAAACGCAAGACGCACCAGCATTACTTGCACTCATATAGAAAGGCATAACAAACACGTTACTACTAGCTAGTCTAGCAAACACGTTACTACTAGCTAGTCTAGAACCAACTAGTTAGTTAGTTAGTACAGAAAGAGAACACAGGTACTAACAAGCTACACAGCTGGTCTACAGACGTCTTCTTCTCTAGCGACCCCCCGACCGCTTCGGCAAGTTCCACCCTGCGGGAGGGGTGTGTCTCCCCCGATCACGAGATCATTCGCCGTGCAGACCCCGGTCGACCAAACGCCGGCGTATTGAGCCAGTGAGTCCATAGACACTCTCGATGCCGACCGGCAACTAAGTTTTCCACCCTGCGTGAGGGGTCCTCTACGCCACAGTGGGACGGTCACCTCGTTCCACTCTGCGGAAGGGGTGTCTCCCCGACATATCGGATTGGCCGAATAGTTCCACCCTGCGGGAGGGGTGTCTGTTTGTAATATATCATAATACAGAATACTGTGTTCCACCCTGCGCTTGGGGTATTTGCCCAACATATCGGATAGATCAAATGGTTCCACCCTGCGGGAGGGGTGTCCCTATATGTCAGAACCTATGAAAAATTCCACCCTGCGGGAGGGGTGTCCCCAACAGACCAAGATAGCTGGTCAGTTCCACTCTGCGGGAGGGGTGTCCTCGACAGATCAGAACAATAGGTCAGTTCCACCCTGCGGGAGGGGTGTCTAAATCGACTTTCAGCGAATGAAATCACAACGCTCGAACTCCATAAACCGATAATATAG-255487-**Hlac_3512 (Plus strand, 255488-256777)**

1. **Hlac_3539**

**Hlac_3539 (Minus strand, 287018-288481)**-288482GGAACGACCGATACAGGCTAGTAGTAAAAAGGTT

CCCCACCCCTATCGAAGTGTGATCTCCCGTTTTAGAATCTCGTTCGCTTTTGTGAAGCCGGTGTCAGCAAATAAATGATGGAAATACGTAACCGCCGGCGATGTGTTTAGCCGACACACGGTCAAACACACACACCCCTCTATCGATGTGTTCGATGTCTTCGGTGGGTGGGGGGTGAGTCGAGCCACTCGGTCGGGTGGATCGTCGGGAGATGCCGAGAAACCACTTAGATCACGCAAGACATCGTCTGATAGAACCTCCTCGTTTCTCTGTCTTCCTCTTCTTCTTCTTCTTCTTCTTTTCTAACAACAAACAACACACACACACCCCTCTATCGATGTGTTCGATGTGTTCGTTAACGCCAAATTCTGTTGGTGTGATAGCGCCAAAATTCTACCTACATTATTCAGCGCTATTCGGCGCTATTCGACGCTATTCACCCTTAATTCACCGTATAACGTCGCCTTCTCGGCTGATACCCACGAGTACCCGACAACAAGTCGCCGTCCTCTGACTGTTGTCCGGTCGACGCCAATCGGTCGAACACATCGATAGAGGGGTGTGTGTCTCTAATTGGATCCACTGATCTGGAGTGAACACATCGATAGAGGGGTGTGTCATGGAGCGAGATGTGTGGTGCCGACTGTCTCGAGTCAGTTCGTGTCACTCGATCTCCTCGGATCGCCAGTGATCTCGGCTGAAGTCGTGAGGTTCCACCTTCGATCTTGGTCTTAGGTCTATATCATAGATATTCATCTTAGATCTGCATCTCAGGCTTGTGTCTTAGCTACTGGACTCGTTGCAGGCGGCGATACCGATGATGTTGAGTTCTGTCACCGCTTCAACATCGAGTGTCTCCGGGCGGCGTGACGGCTGCAGTCTAAGCGGGCCGATGAACGACTCTGACTGTCGCGATGAACTACCCTCACTGCTGGGGAGCTT-289457

1. **Hlac_3641**

401836-ACCTCAATTAGAGAGGACACCCACCCCACGTTTCCGTCGTAACTGGATGTGGGTGGAGGGGTGGCGCTGATTTCCAAGGGACCACACCCCCCGCGTTTCCGTCGTTTCTCCACGACGGCATAGGGAAAGGCTACGATAATCCTATGTAGCTACTAATTATGTTGACCTAGAACCAATCTTGAGATGAACTAACTTCTGGAACACATAGATTAAAGTAGTATAACTAAGAAACGACCATTAAGTGTACTTCAACCAAAGTAGAGGCGAAGTACTTCGCCCCCTCAGATGAAACAATCCTTTCCAGCTGCTTCTATAGACGGCCAGCTTTGGACGTCTGGGCCGCTCTCGTCTCTGGTAACTCGTCGTGGACGTCTTCTTCGAGTCCCCTCCCCACCTGTTCAGTGATTACGACGGAAACGTGGGGTGTGTGGGTTTGGGATTCAACAATGAGGCGACGCTTCCGTCTACGGGTTTCCTACCTCACGTCGGAAACGTGGCTATATTGTCAGCAATCCAATTCCACCAAATTACGGCATTTAACCGCTGATATCGGACGAGGAACGTCGGAAACGTGGGGTGGCGTTCTCCACTAGATTTATATTCCCCCGCACACACCGTGTGATATTCA-402464-**Hlac_3641 (Plus strand, 402465-403697)**

**12. *Haloterrigena turkmenica* DSM 5511** (CP001860-66)

**Chromosome (7)**

1. **Htur_0001**

CTGTGGGGCCCTCCAGCACCGCGGGGTGGTGCATCTGCGCGTACTACAAACCGAACCCACTTAAGCCTACGTCAGACGTGTAGACTATTCAACGAAACGGAGTTATACGGACAGATGGGGAGATCATACTAGCGGAACCGATAAACGTACTAGTAGGATCGATAAATGGCTACCTCGACCGATAAATAGTTGCCTCGACTGATCGATGGCTGCCTCGAGTCGAAGCAGGGGGTCGATCGGAGCCGGGACTGTGACTACGGATCAGTCGGATGGCGACTCGAGAACCGCGACGGAGCATTGCATACAGTGATAGACTGACACAGCGAATACGCGTCAGTTATCGAGTGTGTGTTGATCGGTACCAGATATCACGATATGAGGGACGCTATAGATCGGCATACTAGTGTGAGTTCAAACATCATACTAGGAACGTACGTAGATCCGATGGAAGCCCCCCACCCCTTCGTTTCGGGTGGAGAGTCGCGATCGTGGGGGTGGGGGAGTGGTATTGTTCGGGTGGAAAGACTCTGTAGCGACCCCGAGATCCCGTTCAAACGGACAGTACAGACCGTCAACCGGTGAGTTTCGGGTCGAACGACGTCTCTATAACTCGAGCCGTCTAGAGAGGCTGTTCTAGTAGGAACTAGCAACTAGAACCAACATCTCTAGTAAAAATCTAGTTTTGCTAGTGCTACGGACCGAGTTTCTGGCCCATGGAAATAAAGGTTCCCGTTCTAGAGACCCCCCCTCCCCCCACCCCCTCCTTCGTAGCGTTCCACCTGAAACGAAGGGGTGGGGGGCTAGGTTGATCTCACCCTTCGTATCCTGTTCCATGTTACCTCGAATCTCACTCCTTCGATGTCTGACAACTCGTGGCACACTCGCTCTCGAGAACTACTCCGAGTGCTGACCGAAATTTCTCGCCCCTCACTTACTATCATACCTCTTATATTTATCGCTGGTGTCCTCGAGCAACAGCCCATACCCGACTCGCTTCGAGGTCGAGTGACCGAACAATAATTGCGGTTGGAGACGACTACTGAGTGTTCCTGACCTAGTCGATCGCGTAGGTGTCGATCTCGCACACGCATCGCGAGCGCGCGTAATGATCCCTTATCGACCCGAAACGGACGTCTCTCACCCCGTCGTCATCGGTCTCGTGCCTCGATGGTCACTGGACCTTTCGAATAGGCCGTCAGACGGCCGATACCGCCGATCGTGCCAAACCCTTGTGGAGGGAAGGGAAGAATTTAATACCGTGGTTTTCCTCTGTTTCGTTTGCATCAACTGGACTCGGACCAGGGAGGCGAACGAGTGGGAGACCCTATTCGCCCCATCTACTGCGTGATATTCCTGCTTTCGGGTCCGAATTCCACTCGAAACGAGGGGGTATGTGTACGACGG-154-**Htur_0001 (Plus strand, 155-2011)**

1. **Htur_0510**

542216-CCACGTTTCAGTTAAACTATATAACCTATGTGTTTCTCCGCGTATTCCCACCCACGAGTGTCCGATCACTGACTATACTCCCACACCCCGGGTGTCGAGTGTAGACGCTCGAAAGCGGAGAGAAGAGCTCGTTTCTGGGCGTGATGGGAATGGGTGATGGTTCTAGGTCTGGTTTGATAGCAAATATCGTATGATAGGATGGAAGGTTTTATTACCTACTACTGGAAACCGGAACCGTACTGGACTATTCGAAGTTCGAACGATGGTTCGAACGAACGGAGCCTTCGATCTCCGTCTCCCTGTCAATTCTCCGTCCGCTCACCGGTAGTTCTCACACGTCTCTCACTGGTTTCAGCTATCTATAGTCCCTACTTCCATTTCCAACAGCGCTCCTTAGGTCATCTGTCGGAGGTTTTCGGCGATCTACACTCGACACCTGGGGTGTGGGGCTACTCGAGTACGGCGGTCCGTTACACCGCATTCGCACTCGAATTTCGATCGCGATTTCGGTCGGCACTCCTCCGATTTGATGGCTAGCACAGCGGGCGTGTGCCGTCGACCGCGCGCGTTCCGGATTCACTCGACGCGAGGATAGACGCAGTATGACGAACGCGCTCGATAGACCCCTTCTTGCTGCTTATTTCCGGCACCTACATCCGACAGTCGGTGACTGCCAATCACTAGTCTATATGTGACCGTCCTCGATACGTGGTGTGT-541499-**Htur_0510 (Plus strand, 541500-542753)**

1. **Htur_1155**

1210406-ACGCTGTGTCCTCGAGCTACGCTCATAGAAATACGTGTGTCAGTGTCATGTGATCCGGTATTGTGTGCGTTTGGCTACACTATCGCGTATCGAGATGACGGTCGTCGCTGTCGAAACGCGCGCGATTCGACGCAACTCGACGACCGGCCGAAGCGCCGAATGATAGCGACGGACACCCCTGTTTCAGAGTGAAAGGAGCGATGGGGACGGGAGGGATGGCGGAACGGCTCGAGAACGGACGGACCCGTCGTTCAGAGTGAAACGACTCTGAGAGTGTCAGCCCAGTTCTGACAGGGAAGAAGGCTTTCACTCTGAACCGGGGGAGGGACTGCCGGTTCGCATCCAGAAGGGGCGGTGGATTCAAGCTAAGAAGGCCTCAAAGGCGAGATATACTGGAATAGCGCTGGTGGAGAGGACGTGGTGCCGAGACGAAGCCAGACTGGAATTGGGGTCGAGGGTCCCTCCCCTCGTTCAGAGTGAAACGGTGTTCGAGGCCGGGGAGGCCCTGTACTCGGTGGTAGAGAGAGAGCGTGCTCGAGTTGTTGCGGGGGCTCCTGACATGAAACCCCACTTTTGGTATTTCAATACTAGGATTGGTGACTATACTACTCCTACCAAAAGATATAATACTACTATATCTAGCCGTATTCTAGACTAGTAGAAAGAAAGCAGAAAGAAGGATGAAGAGAAACCGGAAAACGGGACGAACGGAGTTGACTCAACATCGTAGTGCTCTCGCACTTGTCGGGAATCGTTCGCTGCCCGAGTCGTCTACGCTGTTGCCTCAGTCGTCCACGCTGTTGGCTGCGTTATTCACGACGGGTTCGGCGAGCAGTATTCAGTGAGTGCGAGGAGTGCTGATCGACGAGGGGGGTGTCCGTCGTGCCCGTTTCACTCTGAACGAGGGGTCTGGGTCTCCCGATCGGCACTGCAGTCCGTCGACCTTCAGTCTGATTCGGGAAGGAGATCAATTCGAGCGGCTCCGGTCGATACGATGTCTTTGAGAACGTCTCGAGTCGGTTTATCAGTCTGAACTACCTTCCACAGATTTATTAGGTCGCTCTTCGAGCACGGAGAC-1211483-**Htur_1155 (Plus strand, 1211484-1212767)**

1. **Htur_1843**

1917656-CGTTCGATGGAGGATATCGACTCGAGGAGCCGTGTATCCGTTTCGGTTCCGTCCGTTCGCAGACGTCGGCATGGAAGTCTATCGCTCGAGGCGACAGACCGAGAGGGCGGCGACTCGAGAGACGTGTCGGTCATACCTCGACTATCTGCTGCGGTGGCAAAAGGGACGCGTTCGGGAGTCTGGGAGTTCCGTTCGGAACGGTGTTAGAACCGACTCGGAAGGAGCACACTGACGGCTACCTTGACCCTATTTTGTCTATCACTCTTTATCGACCTACTTTGTCCACTCTACTTTGTCTACTACTCTACTTTATCTACCCTACTTTAGTTCTAGACTAGCTAGAACTAGATCTAGATGCGGTCGAAGCCGATTCATCGATCTGTTTGATCCGTGTTTCTGGGAGTCAGTTGGTTCTCTGGAGTCGGTTGGATCCCTGTTTCCCGACGTTGTGGTTTCTCTGTGCAGTTCTGGACTGATACGTCCTCGAGCGATCGGGTATCGGCCGAGTTGTTGAGGGGACACACACCCCCATCGCGTTTGTAACGCCGTCTAGGTGTGGGGGTCTCTTCGTGGAACACACCAGTGTCGCGGATGTAAATTTGGGGAATCAGTGGCCCGAATCTCGTCGAAATCCGGAATAATTCTGCATCCCCCTCCCCACCTTCCCCGTTACAAACGCGACGGGGGTGTGTGTCCCCTCCGTGTCAGACGGACGTCTCACATGGGGCAAAATGACCCGCTATCCGGGCTCTCGGACGGGTTCTTCTCACTTCCGTGATGTCTCCGCTCGGTTTTCCCGACTTCGACGGCGAATCGAAGTTCGGCGGCTGACCAATGTCAGCGTTACATCCGCGACGGGGGTGTCGGTGTTTGACACGCTCGAGTCACCGTCGTGGTTAGAAGCGCGAGGCCGCCGTTTCCTGTCAGAACGATCTATGTGGGCACAGACGGCTGCCTTACGGCTGTTCCGCCGCCATTTACATCCGCGAGCGATCTCCATACCTTTATTTCAATCCAGCGGGAAGGCACGGGGTACCGCA-1918695-**Htur_1843 (Plus strand, 1918696-1919937)**

1. **Htur_2434**

**Htur_2434 (Minus strand, 2503283-2504566)**-2504567-TAGCGAAGACTTTCCCGACGCGAGTGATAAAC

GCACCGAACCGGATTTTCAGTGAAAGACTCGAGACGGGCCACGAGAGTGAGTGACCCACTGATCGGGTCGTGGACGGTGACGGCACACACACCCCTCGTTTTCAGTGAACGGCCGCGTAAGTGGGGGTCCGTAGTCCGCCCGCAGCCCGAGCCCCTTGATCGCGTTATTATATTATTTATTGTATCGGTAAAGTAGTAGTGTAGTTGTGGACCAACAGACTCTGAAAATGGACTTCAGTACGCAACACTTTCGATCAGCGTTTTCTTTATACTATGGCTCAATCTCCGCACCTACGGGTTTTCGTTTTCACTGAAAACGAGGGGTGTGCCCGTCGTCGTTCTCCGTCCCGGTCACTCGAGTCCGACCGACGACTGATATTGCACCGAGCTCTTCACCGGAATCGCTGTACGCGAGTGTCAACAGTGGTACGCCTCAGGTCGCTCGCAAGGTTCTCGGTGCCGGTGCTTTCCTCCCAACAGCAGAACGGACTCTGCGTATCGCCGCGCTCGAGCAGACTCGGACGTGAAATGACTGGGATACAATCGTCTCCCGTCAGCAACTTATGAAAACCACCGATATGTTTATCAGCGTTTGCTCGAAAGCGTGACGTAACTC-2505215

1. **Htur_2559**

2627297-CGTCGACGCGAACGGAACGGCTCCGTCCCGACCTGTTCCGTCCCGGCCCGTTCCGTCCGAGTCACGCGTCGCTGTTCTCGGATAGCTTCCTCGAGTCATGGACACGTCTCAGAGAAATCTGTTTGTCTGTTTCGGTCGTCTCTCACACTGTATTCGAAATGAAATCTCTCTATTGAGTAGCTAGAGAAGAGAGTCTAGAAGAGGTACCCTCACACACCGTTGTCGAAATGAAATTCGGTGGTCGAATCCGGATACTGGCTCCATATTCGGTATATGTGTCGAAATCACTAGTTCCAAACACGGTTTCCGAGATTTCATTTCGATCACGGTCCTCGGTTCTCGGCCAGCGAGCACTCCGTTTCGCGGTTTCATTTCGATTACGGTGTGGGAACGGTCCGGTCCGGCCGCTCCTCCAACTCGATGGATCGACTTCATTTCGAAAACAGTCCCTTCATTTCGACAACGGTCTTGTTTCCGCTTCATTTCGGTCACGGTGTCTTCATTTCGGCGACGGTCGGCATCCGATCTTCATTTCGAAAACGGTGCCTTCATTTCGCTGGTGGCCGTCTCACTCCGATACCGGCGATATCTCGGCTTCATTTCGACTGCGGACTCTTCACTTCGACCACACCGTCTTCATTTCGACTGTAGCCCCTTCATTTCGAAAACGGTATTACAGCGTAGTGAACAGTGCAGATA-2627919-**Htur_2559 (Plus strand, 2627920-2629272)**

1. **Htur_3152**

**Htur_3152 (Minus strand, 3241079-3242266)**-3242267-CGAATCCGACATTCAGGTACTAGGGACAGTAC

GCCGTAGCACCTTAAACGCTCGGAACCGAAGTGCCGCTGTATAGACGAGATTTCCTCGAGAGAGATCCCGAACGGTGATAATCACACCCCGGAGTGCCGCTGTATAGTCGGTGACGCATCGCTCGGTTCCGCTGTATCGATTTCGGAACGACCCGTGGCGACCGGCGGGAGAGCGACTGCGGAGGCGACCCCGGAGTGCCGCTGTATAACTGCGGCCACACCCCGCGGTTCCGCTGTATAGCGACGCCGAATTACCGCGATATCCGTCGGGGGACCCCGGAGTGCCGCTGTATGCCGGTGGGTCACACGCGACGGCCGGCGCCCGCTCGACCGCAACAGAGAGGGAGCCACGCGGCCGTCACCGGGGGTATCGGAACCGAACCGATGAACGGGGCGGATACCGTAGTTCCGCTGTATCGGATAAAAAGAGTTTCTATCGGAACTGCACCGTTCGGTTTCCACACCGCGGTGCCGCTGTATGCGGAGACACACACCCCGCGGTGCCGCTGTAACGGTGTGACGGTGGGTGTCGACTCGAAGCGAGGGTGAGGCGGGGTGGATGGGGTAGAGTCGGGCAGGGTCGGGTGGGGGCACTCATGGAGCCACGGCGGTGTGGAAACCAGTCAATAGCGCTCGGCGAACGTACCGAGACGGACGGTGACAGCAACCGCGGTCAGCTCGATAGCGTCAGCTCCAGGAGAATCATCGACGGAGCTGCTGGTTGAAGCGCCGTGACGAACTCGGCGATTGAAGCAGTAACCGAAACACCGAGAGAATCGGCGATTCCGACCGTCGATCCAGAACGAAGGGTGGAGTTCGCCTCGAGCGGCGAGTCCTCGTCCAATACCGATTGGACGGCAAATCGTCCAATACACTCAATGGACGCTAACACAGAACTAGTATTTAGTAGTGGTGATTAGTCATGGATTCAAACAGGCGACTGAAAGCGGTCCAGCAGTCGTTCACAGCCGATTCCGGCGTTTTCTCGAGCGACGCGGGATCTCTCGGACCTATACAGCGGCAACGAGGGGTGTGTCTCTCGAGTCTACCCACTACTCTCGCGTAGATAATGTCGAAATAGCGAACACATCCTCGAGACGCTTTCGAAGATCGAGCACTGAACGACAGTCGAACGTCCGCGAACGTCGACCGAACAACGACGAACGGCAATCGAACAGTCAGACGGCAAGCAGAATCGAACAGCCGAACAACGAGCAGCCAACCGAGCGATGATTGTCCGAGAGAACTCGAGCAGTCGAGTCGCGATTACTCTTCGGTGCAAACCGCGACAGGA-3243592

**pHTUR01 (1)**

1. **Htur_3916**

**Htur_3916 (Minus strand, 171356-172588)**-172589-TTGCTAAGAGATCATGTGAGCGGTGGGAGTATAA

AACTAGTGGGAAGACCCACCCCATGTTTCCGTCGTAATTCCGGAGTATGTGTAGAATATAGGGATTGAAACGCCGGAATAAGACAATTGATAGTATAACCGGATTTCCGACGTAGACCGGAATACCGCCGTTAACGGAACGTGAGAGACAAAAAAGAGTGAGAAGTATTCACCCCCACACCCCACGTTTCCGTCGTAATTCAGTGAGAGAGGGTGGGTGGAATTGAAGAGTTCGTTAGAGGAAGAGAGATCTCCATAGAGAGGTCACGTACCCAACTAGAACAGGTAGGGGAGAGACTATAGATAGACCTCTGTTTCAGCCGACGAAACGAAGTGAAATTGTTTCGTCAACAAACGAAGTTAGCTTAGTGGACTCTACTCAATCTAACTCCTTTAATCTAGGTATTGGAACCAGCCATTTTCATCTTAGAAGCGGTAAAATAGGCCGTTATTTGGTACCCCTCCCCACCTTTCCTAGAGAAACGACGGAAACGTGGGGGGTGTGGGTTCAGCGAGTTCATCTACATCCTTCTGTTCATTCCTTGTCTCGATACGTCGGAAACGTGGGGTGAGTGTGTTCTCTCTTCTTTCGGTCTCCTATCCTATCTTATCTTCCCGCATCACGTAGCATCCTTCCGAAAGACCAATCGGTAACTGTGTGCACCTCCCGAGCAGCGTTCGATTGATCGTTCCAGCAATGGTTCTCTCACATATCTCCGTCTCGTCGTAATCGAATCGATCGAGGACTGTGGGAACGTCGACTGACGATAAAAAGAGTATCTTAAGGCGAAAGCCCGCTTCGGGCAGGGAGAATA-173436

**pHTUR02 (1)**

1. **Htur_4773**

**Htur_4773 (Minus strand, 386004-3873500)**-3873501-AGTATCGGAGTGACGCCCAGCCCATAAATAAAT

GTGGGATACCGTGTGCGATGTCAAATATAGAGGAATCCCGCGGTAAAGGGGTTCTCTCCGTGGAGAGTTAGATAGTATCGAGAACCGTGGGCGAAATCAAACCGGGGCTAAGTGTGGATCGAGACACCCCGTGTGCGAAATGAAATCGGTCGCAACTGATCGGACTGAATTCGAGAGTTGATCACACGGGGAGAACAACAGACGAGAGGGGAAAACACAGTTATGGGGAAGGGGAGTGAACCCCCTCCCCCCGTGTGCGAAATGAAATCGGCTTGAGGGTGGGGGGAGGGGGGATGGGGTGTCAGCGCTGGAAACGGCGGATATCCGGGAATACCAGGGTAAAAAGGACTCTACACCAGATATGAGAATATCTCTATATACTAGGGAAGATTTATTAGCATAGTCTAGAAACAGTAACCGCACTGCAACTAGGACTGGTTATTAATCACATACCATTAGTTAATAATACGTAAGGACATTAGTAGCTATTTCCCAGACGAGTTCTTAATCTAGAGAGTTCCCGCCAGTTTATAGCCGCTATGGACGTCACTCAGCCGCTCTTGCTGGTTTCACCTGTTCCAACTTCAACCGATGGCCTTCCCTCCTTCTCACCGATTTCATCTCGCACACGGGGTGGGGGTGTCTCCTTCATTACCCACCCTTCTCCAGCGACTCCTTCTCTCCGAGAGTTCCAGTTCGCTCGAGAGGATCGACTAGTTCGAAATGGCCCGTTCACGCACGAGCTCGAAAAACTCACCGTTGAAACGATACGGGAATGGGTCGTCGTCAATGTATGGTGAGTCCATGATATCCTTACCCAACCCTCTCTTTGCAATATTCGTTGCTCCGTTTGTATGACTCGAGACCGCCTGTTTTAGGGATCGTAGCTCTCGCTCTTCCCCCACAATTTGCAGTAAAACGACCGGTGCAGGTCTTTCAAGTCATAGCACCACTTTAATTCACGGAAGACGGGGTGATGATTTCTGAGAATAACCCTACTACGGACCAGCTCAAACGTAATCGGTATGTATTCTTATTTAGAGTTTTCTCGCTACGTGGTTCTCTCGTGCACTTTACTGGAAATTGTGGGGGGGAGGGCGTTCCACCATTTCCGTCCGTCTACCTCTCCGTCTTCTTCCGATTATCTTCCCTTCCGAGTTTACTGGTCACGAACATCTCCAATTTTCGGACGACGACGGCAGGCATCGGGGCTTGTAGACAGCTCGGCTGCGACCCAACCGATCGTTCGAGACCACCTTGTCGATGGTCTAATGAGTGTGTCAGACCAAACCACGGGAGGTAGCCTCGCGACCAAGCCTCGAGGCGGTTCATTACTGCCATCTTGAACGGACTCTTCCGACAATAGCTACCTCGACTACAATCGGCTCTGTTCCTATTTTACCTATTGTAGCTGTTGTAACTGCTGCAGATGTTTGGACTACTGTACCTGCCCAAGCTATTGTGCCTATTGCACTTACCTTATCTCCAGTAGGTAGAGTTAAGGTTCTGTAGTAGAATCTAACTTTCACCAGCTGAAATAGGACAATTCGGAACAATAGGTCCCTATTCAGATTCTCTCAGCGGGTGAACGTGACA-3875129

**pHTUR04 (3)**

1. **Htur_5210**

**Htur_5210 (Minus strand, 102212-103438)**-103439-GTGTAATGAATTCAACTAAGGTAATATAAAACCA

CTCTTTCATCTGGTTCAAGTGAAGAACAGAGACACACCACTCTTGCAAGTGAAGCCATTGACGCAGTATCCACCGAAGACAACACACCACTATTACATCCAATCAGCGCACATATTATATAATATACATGTATAGATTAGAGTAACATAATCATACGAGCGACCCGGTTATTCTCCGATTGGTCCATCTGAATTTTTACTTGTAATAGTAATGTGGCTGAATAGTTCGAAGCCATAGCAGTTGGAACCAGTTACCGACGTAGCCGCCGAAATCTACACCATCGACGATCAATTCGACTGATTATGCAGATAGGAAATTTCCTAATCTGAGTTCTGACCGTCATTATCGGTGGAAATCCTCGTAATTACGCTGGAAAGCCCCTTCCCCCTCCCACTGAGTGTTACGACGACGGGTATCACATCCCATTCAGAACGGCTTCACTTGCAAAAGTGGTGTGTGAGTGTTGGTCTGAATCCCCATCCGCTCGAATCGACTATCGATATCGCGGAGACCGACTCTTTAGATGCTACATCGAAGAGACACCCCACCGTTACATGTGAAGCCGCCGGATTTGAGTCGGTACTGTGGACGATTTCTACAACGTAGCTTTGTGAGAGACTGCGAGCAGAAAACGACTGTGGGAGCGGTTCAGATAGCGGAACCGTCTCTCTGGATGCTGCTACTACTTCGTGAGAAGACTCCCCTGACGGAACTGCGACACGGATATCCCCTCCCTCCCTCCACTGTTTCCGGAGCTTTTGGCCCTCCAGAACGGCGCGAAAAGATACCAGCCACACACCACTATTTCCGAAGCTTTTGTTCTCAGAAGGATACAGAAAGACGCCAATCCACACACACACTGTTTCCGGAACTTTCGATTCGCGAAGACAGTTTGCCAGTGTAGAGTGGACAGACACGACATCGGTTTGCTCTCCTCGAGTCTGAAATCCTTCGGGAACAGACACCCCACGGATTCAAGTGAAGATCTGCAGTTGAGAGGCTACATACCCACTACACGTAATATTAGAAAAGATGTGAAAACGTTTGGATGGGTGGTGTCTCTCTCATCAGTGAGCAATTAGAGGGGAACCCTAGTGTTGCCAAGCGAGATCGTTAGAAGAGACTTTCGCTAGACAGTGGTTGGTCCGTGCTTCGTAAGAGGACAAAAATCGATAGGGGGAAGTCTCACAGCGAATAGTACGAGAGAAACTTAACGATCTCTCCCACCAGAAGATCCTGTCTCAGTCCCGTCGCGGTCGTGGACAGGGCCACGGG-104747

1. **Htur_5222**

**Htur_5222 (Minus strand, 117409-118614)**-118614-GATCGTAGACACCACGTTTCCGGTGAAACGTCTTT

ATTGTTTGGACTCGAGGTGGCTCCGTCGCTCATAACGGTGAATTACGGGGAGAAGAGAGGGTTTTGAGCGAATATATAATAATTGGGGTAGCGGAATTGGCGGGAGAAGACACACACACCACGTTTCCGGTGAAAGGGGAAAATCGGGGGATGGGGGTTCCAGTTGAAACGGGGGTTATTTCATCGGAAACCCGGAGTGGTGTCGAACTCATGGCGGACAGGAGTCGACTAGAGTAGTCACTATATCGGGGTAGTAGCGGAAGTACAGCCGCTATGTAGGGAGTATCGGGTAGTCGCTACTCTCGTTACGCTTGCATTTGTGGAACTCTCCGATCGTGACGTTCTCTCGAGTCAGATCACTGGTTCTCGATGTGACTCGAGAAAGGACCAAGAAGGGTAGACACGAGTAGGCAGTCGAAACCATCCATAGGAAGGGTTCTATCGACGCTTCAGAGAAATCTTCCGTGGCAGAGGGAGAGAGCGACAGAAGGATTTCCGGTGAAAGAGTTGTTGCCGACTCACGTCGGCTTCCTTTTTTGGTTCTACTCAATGGACTTTTTGCCGTGTAGTATTTTATATCTAATGATAAAGCGAGGAGAAACTCTAGGATTAGAGAACCACTCAATGGGAATTAAGAATATTGTCTGGAAACGGTTCTGTGTGGGTTTTCTTCTATGGAGATGGATTAATACTGTCGAGATGGCAATCTTCTCTCTCGTTATCGGTGTTTTCTCTTTCCAGAAATCGCTGCAGTTCTGAGTCCGGTCCCCCTCCTTCGGACCCCGTTTCACCGGAAACGTGGTGTGTGTGTCTTCGCTCTCCCTCCCACCCCACCCCCCCTCTCACCCTGTTTCACCGGAAACGTGGTGTGCGCGCGCTAGCAACAGGACCTGTCGTCACGGATCGCCGCTGTTCACGTTTCCGATCCGTAATCCGAAGCAATACGCATCCTTGAACGACCCAATCGATCCGAGCCAGTTCGAGCCGGGTCGGTTCGAGCCGCGATGTCACTACTTAATTGGATATCAATCTCCGCTACTCACGCATTCACCGGAAACGTGGTGTGTTCGTGGAGTGGATGGCGCGACCGACCGTCCGTTTGCCTCTCCACGATCGTCTCCCGTCGCATCGTCCGTATTCACCGGAAACATGGTGTGTCTCTGCGAGGCTGACCACGGCGCGACACCGCACCGAGAGTCAACGGTTCAGCGACGTGTAATTTCACCGGAAACGTGGTGTGTGGGCTATCGAGCGGCGACTACTCGCGTCGGGACGTCGGTCGACGCCGCCGCCGTCG-119946

1. **Htur_5244** (141243-142475)

140294-CGCCGTCCACGCGACCACGCCCGCCCTCGAGCGCGGGTGATCTAGTCCCCGACCGGGACTCGAGACGGTTCCACCGACACGCGATGTAACCGCTCGCGGGTGTTCGCTTGGGTTCGGTAGTTCTGGGTCCGTCACTCGAGCGGTCGTCGGCGGTCGATCCCGCAGACGGGCCAGCGAGTGCTGGCCGGAGACGACGGCTGTCCACGGGATGGTGAACGATCGGAATATTCGGTCGAGTCGTATTTAGTGGCGCTGGACTAGAAAGCCATACACCACGTACCGAGTGAAAATGTATGAATCGCCACCTCGGTAGCACTGGTTCACACCTCGTACCGAGTGAACAGGTGAGGACTTCGGACTGGAAGTTCATACTCATACCCACACCTCGTACCGAGTGTAGAGTGGGATAAGGAGTGCCAGTATCGAGTACTCACACACCTCGTACCGAGTGTACTTTGCACGAAATAGACAGGAGGATCCTTCTCGAACAGAACCAGAGAGAAGGGAAAGATGACCCAGTTCCATTACAGCTATCTCTTCACCGTTGCTAACTGGAAGCTGTTAATAATCTTATAGATAAATTTTTCTATATTACTTTCAGACATCGCAAGATGGAATAGCGGCCTAGACTAACCAACGGTTTCTATAACGAGTATTTATAAGATAATTCTCGTTGGTTAATCTCGGGCCGGTACTTCTACGACAATACACTCGGTACGAGGTGTGGGTTGCTGATTGACGAGTCCTCGCTACCTATCTCTCGTTGACTTCTGAAGGGCGAGAGAGGTCTATCGTTGCTTACACTCGGTATGAGGTGTGTCTGTTCTCTACCTTCTAAATATAATGGATAGCTTATCGCCCGTAGAAACTCCTGTAGGTCATATTTTGAGGGGGTCACTCGGCACGAGGTAAGGGAAGGTTTTTGAACATACCGAGTACACTGTAACT-141242-**Htur_5244 (Plus strand, 141243-142475)**

**13. *Natrialba magadii* ATCC 43099** (CP001932-35)

**Chromosome (5)**

1. **Nmag_0001**

GGAACTCGAGAGTACACACAGAGTATCGAACGGGAGGCTAAAAGTGACCTGTTCGCTGTGTGTGGTTCGGTGTCTGTGCTGGTGGATGTGTTAGGTGTGTTGGGTGTGTTGAGTGTGTTGAGTGTGTTGAGTGTGTTAGGTGTGTGGTGTGTATGTGATGAGAATGTGTGATGAAAGTGTGTGTGAGGTGAGTGCTACTGGTGGACAGCCGGAATTGGTTGGATAGTTCGGGATTAGTTGAGTTCATCGAGAGCCGTTGGGACAGGTTTGATTGGAAGTTCCTGAATTAGTGGAGTTGTTCTGGAACCGTCTGAGTTATCGTTGGTTCTGTCTGAGAGAAGTCTCTAGGACTACTCATGACTACTTCGTCGTACTACATCTAGTATTACTAGTTACTCACTTCTCTCTAGTTCTAGAACACACATGTTGAATACAACGAACAGCATGTGTGTTGGAGTAGTACACCACTCGAAACGGACGTTTTGCCGTAGAGAGAACGTGTCGACAGCCTGGTGAGGGGGGACACACCACCCTCGCGTTTGTAACGCTGTATGGCTAGGGGGGTTCTGTTTTGCAACACACCTATGTCGCGAGTGAAAAATCCGCCAGGTTACCGGTCATATTCTCCGAAATCTGGGGATCAGTCAGACCCTCCCCCACCCTTTCGGCGTTACATCCGCGACGGGGGTGTGTGTCCCGAACGGCTCGTCGTTCGTCCGGCGCGTCAGACATCGGTGCTAGTTTTGTCGCTCAGTGGGCGTGGATTCTACACCGATCTTCGTTCACACGCTGCTTTCGGAACCGGATTCAAAACGGGCATCTTTGGCCTTCGATTGGACTGCAGTCTTGCGAATTTACATCCGCGACGGGGGTGTCGGTGTCGGCGCCACAGTGACCGCTACTTGTTGCTTTCCACACGGCAGTTAGAAGCGCGAGGGCGGCTATTTCATCGCTCGCGCCTGAATCGGCGTGTTATGGCGGATACAGTCTCGAACACATGCTATTTACATCCGCGATCGATCTCCATACCTTTATTTCAGTCCAGTTGGAAGACACCCGGTACTGCA-676-**Nmag_0001 (Plus strand, 677-2026)**

1. **Nmag_0368**

356009-ATCTCGGCCGTGACCGTTCGGTATTCGCCAAGAGCAAAACTAAATCGCTCATTTGGAATGTGGTCAGTCCATTTTGTTTGCAAAACCCCATGACAGCGAGCAGTTCTCATTTGGAACTCGGTCCACACTCCGATAGAGACACATTCGGCTGCCCTACGGCGATTCTCATTTGGAATGTGCGCAGGTAGCAGTCCATCTTCTATATACAACATCTTACAGTACCACTCTTTCACAATAGTGCGAGACAGATACCCCAGGTTTTGACCCCGTAGCAGTTGGCACACCCTCCCCCCTTATTTCGAACGTACTATTGGTCCAAGCAGGGGGAGGGGGTTAGATTCCAATCAATGGTCCAACACCTTTTCTTCGCCCCGAACGTACAACTATGACGATGAAAGAGTGAGAGTTACTGTAATATGAGGACAAAAACAGCGCCCCCACACCCCTAATTTCGATCGTTTTAACCTGAACGAGGTGGGTGGGTCGAACAATCAATCGATACGACAACGCTAATTCTTGTTGGGGTATTGTATCGAGCAGTCAGTTTTGGTTGCGCCAAATAGCCGTATTCTCATCACTCCCTGAATTTCAGCCACTCATATGATCTCTACGAGATTAGATCGGCATTCACTACTAAGTTCTACATTGCAATTGCACAACGATCTGTGTTCTTCACAGTAGCGTTACTCGCTTCACGCTATGACGGATCAAGAGGTAGTGATAGTGGGTTTCTGATAGTACAAGCGTCTATAACTTCTTTAGGAAATAAGATCCTATACCATAAGAGTTAAGTTACTATTATATATTAACATTTATTAATTTATAACAAGGTAATTAGCCACAATCATCTACACTAAAAGCATGTCATAGAAGGGTTTCCGTGAATTCAGCCAGTGGTCAGTACAGACGCTACTCATGTGCTGTCGTTGCTAATGAAAACGCTCGTATCACATTCTATTGCTGTTTCGAATGCTCCCACAGCTTTCTTCATCGAATCAACGATATCGTTACCCGACACTTCTCGTACCTCCGCCCCCTTATTTTTCCACATCAATACTATCGTATGTTTTGATTTCTCTTAACTGCGTATTCGTTCCTCGACGGATTAAGAAAGGCTGATCATAGACTTCTCGGTTCTCACCGTACACTGTAACTGATCGACTGCAGACGATTCTGTAACACACCGACTCTAAAGATAGAAATTCTACATGGTACACAAGCACCTCACAGTATAGGACCGCTTACCCCCATCAATAGCTCTAGCCCACGAACCGAGTTTAGATGCCATCCACGTTGTTTAAATGTGGACTATTCGATTTCTTTGACCCTACCCACCCCCTCAAACCCGATTACGCTCGAAATGAGGGGTGGGGGTGTTGAGAGATAGTCCAACAAGTTCAGGTAATCCGCCTCAAGATAGCGGTAAACAAACCCAGATCCTTTGACCCCCACCCCCTCGAACTAAATTACGTTCGAAATGAGGGGTGGGGGCGTTGAGAAATCGTTCCGGATCTCTCACTCTATTTTTAAGGAAAGAATCTGACTATTCATGGCAGCGTAAATCCGACAACACCTGCGCAGCCTACATTCCACAATAGCACAGTCTTTAATCTATATAAGGATTCTAATTTTATATTACACTAAGGGGGCACGTATGTCCACTTATTCGGATAATATTGGAACTCCAAAACCGGGCAGGTTCGAGACACTCTCTCGAACCCCTGTGTATCTCGTTACAAAATAACTCCAAGTCCTCTGCTGGGATACGAGTGACGGCGGTGTGGCCCCACCAGTAGCCTTGTCGTGCCGACCGAGAGACAAATCAGCATATCCCAACCCAGTGGTACAGTGCCGTCGGAAGCCTCACCGTCGTCAGGTGTAGCCCGCCTGCCTGGGGGCTCTTTGATCCTTCTCCGTTTTTAGCGAGGAGAAGGTAACTTTGCAGTCAAGTAATTCGAGATGGGAGAAACGCGCAGCAGGTGAGTTTGTCTGACAGTCGGTGAAACCGTTATATGTAAACAGGAACTGGCATACAGCC-358047-**Nmag_0368 (Plus strand, 358048-359319)**

1. **Nmag_0918**

**Nmag_0918 (Minus strand, 935047-937026)**-937027-ACACCGCCTTTCAAATGAATTCGGTTTAGTTCTTT

CTTTCTCACCGATCTTCGAACGAGAGGGACTCGAGTACTCCTCTCGTGTCACACGAGCGGAATACGGCAACCTAACTGTTGGATGTGGTCGATACGGCGAGACTGACACCGCATTTCAGATGAATTGGGGCCACAGTGTGGTGCTGGTTCCGCTGGTGGCTGTTGGAGAGGGGAGACCCACACCGGGTTTCAAATGATTTCGTGAGTGGATGAGGTGGGGGTCGGTGGACAGCTGAGGTGGTGGGACTCGAGTTGTGACGGCGAAGACGGGAGACCCACACCGGATTTCAGATGTAATTCCCGAGATGGCCCGGTGGTATGGTGGGGAGAAGGAGTGACGAGCTAGTGCTGTAGAACAAGGAGTGTCGAAATAGCGTTGTAGAACAAGGTGTGGTAAAATAATGCTGTAGAACAAGGGGTGACGAAATAGTGCTGTGGGAGCACGAGTAGCAGAACAGGGGAACGGCCTGATAGAAGAAGGACCTGGACAGAAGCTGTTGACGAGTGGAGAACAGCGTGTCGGTTGCCACTGAAATCATCTGAAACCCGGTGTTCCGAGTGAGTGGATCCACATCTACAATGCTTGGACAGTCATCTGGTGACATATAAATCAAACACTATTCCACTCTGTGAATTTCGGTATCCCTGTCATTGTCGTCTGTTACTTCTTTCTACTGGACTAGTAGTAGTACTAGATAACCTGTATAGTAATAGTTATAATAATGTTTCAAATTTGTCGACATCTTACCGCTCAGATATCCGCATTTGAGCGCCCCTGATGCCAATATTCGGCATCTGCGTCCGACTCTACCACAATCTCCACCTCACACCCCACCCCTTACCCCACCCCTCACGTCCGCTGCATTTCATCTGAAATCCGGTGTGGGGGCGTCAGTATTCGTCGTTCGAGTACTCCTCGTCTCGAAACAACCCTCACGGCGAAGATGACGCCGTCATTCAGAACCCTGGCTGTCGTGCCGTCTGCAAGGAGAGATCGGTAACTCTACTCGAGTACGGTTGTGCGTCCGC-938090

1. **Nmag_1930**

1960869-CTGTAGGGCCCTCCAGCACCGCGGGGTGCATCTGCGCGTACTACTGACTGAACTCACTTAAGCCTACGTCAAACGTGTTGATTTTCAGACGAAAAAGCATAGATTCCTGTCAGGTCTGGTCGCATGGTTGAGAAGAGAGTGAGTGTTGTGAGAGCGAGACGGTATGAATTAGCTTCTGGATGGACGGGTTGGCCTATATATAATCCAGAGGCAATATAGAGACGACCGCTGTGACGAGCTGAAATACCATGGGTTCGATAGAGCTAGTATTTCTCATGGGATACTAGATGTAAGTAATCGATACTAGAACACTCTAGACAAATCCTAGGTGGTCAGTTTTCGAATATGTTGGACGAATCCGACGGTGTTCGAACACCCCCACCCCTTCGTTTCAACTGGAGACCAGTGATGGAGTGGGGTTGGAGAGCTGGTGTTGAGCAGCAGTAGCGGGTTCTGTTTCAACTCGAACGGGAGTACAGACGACAACTCGGAAGAAAACGAAGGATGCGAGAAGTCTAGTAATAGATCTCCATAAGAGGGACAGATCTAGTAGCAACTAGCAATAAAACTAACTTCTCTAGTAAAAACATCAATCTGCTAGTGGTACGGTCCTTATTTCTCACCCATCATTATAAATCCTCTCGTTCTAGAAGTCACCCTCCCCCACCCCTCTCTCGACTCTCTCCAGTTGAAACGAAGGGGTGGGGGGCACCACCCCTTTCTCACACAAGTTTCCTGACTCATTGTTGGCAAGACACAATGATGATGATTCACACCCCTTCGTATCATCCCCCACACCACGCTGCATAATCTCTCACCTCACCATCTCCTCTCACCATCTCCTCTCACCAATCTTCTCTCACCAATCTACTTTCACCAGTCCACCTTCCAACTTTCCCAAAAATTCGAACTTCTGGGATCCGAACCTCCTTGAATCCGAACCTCCTGGATTGATCGACCCGAGTAGCCCACAGAAATCACCTCCACATCCACAGTCTGAGGCAGGCACGGGTCTAGGATCGGACGGAGCTGGGACAGGCATGCCCGATTCACAGCGGATCGAATCACGCATAGCTGGTTGCTGCACGAGTTGTAGCTGTCTTCTATTTTCTTCTATACGTGTGCGCGCTAGACCAACAAGATAGACACAGTGACAAGATAGACACAGTGAATACCAGTCTATCCTTTCACTGTTGGTCCAGTAGTTCAGTGTCGCAGTTGCTCACGGATTACTCGGTTCGGTGAACCACTCAACGTCCGTGAAGTGAAGGGAAGAATTTAATATCACCGTCTTCCTCTGTTTCGTTTGCATCAACTGGATCCGGACCGGGTCGTTAGGACTGTAGACTGGCCCCGCTGGCTCGCCTACACCGCTATATCCTCGCCCTCGGGTTCGGATCTGCAGTCGAAACAAGGGGGTACGTGTACGACGA-1962302-**Nmag_1930 (Plus strand, 1962303-1964261)**

1. **Nmag_2323**

**Nmag_2323 (Minus strand, 2374803-2376095)**-2376096-GCTTCCATTCTGGGAGACAGGCGTTATAAAA

CCACGGGAAAGAATTCAGACTGATAACGACCCGGAGACCGTTCCAATTCGGACGTAATTCCGCTGTAAGCGGCAGTCCGTTGGAATTCGTGTGATCAACGCCCGATCGGACAGGCCCACCCCCGATTCAGAGTGAAAGTGCGTGAAAGACGAGGGTGAGAAAAGAGCTGTGTCGGTACCGAGGTCCCGACAAAATGTAGGCGGGGCTGGGGTTCTGTCTCAGGTCTGTAGTTCTGTATCGTATTCTCCATCTCTGTTTCGTCTCGATAGAGTAACTAGTATAGAAATAGTCTATAAACAGTCTAGAAGCAGTCTAGAAGCAGTCTATAAACAGTCTAGAAGCAGTCTAGAAGCAGTCTATAAACAGTCTATAAAAATAGTATAGAAAACTAGTCTAGAAAGAGTTCTAGCTATAGTTTGAATGTTATGGTGGCGGCATGTATTTCTGGGAAGAGGGGAAGAAAAACCAGGTCACTACTACCGCTGGACACCCCTCTCTGACCTCGTTGGACCTCAGAAAGGCCGTTGCTCTCACACTTTGTACTGATTCTCGGTTTTCCGCGGACCGTTTCCCGCCTCCAGCGTCTCTCCCCCACCCCACACCACTGGGCAGATACACTCTGAACAAGGGGGGTCCCTGGGTGTCTCAACCCCACTCAAACCGACTTCCCACTTGTAGCCACTCTACAGCCTGCTACGGGGAGTTTCAAACACATCCACTACGTTCCTGGAGCAATCTGTGTGGAACCCACACAGCACCACCCCGTATTCAGAGTGAAAATCTCGTTCCTGTTTTCCGCGTCTATGCATGTCACTTTTCACTTTGAACGAGGGGTGTCTACCTTCGTCCTGGACCATCGACTTTCGGTGGTGGCGTGACGCCAGTTTCTGCCGAATCGTTTCCAGTCCGACCTCGTGGCCAGTCCCATACCTTGACATAATCGGTACGCACACAGGTGGATATGGTACCAATGGTACGACGCAGTATTCTCTTCACTCCCGGTGATCAACCGGAA-2377141

**pNMAG01 (1)**

1. **Nmag_3611^#^**

**Nmag_3611 (Plus strand, 566-1792)**-1793-GGACGGAACGGTGTTGGCTGGCGTCGACTGGTTCGGTTAG

TTGGTTCCGGTTGGCACGGTCAGCTGATCTCGGCTGATCGATCACTGTCTGGTACTCTCCCAGTAGCTATACTGATCAGTGCAAGTCACGGACGACTGTGACTTGACGGCTGGTAATCGGTGAGCAGTGTGGGGGCTCGATTCGTTGCCGGTAGCAGACGATAGCAAGACGTTCCACCGGAAATCCGGTGTCTGTCCATCGTTTTGGTTCATACCGTTCTGCGGGTAACTGTTCACCGGAAACCTGGTGTGCACTGCTAGACATCGGTGACGTTTCACCGGAAATCTGGTGTCCTGACGATCACGGTCTGGTATGGGTGTTGACAGCACCACTCATATCCTGGGCGCACACCTGATTTCCGGTGAAAGCGAACGGTAATAGTTGGCTGGGATATTCTCGGCCCTATTTTCGACTTTTGATTGCGAGCGACTCGGTTCAGGTGAACCTGCTTAACCGAGTAGTTACCACGAGACTGACCCTGGATACCACGATATCGACGTTGATCGTATGAGTATGAGGGACTCACACTTCTGTGTCAGTTTCACCGGAAACATAGTGTGAGGTACTCGTGGCTACACCTCTTCACCCAACGATGAGGAGATGGGTGTTGTGCGTCCTCGTTCAGCCTGGACGTACCAGACCCATTTGAATCAACCCGAGATGGGTGCCGGGTGAACCCGTTTCCATTCGTCTGCGA-2529

**pNMAG02 (1)**

1. **Nmag_3963**

TGGCACCGACACACCACCCGTTCAATCGTTTCATGGAGGCACCACCCACCACCCATTCATTCGTTTCACAGACATGCTGGCCAGATGGAGACACACCGGTCGTTCAATCGTTTTGCGCAGAGTGAGAGGGACCCACCACCCGTTCATTCGTTCGAGGTGAATGACGGACCCACCATCCATTCAAACGTTTTGGAAGGATTGTGCAGTGATCAAAGCGGAATACACCCACCACTGATTCATTCGTTCCAGAGACGCGGAGCAGAATGTGGATCGTTCACGGAGCAGAATGTGGAGGACACACCACCCGTTCAAACGTTAGTGGGTGTTCCAGAGGGAGAGAGACACCACCCATTCAATCGTTCTGGTGAAATAGCCCCAAGTGTCGATACCGGATATAAGGGAAGGGATTTAGCGGGTAGAAGGACTGTGCTGTACGAAACCGAAGGAAGAGGGTTTTGAGGAGCAGAGAAGGACCGATACCAGAACCAAACACACCACCCATTCAAACGTTTTCTGTGGTACTGCAGTGCGGGTATGGTTTTGACGCTATCATTATAAAACTAGGCCTGTTCCAAAACCGATTCACAGTATCTCAGGAAGCTAGGGGGACTCTCGGAGGGGTTAACGATTGAATCAGTGGTGTGTGTGTGTGTTCGGGAACGAATGAAACGGAGGAATCGTTTGTAACGATTGCGCCTATGGTTTT-148-**Nmag_3963 (Plus strand,** 149-1411**)**

**14. *Natronomonas pharaonis* DSM 2160** (CR936257-59)

**Chromosome (2)**

1. **Cdc6_1**

302012-ATCATCCTCCCACGCCCCCGATTTCGAGTCGAAATCGGAGTCGCTCTGCAGATACTACCATCCTGAACTCACTTAAACATATGTCAGACATCAGCCTACCGGTTCCGTAGCCGGTCGGCTTTGGGTCGAGCTGGGCCGGACCAGATGGGGCGGAGCCAAGCATGGGACCAAGGAGGGGCGGGCCACTCCGTTCGTCTTCGTCTGTCGGCAGGCCCACCTGTCTTTGTCTGCCGACAGGCCTCTGGGACAGCAGGACCGTATCCCTGCCGCACCCCCCCACCCCTTCGTTTCAACTGGAGCGGAGAAATGGAGGGGGAGGGGGAGCGCTGGAGTGGGTGAACTCATAGTAGAATAAATAGCGTTTAACGTAGCTACTACTGTACTCTCTGGAATAACCGAGGAAGAGTGTATCGGTAAGCTAGTTTAGATTGGAGACCTAGCCGGGATAGACTAGATATGCTGCTGTATCTAGTACTCACTAGTAATTTTCTACTACTACTAGCAACATAGCGAAACACAGCGAAACACAGCGATTACTAGCCTATAACAACCATAGAAATATAAACAGAATCGTATTTCTTTGTCGTACGGATTTGGTTACTTCCTACAGGTAATAAAGGTTTCTCAACTAGAACGCCTCTCGTCTACCCCTCCCCACCCCTTTCAACGGCTCCAGTCGAAACGAAGGGGTGTGGGGGTAACCACCCTTGTTTCGTCTGCATCAACTGGAGATGTGACTTTCCCGACTTTCCTGGCCCGCTGGATTCTCTCGACCCCACTCTCTCGTTCCCCGTGTTTCACGTCGCCGCCCCCTCGGCACTGGCCCAGCGTCTCGGCGTCCGGTCTCTCAGCATCCGACTTGTTTCTCGGCGTCCAATTCCGTCGGCGTCCAATTCCAGTGCCGATATCCGAGTTCAGTGTCCGTCAGAGTTCAGTGTCCGTCAGATATTATGGCGGAGCTCCCGCCCGGTGGGGGGCTTCGGTCGGCACGTCGGCGTCCGGGTGCTCGAAGTCGGGGCTCCCAATCTCTTTGCCGACCTCATCAAAAAACTTAATACCGAACTCGACCATCGGTTCGACTGCATCAAGTGGACATCCGTTTGCACGCCCTCCACCGAATCAGGGCCTCTACCCCCTGTATTCTTTCCATTCGCTCGATGCGGGGTGGTTTTCCAATTGAAACGGTGGGGTACAGCGGAGACACGCCGCAGGGGAACACAGA-303233-**Cdc6_1 (Plus strand, 303234-304949)**

1. **Cdc6_3**

1486980-CTGGACTGGAGTATGCTGCTTTCCTGTGTTCGTCGCTGCAGCGTCTCGTTCTGTGCTTCCCGTGGTGCGGTTAGGAGTCTGTGGTGTGGTCGGTAGTCTGTGGTGTGGTCAGTAGTCCGCAGCGCTGCCGGTGTACGGACACCCCTCATTTCGTCTGTTCTTGGCAATGGAGGGGTACCGTATTCGGATGGGACGTGGGGTTGATGCGCAGGCCCACACCCCTCGTTTCGTCTGTTCTTCTCAAAAGAAGAGGGAGAGAAGTGGAGGAGAAAGAAGTTGAGGGAGAGAAGTGGAGGGAGAAAAGGAGAGGAAGAAAAAGAGATAGAGAAGGGAAGGAAGTGGAGAAAAAGAGGAGGGTGAGAGAATGCAAGGGAAACAGAGCGGGAGCAAGATGGAACAAAAAGACGAGGAGGACACCAAAGAGCTGAAAGGGCAAAAAAGAAAAACGAAAACAGCTTCATACGAAACACCCGAAACAGACGAACGCTACTTTTATTAGGTGCCGTTCTGTTTGCGACCTTACGTGAG-1487507-**Cdc6_3 (Plus strand, 1487508-1488704)**

**pL131 (1)**

1. **Cdc6_5**

94739-GACTACGCGTCCTCGCCGGGCTGAGACTACGTGTCCCCGCCGGGGGCTACCGCTCCACCGTGGAAGCGAGCCTTCGACGCAGTACTGGCAGCGCGTGTTTTAACCAACACTATGCCGTTTTGTTGGTTAATGGCGGAATGGTCGGTGAGCGGATTTACACATCGATAGAGGGGTCTGGCTGCTCCGCGGGACCGGAATTAATGGATTCAAGGGTCCCGGGGATTGACTTCGGGGCAGTCCACAGAACAAAAACCAACTCGACGTCGAGTCTCAAGGCGACTAGTTTCACTTAGCTCGCTGCTGTTGGCATGGTTCCTCTCTGTGGACGTCGGGGACAGACACCCCTCTATCGATGTGTTCTATCTGTCGAGAGGAAGTAGAGCTGTGCCAGCGGAGAGATTACGATAGCCACGAAACGCACGATACAGGACGACACAAGAGCGTATTCACGGAGAAACTGGTATTCCATGGGGGGAGACGAATATCTCAGAGCCATACACATCGAACACATCGATAGAGGGGTGTGTGAGTGTTCTTCTAGAAACTAGAAGAAGAAAGAAGAAGAAACAACACTACAACCTAGTTAAAATATGGCCCGTCTAGAACAAGCATCTAGAAAACAGCGGTTTTAGACGCTAACAAAGGTCTTCTTGATATCGAGTGCAGCAAGACGGTCGATTCAACCCCCACCCTACCAACTCTTCGAACACATCGATAGAGGGGTGTCTGTCTTGTAGTGGTCTCTCGACAGCATACGATAGCACCGACGATGGCGTTCCTTCGTGTTTCGATGGGCCGGCTGGCTCGGTCGCCCAAGCAGCCAACGTAGGACACGAGTGCGTCCTTGTTGGCTAAGCAAGCTCCGCCCGCTCCAGGCGACGGTGTACTTCGCGTCGGCGGTATTCGAGGAGCCGCATCAATCCACGCTTCGTGTTGCTTCTCCCAAAACACTTCGTCGGACGCGTTGTTCTTTTGGCGGCTATTTCCAGTTGAACCAGCGCATCTATGGCCTTATACGCTTTACAAACGGCGATTACACTTCGATAGAGGGGTGCGAAGCTTTTTGTAAGCCGCCTGTGTGAGTCGTCGT-95828-**Cdc6_5 (Plus strand, 95829-97298)**
