# Supplementary material for: Diversity and evolution of multiple orc/cdc6-adjacent replication origins in haloarchaea
Source: BMC Genomics. 2012 Sep 14;13:478. doi: 10.1186/1471-2164-13-478 (PMC3528665; doi:10.1186/1471-2164-13-478)
Supplement: Additional file 6 — List of genes in the divergent regions between H. hispanica and H. marismortui. Blast analysis of the genes in the divergent regions between H. hispanica and H. marismortui. The genes whose closest relative is outside haloarchaea are highlighted in different colors. [file 1471-2164-13-478-S6.doc]

**Additional file 6: List of genes on the divergent regions in *H. hispanica* and *H. marismortui*.**

**Table 1. List of genes on Hh**is_A region

| ORF | Description | Best BLAST hit | | |
| --- | --- | --- | --- | --- |
|  |  | Accession | % identity | Taxonomic affiliation |
| HAH_1657 | hypothetical protein | [NP_070307](http://www.ncbi.nlm.nih.gov/protein/11499073?report=genbank&log$=prottop&blast_rank=1&RID=Y9BJAW1N016) | 56 | *Halorhabdus tiamatea* |
| HAH_1658 | transposase | [YP_135507](http://www.ncbi.nlm.nih.gov/protein/55377657?report=genbank&log$=prottop&blast_rank=1&RID=Y9BJAW1N016) | 99 | *Haloarcula marismortui* |
| HAH_1659 | transposase | [YP_135506](http://www.ncbi.nlm.nih.gov/protein/55377656?report=genbank&log$=prottop&blast_rank=1&RID=Y9BJAW1N016) | 98 | *Haloarcula marismortui* |
| **HAH_1660** | **orc/cdc6 family replication initiation protein** | [**YP_003129991**](http://www.ncbi.nlm.nih.gov/protein/257052158?report=genbank&log$=prottop&blast_rank=1&RID=Y9BJAW1N016) | **80** | ***Halorhabdus utahensis*** |
| HAH_1661 | LPS glycosyltransferase | [YP_004044356](http://www.ncbi.nlm.nih.gov/protein/313117373?report=genbank&log$=prottop&blast_rank=1&RID=Y9BJAW1N016) | 76 | *Natrinema pellirubrum* |
| HAH_1662 | LPS glycosyltransferase | [YP_004519752](http://www.ncbi.nlm.nih.gov/protein/333987145?report=genbank&log$=prottop&blast_rank=1&RID=Y9BJAW1N016) | 48 | *Methanobacterium* sp. SWAN-1 |
| HAH_1663 | glycosyltransferase | [YP_447113](http://www.ncbi.nlm.nih.gov/protein/84488881?report=genbank&log$=prottop&blast_rank=1&RID=Y9BJAW1N016) | 41 | *Methanosphaera stadtmanae* |
| HAH_1664 | hypothetical protein | [YP_003129982](http://www.ncbi.nlm.nih.gov/protein/257052149?report=genbank&log$=prottop&blast_rank=1&RID=Y9BJAW1N016) | 29 | *Halorhabdus utahensis* |
| HAH_1665 | polysaccharide biosynthesis protein | [YP_004341291](http://www.ncbi.nlm.nih.gov/protein/327400452?report=genbank&log$=prottop&blast_rank=1&RID=Y9BJAW1N016) | 32 | *Archaeoglobus veneficus* |
| HAH_1666 | arylsulfatase A family protein | [YP_004044354](http://www.ncbi.nlm.nih.gov/protein/313117371?report=genbank&log$=prottop&blast_rank=1&RID=Y9BJAW1N016) | 48 | *Halogeometricum borinquense* |
| HAH_1667 | hexosyltransferase; glycosyltransferase | [YP_659211](http://www.ncbi.nlm.nih.gov/protein/110669400?report=genbank&log$=prottop&blast_rank=1&RID=Y9BJAW1N016) | 67 | *Haloquadratum walsbyi* |
| HAH_1668 | hypothetical protein | [YP_003480900](http://www.ncbi.nlm.nih.gov/protein/289582434?report=genbank&log$=prottop&blast_rank=1&RID=Y9BJAW1N016) | 72 | *Natrialba magadii* |
| HAH_1669 |  |  |  |  |
| HAH_1670 | hypothetical protein | [YP_003480902](http://www.ncbi.nlm.nih.gov/protein/289582436?report=genbank&log$=prottop&blast_rank=1&RID=Y9BJAW1N016) | 83 | *Natrialba magadii* |
| HAH_1671 |  |  |  |  |
| HAH_1672 |  |  |  |  |
| HAH_1673 | glycosyltransferase | [YP_004044356](http://www.ncbi.nlm.nih.gov/protein/313117373?report=genbank&log$=prottop&blast_rank=1&RID=Y9BJAW1N016) | 55 | *Natrinema pellirubrum* |
| HAH_1674 |  |  |  |  |

The origin-associated *orc/cdc6* genes are indicated in bold. Genes color-coded with blue represent closest relationship with Methanobacterium or other non-halophilic archaea in BLAST searches.

**Table 2. List of genes on Hmar_A region**

| ORF | Description | Best BLAST hit | | |
| --- | --- | --- | --- | --- |
|  |  | Accession | % identity | Taxonomic affiliation |
| rrnAC1048 | hypothetical protein | [YP_003738572](http://www.ncbi.nlm.nih.gov/protein/300712759?report=genbank&log$=prottop&blast_rank=2&RID=A25XUMFU01N) | 63 | *Halalkalicoccus jeotgali* B3 |
| rrnAC1049 | transcription regulator | [ZP_08561166](http://www.ncbi.nlm.nih.gov/protein/335438422?report=genbank&log$=prottop&blast_rank=2&RID=A25XUMFU01N) | 56 | *Halorhabdus tiamatea* |
| rrnAC1050 | hypothetical protein | [YP_657884](http://www.ncbi.nlm.nih.gov/protein/110668073?report=genbank&log$=prottop&blast_rank=2&RID=A25XUMFU01N) | 60 | *Haloquadratum walsbyi* |
| rrnAC1051 | hypothetical protein | [ZP_08964487](http://www.ncbi.nlm.nih.gov/protein/352116522?report=genbank&log$=prottop&blast_rank=3&RID=A25XUMFU01N) | 66 | *Natrinema pellirubrum* |
| rrnAC1052 | hypothetical protein | [ZP_08964508](http://www.ncbi.nlm.nih.gov/protein/352116543?report=genbank&log$=prottop&blast_rank=2&RID=A25XUMFU01N) | 38 | *Natrinema pellirubrum* |
| **rrnAC1053** | **orc/cdc6 family replication initiation protein** | [**YP_003736848**](http://www.ncbi.nlm.nih.gov/protein/300711034?report=genbank&log$=prottop&blast_rank=4&RID=A25XUMFU01N) | **52** | ***Halalkalicoccus jeotgali* B3** |
| rrnAC1054 | transposase | [ZP_08969569](http://www.ncbi.nlm.nih.gov/protein/352125715?report=genbank&log$=prottop&blast_rank=2&RID=A25XUMFU01N) | 76 | *Natronobacterium gregoryi* |
| rrnAC1055 | hypothetical protein | [ZP_08562201](http://www.ncbi.nlm.nih.gov/protein/335441532?report=genbank&log$=prottop&blast_rank=2&RID=A25XUMFU01N) | 97 | *Halorhabdus tiamatea* |
| rrnAC1056 | hypothetical protein | [ZP_08558714](http://www.ncbi.nlm.nih.gov/protein/335433902?report=genbank&log$=prottop&blast_rank=2&RID=A25XUMFU01N) | 72 | *Halorhabdus tiamatea* |
| rrnAC1057 | plasmid stability protein | [ZP_08558715](http://www.ncbi.nlm.nih.gov/protein/335433903?report=genbank&log$=prottop&blast_rank=2&RID=A25XUMFU01N) | 48 | *Halorhabdus tiamatea* |
| rrnAC1059 | hypothetical protein | [YP_002564325](http://www.ncbi.nlm.nih.gov/protein/222475804?report=genbank&log$=prottop&blast_rank=2&RID=A25XUMFU01N) | 39 | *Halorubrum lacusprofundi* |
| rrnAC1060 | hypothetical protein | [ZP_08558300](http://www.ncbi.nlm.nih.gov/protein/335433476?report=genbank&log$=prottop&blast_rank=4&RID=A25XUMFU01N) | 33 | *Halorhabdus tiamatea* |
| rrnAC1061 | transfer complex protein | [YP_003130594](http://www.ncbi.nlm.nih.gov/protein/257052761?report=genbank&log$=prottop&blast_rank=3&RID=A25XUMFU01N) | 69 | *Halorhabdus utahensis* |
| rrnAC1062 | hypothetical protein | [YP_003130593](http://www.ncbi.nlm.nih.gov/protein/257052760?report=genbank&log$=prottop&blast_rank=3&RID=A25XUMFU01N) | 51 | *Halorhabdus utahensis* |
| rrnAC1063 | hypothetical protein | [YP_003130592](http://www.ncbi.nlm.nih.gov/protein/257052759?report=genbank&log$=prottop&blast_rank=3&RID=A25XUMFU01N) | 57 | *Halorhabdus utahensis* |
| rrnAC1064 | hypothetical protein | [YP_003178047](http://www.ncbi.nlm.nih.gov/protein/257388274?report=genbank&log$=prottop&blast_rank=2&RID=A25XUMFU01N) | 81 | *Halomicrobium mukohataei* |

The origin-associated *orc/cdc6* genes are indicated in bold.

**Table 3. L**ist of genes on Hhis_B region

| ORF | Description | Best BLAST hit | | |
| --- | --- | --- | --- | --- |
|  |  | Accession | % identity | Taxonomic affiliation |
| HAH_2114 | hypothetical protein | NP_045946 | 81 | *Halobacterium* sp. NRC-1 |
| HAH_2115 | glycosyltransferase | [ZP_05283216](http://www.ncbi.nlm.nih.gov/protein/255011090?report=genbank&log$=prottop&blast_rank=1&RID=YB6B76H401S) | 41 | *Bacteroides fragilis* |
| HAH_2116 | hypothetical protein | YP_003536073 | 47 | *Haloferax volcanii* |
| HAH_2117 | glycosyltransferase | [YP_001688306](http://www.ncbi.nlm.nih.gov/protein/169235106?report=genbank&log$=prottop&blast_rank=1&RID=YB6B76H401S) | 38 | *Halobacterium salinarum* R1 |
| HAH_2118 | arylsulfatase | ZP_08046010 | 32 | *Haladaptatus paucihalophilus* |
| HAH_2119 | transposase | NP_046052 | 83 | *Halobacterium* sp. NRC-1 |
| HAH_2120 | transposase | YP_003177286 | 94 | *Halomicrobium mukohataei* |
| HAH_2121 | glycosyl transferase group 1 | YP_003130892 | 31 | *Halorhabdus utahensis* |
| HAH_2122 | hexosyltransferase; glycosyltransferase | YP_659205 | 45 | *Haloquadratum walsbyi* |
| HAH_2123 | hypothetical protein | YP_002430497 | 32 | *Desulfatibacillum alkenivorans* |
| HAH_2124 | glycosyl transferase group 1 | ZP_06369153 | 30 | *Desulfovibrio* sp. |
| HAH_2125 | glycosyl transferase group 1 | YP_001804214 | 40 | *Cyanothece* sp. |
| HAH_2126 | O-methyltransferase-like protein | ZP_01891901 | 39 | unidentified eubacterium |
| HAH_2127 | export protein | ZP_08045397 | 38 | *Haladaptatus paucihalophilus* |
| HAH_2128 | transposase | YP_001688265 | 87 | *Halobacterium salinarum* R1 |
| HAH_2129 | transposase | YP_001688265 | 85 | *Halobacterium salinarum* R1 |
| HAH_2130 | hypothetical protein | YP_002564250 | 54 | *Halorubrum lacusprofundi* |

Genes color-coded with red represent closest relationship with bacteria in BLAST searches.

Table 4. List of genes on Hmar_B region

| ORF | Description | Best BLAST hit | | |
| --- | --- | --- | --- | --- |
|  |  | Accession | % identity | Taxonomic affiliation |
| rrnAC1544 | transcription regulator | YP_003129892 | 48 | *Halorhabdus utahensis* |
| rrnAC1545 | hypothetical protein | YP_003129891 | 44 | *Halorhabdus utahensis* |
| rrnAC1546 | transposase | YP_003537089 | 89 | *Haloferax volcanii* |
| rrnAC1547 | hypothetical protein | YP_003535780 | 78 | *Haloferax volcanii* |
| rrnAC1548 | hypothetical protein | YP_003535779 | 76 | *Haloferax volcanii* |
| rrnAC1549 | hypothetical protein |  |  |  |
| rrnAC1550 | hypothetical protein |  |  |  |
| rrnAC1551 | hypothetical protein |  |  |  |
| rrnAC1552 | hypothetical protein | [ZP_08964495](http://www.ncbi.nlm.nih.gov/protein/352116530?report=genbank&log$=prottop&blast_rank=2&RID=A28Z5PDT01N) | 86 | *Natrinema pellirubrum* |
| rrnAC1553 | zinc finger SWIM domain-containing protein | [ZP_08964495](http://www.ncbi.nlm.nih.gov/protein/352116530?report=genbank&log$=prottop&blast_rank=2&RID=A28Z5PDT01N) | 88 | *Natrinema pellirubrum* |
| rrnAC1555 | zinc finger SWIM domain protein | [ZP_08964495](http://www.ncbi.nlm.nih.gov/protein/352116530?report=genbank&log$=prottop&blast_rank=2&RID=A28Z5PDT01N) | 88 | *Natrinema pellirubrum* |
| rrnAC1556 | hypothetical protein | [ZP_08964494](http://www.ncbi.nlm.nih.gov/protein/352116529?report=genbank&log$=prottop&blast_rank=2&RID=A28Z5PDT01N) | 86 | *Natrinema pellirubrum* |
| rrnAC1557 | hypothetical protein | YP_003735727/  [ZP_08562014](http://www.ncbi.nlm.nih.gov/protein/335441302?report=genbank&log$=prottop&blast_rank=3&RID=A28Z5PDT01N) | 83 | *Halalkalicoccus jeotgali* B3/  *Halorhabdus tiamatea* |
| rrnAC1558 | hypothetical protein | [ZP_08562013](http://www.ncbi.nlm.nih.gov/protein/335441301?report=genbank&log$=prottop&blast_rank=2&RID=A28Z5PDT01N) | 86 | *Halorhabdus tiamatea* |
| rrnAC1559 | transposase | [ZP_08964506](http://www.ncbi.nlm.nih.gov/protein/352116541?report=genbank&log$=prottop&blast_rank=2&RID=A28Z5PDT01N) | 91 | *Natrinema pellirubrum* |
| rrnAC1560 | transposase | YP_657788 | 91 | *Haloquadratum walsbyi* |
| rrnAC1561 | hypothetical protein | NP_279188 | 73 | *Halobacterium* sp. NRC-1 |
| rrnAC1562 | hypothetical protein | NP_279190 | 37 | *Halobacterium* sp. NRC-1 |
| rrnAC1563 | hypothetical protein | NP_279191 | 79 | *Halobacterium* sp. NRC-1 |
| rrnAC1564 | hypothetical protein |  |  |  |
| rrnAC1565 | transposase | NP_279201 | 75 | *Halobacterium* sp. NRC-1 |
| rrnAC1566 | hypothetical protein | YP_657884 | 67 | *Haloquadratum walsbyi* |
| rrnAC1567 | hypothetical protein | YP_657885 | 88 | *Haloquadratum walsbyi* |
| **rrnAC1568** | **cell division control protein 6** | **YP_004038327** | **56** | ***Halogeometricum borinquense*** |
| rrnAC1569 | cell division control protein 6-like protein | [YP_004598664](http://www.ncbi.nlm.nih.gov/protein/336255557?report=genbank&log$=prottop&blast_rank=2&RID=A28Z5PDT01N) | 71 | *Halopiger xanaduensis* |
| rrnAC1570 | ABC transporter ATP-binding protein | YP_003177280 | 69 | *Halomicrobium mukohataei* |
| rrnAC1571 | GDP-mannose mannosyl hydrolase | YP_004044342 | 51 | *Halogeometricum borinquense* |
| rrnAC1572 | dTDP-glucose-46-dehydratase | ZP_05092212 | 39 | *Carboxydibrachium pacificum* |
| rrnAC1573 | UDP-glucose 4-epimerase | YP_002462880 | 61 | *Chloroflexus aggregans* |
| rrnAC1574 | Transposase | [ZP_08968237](http://www.ncbi.nlm.nih.gov/protein/352124057?report=genbank&log$=prottop&blast_rank=2&RID=A28Z5PDT01N) | 34 | *Natronobacterium gregoryi* |
| rrnAC1575 | transposase | [ZP_08968874](http://www.ncbi.nlm.nih.gov/protein/352124808?report=genbank&log$=prottop&blast_rank=2&RID=A28Z5PDT01N) | 92 | *Natronobacterium gregoryi* |
| rrnAC1576 | hypothetical protein | ZP_07202326 | 32 | *delta proteobacterium* |
| rrnAC1577 | transposase | YP_003537079 | 85 | *Haloferax volcanii* |
| rrnAC1578 | transposase | [YP_004785917](http://www.ncbi.nlm.nih.gov/protein/344209740?report=genbank&log$=prottop&blast_rank=2&RID=A28Z5PDT01N) | 91 | *Haloarcula hispanica* |
| rrnAC1579 | transposase | [ZP_08559152](http://www.ncbi.nlm.nih.gov/protein/335436356?report=genbank&log$=prottop&blast_rank=2&RID=A28Z5PDT01N) | 43 | *Halorhabdus tiamatea* |
| rrnAC1580 | transposase | YP_001688280 | 98 | *Halobacterium salinarum* R1 |
| rrnAC1581 | transposase | YP_001688280 | 100 | *Halobacterium salinarum* R1 |
| rrnAC1582 | hypothetical protein | [YP_004043072](http://www.ncbi.nlm.nih.gov/protein/313204415?report=genbank&log$=prottop&blast_rank=2&RID=A28Z5PDT01N) | 26 | *Paludibacter propionicigenes* |
| rrnAC1583 | LPS glycosyltransferase | ZP_05023801 | 29 | *Microcoleus chthonoplastes* |
| rrnAC1584 | LPS biosynthesis protein | YP_001688308 | 61 | *Halobacterium salinarum* R1 |
| rrnAC1585 | glucose-1-phosphate thymidylyltransferase | [YP_003735721](http://www.ncbi.nlm.nih.gov/protein/300709907?report=genbank&log$=prottop&blast_rank=3&RID=A28Z5PDT01N) | 84 | *Halalkalicoccus jeotgali* B3 |
| rrnAC1586 | hypothetical protein | [YP_004795101](http://www.ncbi.nlm.nih.gov/protein/344210781?report=genbank&log$=prottop&blast_rank=2&RID=A28Z5PDT01N) | 74 | *Haloarcula hispanica* |
| rrnAC1587 | glucosamine-fructose-6-phosphate aminotransferase | YP_004795100 | 97 | *Haloarcula hispanica* |
| rrnAC1588 | transposase | YP_003481298 | 88 | *Natrialba magadii* |

The origin-associated *orc/cdc6* genes are indicated in bold. Genes color-coded with red represent closest relationship with bacteria in BLAST searches.
